# Multiomic spatial analysis reveals a distinct mucosa-associated virome

**DOI:** 10.1080/19490976.2023.2177488

**Published:** 2023-02-23

**Authors:** Austin Yan, James Butcher, Laetitia Schramm, David R. Mack, Alain Stintzi

**Affiliations:** a Ottawa Institute of Systems Biology, Department of Biochemistry, Microbiology, and Immunology, Faculty of Medicine, University of Ottawa, Ottawa, ON, Canada; b Department of Pediatrics, Faculty of Medicine, University of Ottawa, Ottawa, ON, Canada; c Inflammatory Bowel Disease Centre and CHEO Research Institute, Division of Gastroenterology, Hepatology and Nutrition, Children’s Hospital of Eastern Ontario, Ottawa, ON, Canada

**Keywords:** Virome, bacteriophages, gut microbiome, phageome, gut mucosa

## Abstract

The human gut virome has been increasingly explored in recent years. However, nearly all virome-sequencing efforts rely solely on fecal samples and few studies leverage multiomic approaches to investigate phage–host relationships. Here, we combine metagenomics, metaviromics, and metatranscriptomics to study virome-bacteriome interactions at the colonic mucosal-luminal interface in a cohort of three individuals with inflammatory bowel disease; non-IBD controls were not included in this study. We show that the mucosal viral population is distinct from the stool virome and houses abundant crAss-like phages that are undetectable by fecal sampling. Through viral protein prediction and metatranscriptomic analysis, we explore viral gene transcription, prophage activation, and the relationship between the presence of integrase and temperate phages in IBD subjects. We also show the impact of deep sequencing on virus recovery and offer guidelines for selecting optimal sequencing depths in future metaviromic studies. Systems biology approaches such as those presented in this report will enhance our understanding of the human virome and its interactions with our microbiome and our health.

## Summary

The human gut hosts many bacteria-infecting viruses, also known as phages. These phages interact with other gut microbes and affect human health, yet they are understudied. We advance the study of human gut phages on three frontiers by studying gut viruses in three patients with bowel diseases. First, nearly all phage studies use fecal sampling; however, these phages may be transient and are less relevant to our health. By obtaining samples during colonoscopy, we studied phages from the colonic wall and show that they are different than those found in our stool. Second, we use RNA sequencing to explore how phages interact with bacteria within a complex microbial ecosystem. By applying these new techniques, we advance our study from “what phages are there?” to “what are they doing?” Finally, this study reports one of the most extensive per-sample sequencing efforts to date, allowing us to make practical recommendations for other researchers aiming to design phage studies. Further studies will expand our understanding of our gut phages and how they interact with human health.

## Introduction

The human gut virome, which encompasses the vast and diverse collection of viruses in our gastrointestinal tract, has been linked to several human diseases including inflammatory bowel disease, cancer, and diabetes, and may impact the efficacy of microbiome-modulating therapies.^1–3^ Our gut viromes are highly individualized, temporally stable, and consist mainly of double-stranded DNA bacteriophages from the *Caudovirales* order.^4^

Virome research has lagged behind its bacterial counterpart due to difficulties in sample processing, unannotated viral “dark matter”, and high viral heterogeneity. Yet recent advances have led to the exploration of virome assembly and development in neonates, a significant expansion of viral databases, and the characterization and isolation of several crAss-like phages which were only first described in 2014.^5–9^ Most studies rely either on the sequencing of virus-like particle (VLP) enriched metagenomes, herein referred to as metaviromes, or the identification of viral sequences within whole metagenomes (also referred to as bulk or whole-community metagenomes). Each approach captures a distinct subset of the virome community and, when used together, can increase the recovery of viral species and provide additional context about viral populations.^6,10,11^ Paired metagenomic and metaviromic sampling also enables the study of interactions between viral species and their bacterial hosts.^10^ Efforts to integrate other experimental technologies, including metatranscriptomics and metaproteomics, are also evolving, bringing a systems biology approach to viromics and its transkingdom interactions.^1,12^

To date, metatranscriptomic approaches have been rarely applied in the context of human metaviromics. This may be due to the additional technical challenges of obtaining high-quality RNA, the lack of procedures to enrich for viral transcripts, and limited viral sequence databases. Oral viromes have been investigated using RNA-seq; one study was able to map 30% of the reads to viral populations, but lacked the sequencing depth for further downstream analysis such as assembly.^13^ Data mining efforts in the past few years have also identified ssRNA phages in activated sludge and aquatic environments, significantly expanding the number of known ssRNA phage genomes.^14^ Due to the challenge of studying viromes at the community level, transcriptomic analysis has thus been primarily used for more focused phage-host studies such as phage profiling of *Salmonella* and *Yersinia*,^15,16^ and more recently ΦcrAss001 and its host *Bacteroides intestinalis*.^17^

Furthermore, most of our understanding of the “gut” virome is based on fecal viromes, which, like the bacteriome, likely do not represent the complex biogeography of our gastrointestinal tract.^18^ A recent profiling of gastrointestinal tract viromes in the domestic pig and rhesus macaque show that virome composition was specific to anatomical region, with differences in viral load and diversity between luminal and mucosal samples.^19^ The mucosal virome is hypothesized to have different ecological pressures than the primarily luminally derived stool virome and may have greater influence over the host-associated bacteriome.^3,20–22^ Rectal samples have been used to study viromes in patients with ulcerative colitis while a recent study utilized colon resections and ileostomy fluid.^23,24^ These human studies, however, did not compare the virome across multiple sites or compare colonic sampling to fecal viromes.

We recently demonstrated the use of mucosal-luminal interface samples to study the gut virome at specific sites within the gastrointestinal tract.^11^ This sampling technique also provides sufficient microbial content for matched whole metagenomic and metatranscriptomic shotgun sequencing. Here, we leverage these advantages to demonstrate a multiomic spatial characterization of the virome in three treatment-naïve, pediatric patients with ulcerative colitis, an inflammatory bowel disease. We also highlight the power of matched metatranscriptomic sequencing to reveal viral gene transcription to better understand virome-bacteriome interactions. We did not seek to compare the IBD or ulcerative colitis virome to non-IBD controls in this study.

## Results and Discussion

### Multiomic sequencing of the mucosal-luminal interface microbiome

Samples were obtained from three pediatric patients at the Children’s Hospital of Eastern Ontario in Ottawa, Canada (Table 1). These participants (hereafter referred to as Participants F, G, and H) were treatment-naïve patients undergoing colonoscopy for confirmation of their clinically-suspected ulcerative colitis. Washes of the mucosal-luminal interface (MLI) from the proximal colon (PC) and distal colon (DC) were collected, along with a stool (STL) sample that was obtained between 2 days prior to endoscopy and 20 days after endoscopy. All samples were processed for metavirome and whole metagenome sequencing as previously described.^11^ MLI samples were also subjected to metatranscriptome sequencing; H-DC had insufficient quality after library preparation and was not sequenced. After removal of low-quality, low-complexity, and host reads, a mean of 211 (ranging from 157 to 254) million paired-end metavirome reads were obtained per sample, providing one of the deepest metavirome sequencing efforts to date. Whole metagenome sequencing yielded an average of 75.4 (6.83–162) million paired-end reads per sample while metatranscriptomic sequencing yielded an average of 119 (107–139) million reads per sample.

**Table 1.** Participant and sample descriptions.

Participant	Sex	Age (years)	Paris Score	Site	Site Mucosal Inflammation/Stool description
F	Female	17.3	E4 S1	PC	Mayo UC – Grade 2
				DC	Mayo UC – Grade 2
				STL	Pre-scope (two days)
G	Male	14.5	E2 S0	PC	Mayo UC – Grade 0
				DC	Mayo UC – Grade 1
				STL	Post-scope (twenty days)
H	Male	16.1	E3 S0	PC	Mayo UC – Grade 0
				DC	Mayo UC – Grade 2
				STL	Pre-scope (one day)

Abbreviations: PC: proximal colon; DC: distal colon; STL: stool.

As noted previously, whole metagenome sequencing at the mucosal-luminal interface is prone to high host contamination at inflamed sites (Supplementary Figure 1A).^11^ Host contamination has been suggested as a possible biomarker for inflammation, reflecting epithelial and blood cells that are more likely shed in intestinal diseases.^25^ In this study, all aspirates taken from sites of Mayo Score 2, which is indicative of moderate disease, had >69.8% host contamination.^26^ In contrast, aspirates from non-inflamed sites (Mayo 0) or mildly inflamed sites (Mayo 1) had no more than 3.7% host contamination. Similarly, host content in the stool metagenomes was markedly increased if Mayo Grade 2 inflammation in the proximal or distal colon was observed. These results suggest that moderate inflammation (Mayo 2) which includes erosions, complete loss of vascular pattern, and significant erythema but not mild inflammation (Mayo 1) is correlated with increased host content. Furthermore, both MLI and stool sampling are affected by mucosal inflammation.

Bacterial contamination in the metavirome sequencing data was assessed by aligning reads against a *cpn60* housekeeping gene database.^27^ Metavirome samples had a mean 172-fold decrease of mapped *cpn60* reads compared to their whole metagenomic counterparts (Supplementary Figure 1B), demonstrating efficient viral purification in this dataset consistent with prior metavirome studies.^11,28^

### Viral contig identification across multiomic datasets

High-quality, host-removed sequencing reads were assembled per sample and clustered (Supplementary Figure 2). Using Cenote-Taker2, we identified 1,880 putative viral contigs (VCs) from the metavirome, and an additional 785 VCs from the whole metagenome, including 242 pruned sequences with flanking host regions. These putative VCs were pooled and further clustered, resulting in a set of 2,171 VCs, of which 330 were present in both the metagenome and metavirome datasets. Most VCs (1,499) were only assembled from the metavirome, while 342 were only found in the metagenome. By integrating metagenomics and metaviromics, we could infer bacteriophage lysogeny by the presence of bacterial flanking regions. VCs were defined as integration-capable (IC-VCs) if their cluster included a metagenome-derived VC with pruned flanking regions,^9^ or a virome-derived VC that could be clustered within a non-viral contig (296 of 2,171 VCs). These 296 IC-VCs were linked to 309 non-viral contigs representing their host genomes. Of these IC-VCs, 105 were only identified from the metagenome and were thus likely inactive (i.e. prophages), while the remaining 191 were also present in the metavirome, suggesting simultaneous prophage activation as temperate phages. The other 1,875 VCs are likely primarily composed of free phages but could also include pseudolysogenic phages; here, we define these phages as non-integrated VCs (NI-VCs). While NI-VCs may be capable of phage integration, they were not detected as prophages in our study.

Viral contigs were assessed for quality using CheckV and annotated using Demovir and vContact2.^29–31^ 94.5% of VCs (2,051/2,171) could be annotated at the viral order level (Table 2) and primarily represented the order *Caudovirales* (n = 1,892). The remaining VCs were mainly *Microviridae* and *Circoviridae*, which were predominantly identified from metavirome sequencing. Non-viral contigs in the metagenome (n = 39,735) were taxonomically annotated and subsequently used for phage-host analysis. High-quality sequencing reads across all three datasets were mapped to the set of viral and non-viral contigs; counts were normalized for contig length, resulting in a normalized relative abundance (NRA). In all, 86.4–98.3% of metagenome reads, 67.3–97.6% of transcriptome reads, and 88.9–98.2% of metavirome reads mapped to a contig; 4.74–11.8%, 0.96–2.52%, and 52.9–91.8% of reads mapped to viral contigs, respectively. Metagenome and metaviromic datasets were further filtered for a minimum breadth of coverage of 75% with ≥1 read per bp for subsequent analysis.

**Table 2.** Viral genomes by viral family. A total of 2,171 viral genomes were identified from metavirome and metagenome assemblies. The table below shows their annotations by source.

	Metavirome only	Metagenome only	Both
*Caudovirales* families			
*Myoviridae*	148	65	48
*Podoviridae*	37	5	12
*Siphoviridae*	910	214	242
Unassigned *Caudovirales*	138	45	23
Other viral families *Anelloviridae*	6	0	0
*Circoviridae*	86	0	0
*Cruciviridae*	5	0	0
*Geminiviridae*	2	0	0
*Genomoviridae*	1	0	0
*Inoviridae*	1	1	0
*Microviridae*	50	0	3
*Mimiviridae*	0	1	0
*Nanoviridae*	1	0	0
*Phycodnaviridae*	3	1	0
Unassigned viruses	109	9	2
TOTAL	1499	330	342

**Figure 1.**
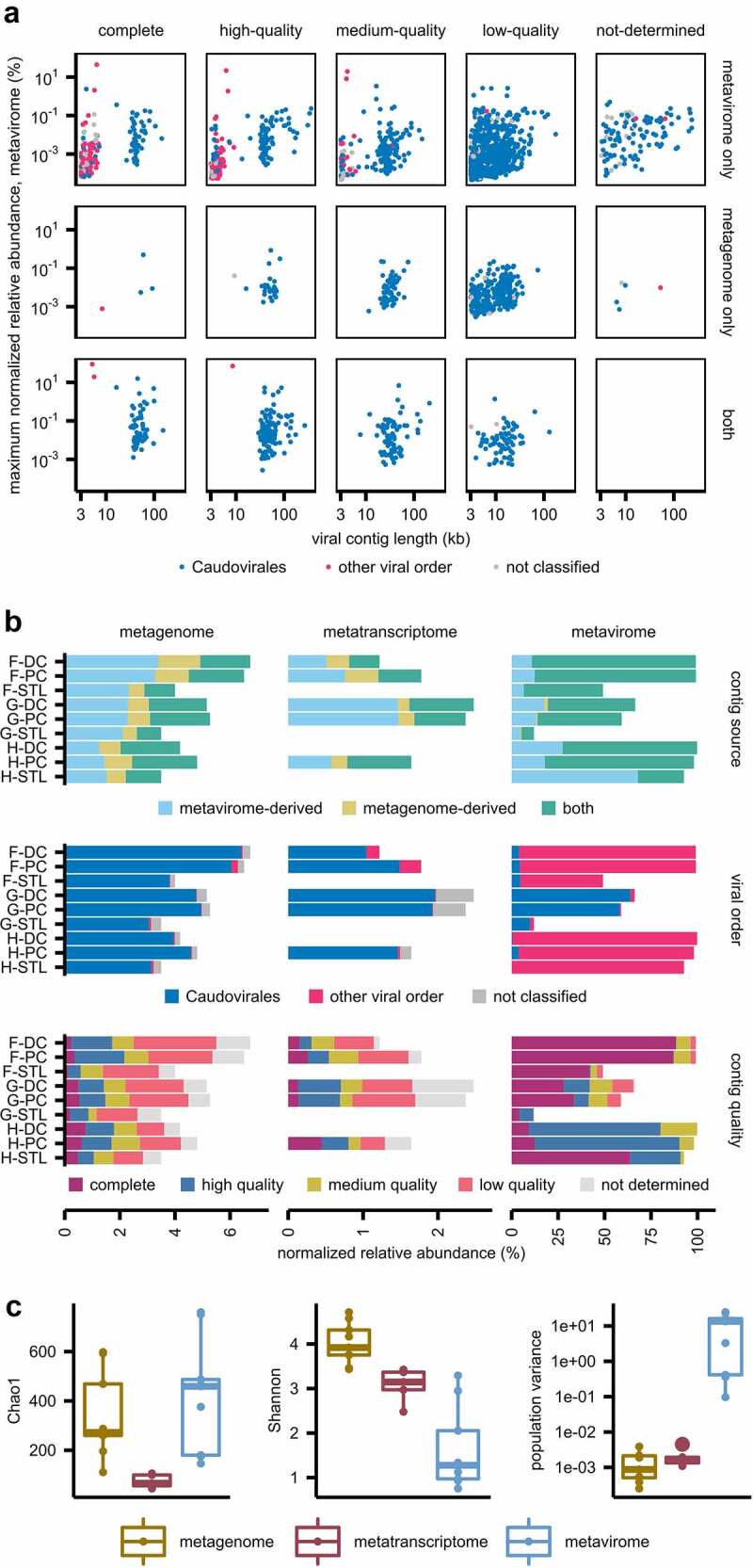
Viral contig annotation and alpha-diversity across metagenomic, metatranscriptome, and metaviromic datasets. (a) 2,171 viral contigs (VCs) were identified across metaviromic and whole metagenomic assemblies and are plotted by: dataset of origin, viral contig quality, maximum normalized relative abundance (NRA) in the metavirome in any given sample, contig length, and viral order. (b) NRA of viral contigs across multiomic datasets for each participant’s samples, annotated by source, taxonomy, and quality. (c) Boxplots of Chao1, Shannon diversity and population variance by multiomic dataset. *p-value < 0.05; **p < .001. DC: distal colon, PC: proximal colon, STL: stool.

VCs are plotted in Figure 1a by viral order, quality, contig length, maximum observed NRA in the metavirome, and dataset identified (i.e. metavirome, metagenome, or both). VCs identified in both the metavirome and metagenome were more likely to be longer (*p* = 6.52e-11), abundant (*p* < 2e-16), and less likely to be low-quality (*p* < 2e-16). Thus, co-presence in both datasets could be used as a marker for high-quality VCs. *Caudovirales* contigs were significantly longer than those belonging to other viral orders (mean length of 24.8 kb vs. 5.3 kb, *p* < 2e-16 by Wilcoxon rank sum test) which reflects the smaller genome sizes of *Microviridae* and *Circoviridae* species; this difference was even greater when only including complete and high-quality VCs (48.9 kb vs. 4.5 kb, *p* < 2e-16). Lower-quality viral contigs were also significantly more likely to be shorter. As viral databases continue to grow, we recommend including the type of source dataset (i.e. VLP-enriched vs. whole metagenome) as key annotations alongside taxonomy and quality to help evaluate the influence of sequencing methodologies on human virome profiles.

### Viral contigs across metagenomic, metaviromic, and metatranscriptomic datasets

Viral contigs represented 75.1% of the metavirome (by NRA), 4.85% of the metagenome, and 1.90% of the metatranscriptome (Figure 1b). The 2.5-fold decrease from the metagenome to the metatranscriptome suggests that the virome may be less transcriptionally active than the bacteriome, consistent with hypotheses of a predominantly temperate phage-host dynamic in the intestinal microbiome.^3,32^

While only 15.8% (342/2,171) of VCs were identified from both the metagenome and metavirome, these VCs were highly abundant, representing an average of 36.4%, 35.6%, and 73.1% of the observed viral community in the metagenomic, metatranscriptomic, and metaviromic datasets by NRA, respectively. The metagenome also contained metavirome-derived VCs that were not identified from the whole metagenomic assemblies. This finding may be due to improved assembly of viral contigs in VLP-enriched samples (with greater sequencing depth per VC), and due to different thresholds employed by Cenote-Taker2 when identifying viral assemblies in whole metagenomes as compared to VLP-enriched metaviromes, the former being more stringent to reduce false positives. It remains pertinent to consider the impact of bioinformatic tools and approaches in the identification of viral sequences,^33,34^ where multiomic approaches could be utilized to corroborate and contextualize viral contig identification.

Viral contigs of the order *Caudovirales* represented the most abundant viruses of the metagenome and metatranscriptome while other viral taxa, primarily *Microviridae*, were enriched in the metavirome. Random displacement amplification, a common step employed in virome sequencing efforts, including this study, has been reported to introduce positive bias for small, circular ssDNA viruses such as *Microviridae*.^35,36^ However, the patterns of ssDNA virus enrichment in our dataset appear to be participant-specific: all virome samples from participant G were *Caudovirales*-dominant while all samples from participant F and H were *Microviridae*-dominant. We have also previously shown that this virome sequencing protocol does not interfere with relative quantification of a spike-in phage in mucosal-luminal interface samples.^11^ Thus, the expansion of *Microviridae* in participants F and H is likely suggestive of a true relative enrichment of these viruses in comparison to participant G.

We also observed that metaviromic reads were more likely to map to complete or high-quality viral contigs compared to metagenomic or metatranscriptomic reads. This supports the use of CheckV annotations to infer viral genome completeness. Lower quality genomes may include incomplete prophages, chimeric assemblies, or low abundance phages that lacked sufficient coverage for full genome assembly. We thus chose to use these CheckV quality thresholds when examining viral gene transcription and prophage induction as described in subsequent sections.

Next, we assessed the virome’s alpha-diversity as observed across multiomic datasets; read counts to VCs were rarefied for these calculations. The Chao1 index, which evaluates species richness, was significantly lower in the metatranscriptome (*p* = .0015, Wilcoxon rank sum test), suggesting that only a subset of viruses are transcriptionally active (Figure 1c). The similar Chao1 indices between metagenome and metavirome suggest that both methods capture a similar number of VCs per viral sequencing read, as has been previously reported.^6^ However, a metagenome sample would require a greater sequencing depth of 1–2 orders of magnitude to be comparable to metaviromic sequencing. The Shannon index and population variance show that the viral community as seen in the metavirome is less evenly distributed than in the metagenome (p = .00012) or metatranscriptome (p = .0015); the Shannon index also shows that the metatranscriptome population is less evenly distributed than the metagenome (p = .0015). The reduced evenness in these datasets may be due to prophage activation, virulent phage replication, or virulence factors, that enable select viruses to become highly abundant in the metatranscriptome and metavirome, including the *Microviridae*-dominance seen in select virome samples.

In all, we demonstrate that each sequencing method provides a distinct snapshot of the human gut virome. With the majority of virome studies utilizing either metagenomic or metaviromic approaches, it is important to recognize each technology’s biases on the observed taxonomy, quality, and community dynamics of identified viral populations, and to utilize multiomic approaches where possible to capture the true complexity of these viral communities.

### The virome at the colonic mucosal-luminal interface is distinct from the stool virome

**Figure 2.**
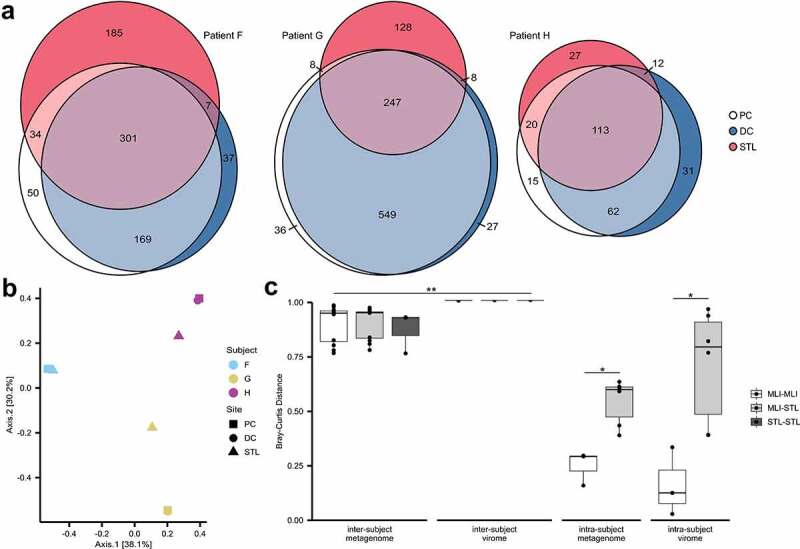
Beta diversity of colonic and stool metavirome communities. (a) Euler diagrams showing the number of shared viral contigs (with a minimum of 75% of breadth of coverage) between sampling sites. (b) Principal coordinate analysis showing Bray-Curtis dissimilarities of metavirome communities. (c) Bray-Curtis dissimilarities of viral and non-viral metagenomic communities between MLI and STL samples, plotted by intra-individual and inter-individual comparisons. *p-value < 0.05. **all inter-individual metagenome communities had a significantly lower Bray-Curtis distance than inter-individual metavirome communities (*p* < .01). DC: distal colon, PC: proximal colon, STL: stool, MLI: mucosal luminal interface.

Several studies have investigated differences between the luminal and mucosal bacteriome along the length of the gastrointestinal tract.^18,37,38^ Different ecological pressures would therefore be expected to impact the intestinal viral community along both radial and longitudinal dimensions, though there have been very few efforts for multi-site, or spatial, characterization of the human virome. Here, we provide a direct comparison between the proximal colon MLI, distal colon MLI, and stool (Figure 2a). Among the three participants, 24.6% to 40.4% of VCs were identified at all three sites, representing a shared viral community throughout an individual’s colonic mucosa and lumen. With 32.7 to 61.0% of VCs unique to MLI samples and 9.6 to 23.6% of VCs unique to stool, multiple site sampling substantially expands an individual’s known intestinal virome. Bray-Curtis dissimilarities between these samples show the MLI samples of participants G and H clustering apart from the stool, while participant F, the only patient to have pancolitis, has all three samples clustering together (Figure 2b). Multi-site sampling allows for an increased spatial resolution to understand viral-host interactions, especially in conditions like inflammatory bowel disease.

Consistent with other studies demonstrating personalized microbiomes and viromes, inter-participant metagenome and metavirome diversity was significantly greater than between intra-participant sites (Figure 2c). Bray-Curtis dissimilarities of the non-viral metagenomic community demonstrated reduced distances as compared to the virome (p ≤ .009) indicating that the virome is more individualized than the metagenome. Within the same patient, Bray-Curtis distances between the proximal and distal colon samples were smaller than those between the colonic mucosa and stool (p = .037), suggesting that despite heterogeneity in local inflammation (such as a non-inflamed proximal colon and an inflamed distal colon), the colonic mucosa provides a common niche that is distinct from the luminal, stool virome. We hypothesize that bacteriophage adherence to mucin, facilitated by interactions between mucin glycoproteins and phage capsid proteins, supports the development and persistence of a mucosa-associated virome.^39,40^ Our data also demonstrated a trend for greater species richness (i.e. Chao1 index) at both MLI sites in comparison to stool in each participant. Larger studies are required to further investigate these observations and to assess whether these findings extend beyond pediatric subjects with inflammatory bowel disease. Characterizing the mucosa-associated virome will expand the known human virome while enabling the study of microbial communities that are more likely to interact with human health and intestinal diseases.

### Viral contig transcription and prophage activation in the metatranscriptome

A total of 59,217 open reading frames (ORFs) were predicted across the set of 2,171 VCs, which were further clustered into 28,112 viral genes and annotated using a combination of five protein family databases: two viral orthologous group databases VOGDB and pVOG,^41,42^ and three general-purpose databases PFAM, KEGG, and TIGRFAM.^43–45^ This approach annotated 9,646 (34.3%) viral genes, with PFAM annotating the most viral genes (Supplementary Figure 3A). However, VOG and pVOG annotations were more effective at annotating abundant viral genes detected in the metavirome, supporting the use of more tailored viral databases (Supplementary Figure 3B). We thus assigned viral functions in the order of the databases listed above, with VOGDB being the primary database used in this study.

**Figure 3.**
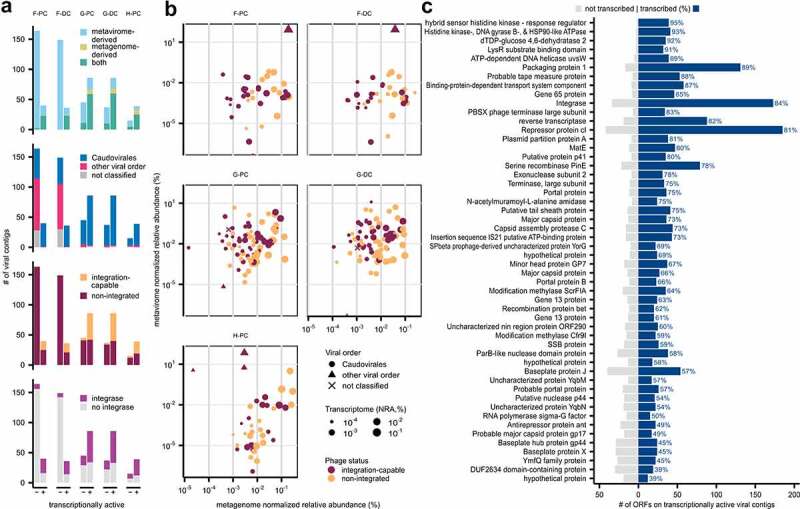
Transcriptionally active viromes of the colonic mucosal-luminal interface. (a) Bar plot showing all complete and high-quality VCs by metatranscriptome activity (minimum abundance of 0.0001%), further annotated by viral contig source, taxonomy, phage integration, and integrase presence. (b) Scatter plots highlighting transcriptionally active VCs by their NRA in the metagenome, metavirome, and metatranscriptome. (c) Bar plot showing annotated ORFs of transcriptionally active VCs present in ≥30 instances (i.e. counted for every VC and every sample). Transcribed ORFs (i.e. present in that sample’s transcriptome) are counted on the right side of the plot.

To assess viral gene transcription, we focused on the subset of complete and high-quality VCs (580/2,171). We classified these VCs as “present” in a sample’s metagenome and/or metavirome if detected with a minimum breadth of coverage of 75%, or as “transcriptionally active” (TA) if they had a minimum NRA ≥0.0001% in the sample’s metatranscriptome. These TA-VCs, highlighted in Figure 3a, were more likely (p = .016) to be present in both metagenome and metavirome datasets (64.8%) than non-transcriptionally active VCs (17.2%). We hypothesize that TA-VCs which are present in all three multi-omic datasets reflect the host-dependent production process of free viral particles. TA-VCs were less likely to be derived solely from the metavirome, which could represent viral particles without available hosts including transient, luminal VLPs.

Most TA-VCs were *Caudovirales* (96.0%, compared to 64.2% of non-transcriptionally active VCs, p = .018) with only a small minority of *Microviridae* detected in the metatranscriptome. This observation could suggest that the non-*Caudovirales* viruses are more likely to be transient while also reflecting the sequencing bias of small circular genomes in commonly used metavirome sequencing efforts, though future multiomic studies with greater sample sizes are required to further explore these findings. TA-VCs were also more likely to be observed with flanking host regions (47.0% vs. 7.5%, p = .0095) and contain an integrase (62.4% vs. 27.8%, p = .016); however, the latter observation was no longer significant when excluding non-*Caudovirales* VCs, which rarely contain an integrase.

Using metagenomic, metaviromic, and metatranscriptomic sequencing data, we plotted virome transcription profiles of each sample (Figure 3b). The NRA of viral contigs in the metatranscriptome was most correlated with their NRA in the metagenome (Pearson correlation = 0.395, p = 3.9e-12). The NRA of viral contigs in the metavirome was also positively correlated with their NRA in the metatranscriptome (Pearson correlation = 0.307, p = 1.1e-7), suggesting that observed increases in viral transcription may translate to increased VLP production. The NRA of VCs in the metavirome was also correlated with their NRA in the metagenome (Pearson correlation = 0.136, p = .021); the relatively weaker correlation between the metagenome and metavirome once again demonstrates the differences between whole metagenome and VLP-enriched sequencing approaches. The similarity between colonic sites is further visualized in Supplementary Figure 3C, reflecting the inter-participant variability and intra-participant stability of the virome as described in the previous section. More samples are required to investigate differences along the gastrointestinal tract and to compare viromes between subjects with and without IBD, while longitudinal studies will provide further insight into temporal variations in the virome’s transcriptomic activity.

Within the subset of complete and high-quality TA-VCs, we also examined the metatranscriptome dataset at the feature level. The most frequently occurring genes are shown in Figure 3c, led by repressor protein cI (n = 227), integrase (207), and packaging protein 1 (148). Gene transcription varied from 39% to 95%, with several genome processing genes (DNA helicase uvsW, 89%; integrase, 84%; reverse transcriptase 82%) showing higher transcription than structural proteins (major capsid proteins, 49–73%; baseplate proteins, 45–57%). While inferring population-level trends remains difficult given significant viral heterogeneity, we hypothesize that this pattern reflects the increased presence of early-stage bacteriophage genes, many of which are supportive of phage lysogeny, as compared to late-stage genes. The greater transcription of repressor protein cI (81%) compared to the anti-repressor protein ant (49%) may also provide further evidence of an overall temperate viral community.

### Two abundant crAss-like phages identified at the MLI

Two circular *Caudovirales* VCs, V014264 (97.91 kb) and V016904 (99.54 kb), had high similarity to existing delta crAss-like phages: ERR844016_ms_1 and ERR844030_ms, respectively.^7^ The comparative phage genome tool VIRIDIC showed that each VC had 95.3% and 89.9% identity with their aligned crAss-like phage, 15.3% with each other, and less than 2% with the first isolated crAssphage.^46,47^ These were the only two VCs in our dataset that contained all three markers (portal, TerL, major capsid protein) utilized in a recent profiling of crAss-like phages.^48^ While crAss-like phages have been observed in up to 90% of metavirome samples,^47^ the rarity of these phages in our IBD-only dataset could reflect a recent study which showed a depletion of crAss-like viruses in patients with inflammatory bowel disease.^48^ The prevalence and diversity of crAss-like phages also appear to increase with age;^49^ here, we profiled adolescents, a rarely studied demographic within virome studies. A larger dataset is required to better capture the prevalence of crAss-like phages in the human colonic mucosa, including in the context of human disease.

Both crAss-like phages were highly abundant in the proximal and distal colon metaviromes of participant G (Supplementary Figure 4), with V014264 being the third most abundant VC (NRA of 4.82%) in the proximal colon. We did not detect any single-nucleotide polymorphisms in either VC between the two sites.^50^ These crAss-like phages were detected in the metagenome and were among the subset of complete and high-quality, transcriptionally active VCs (highlighted in Supplementary Figure 3C). Both crAss-like phages were also present at very low abundance (< 2 × 10^–4^ %) in the distal colon metavirome of patient H.

While abundant in MLI metaviromes, both crAss-like phages were nearly undetectable in the stool metavirome of participant G (2.6 x 10^−6^% for V014264; 1.4 × 10^–7^ % for V016904). These reads were below our filtering thresholds and exceeded a million-fold decrease from the MLI samples. At a more conventional sequencing depth, these crAss-like phages would be effectively undetectable by stool sampling. Both VCs were among the 61.0% of MLI-specific VCs detected in participant G (Figure 2a), demonstrating the importance of distinct viral niches across our complex biogeography.

**Figure 4.**
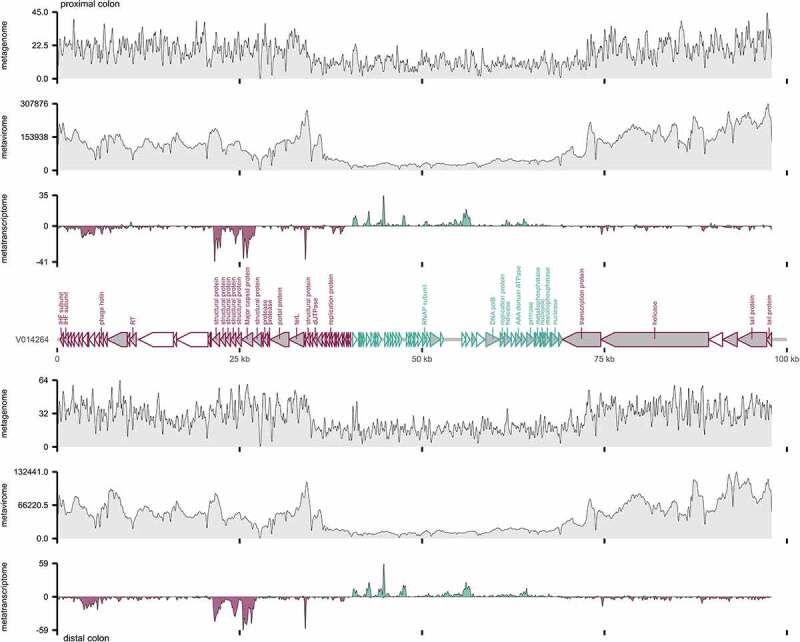
Multiomic sequencing map of V014624, a crAss-like phage identified in the colonic mucosal-luminal interface. Open reading frames (ORFs) were predicted over the 97.9 kb genome and colored by forward (green) or reverse (purple) orientation. Annotated ORFs are shaded in gray and labeled (excluding ‘uncharacterized’ or ‘hypothetical’ proteins). Metagenome, metavirome, and metatranscriptome sequencing depths at the proximal colon (top) and distal colon (below) are plotted using a 151 bp sliding window; metatranscriptome reads are also mapped by strand. Another crAss-like phage, V016904, is shown in Supplementary Figure 5.

Multiomic coverage maps of V014264 and V016904 are shown in Figure 4 and Supplementary Figure 5. Functional annotation of the crAss-like phages was performed as described above using Prokka and several viral and general-purpose databases, yet this only annotated 11.9% and 2.4% of ORFs on V014264 and V016904, respectively (Supplementary Figure 6A). Utilizing a curated set of crAss-like phage protein families described in Yutin, *et al*.,^51^ we were able to increase the proportion of annotated reads to 39.6% and 32.1%, albeit including limited annotations such as “uncharacterized protein of delta crassfamily delta group phages.” Metatranscriptomic sequencing reads, when mapped to forward and reverse strands, corroborated the orientation of the predicted crAss-like phage genes and enabled gene-level analysis (Figure 4). For V014624, 55.4% of the contig was transcribed in the proximal colon while 70.7% was transcribed in the distal colon (i.e. with a minimum sequencing depth of 1). Between the two sites, metavirome map depths were highly correlated (Spearman correlation = 0.983), compared to 0.585 in the metagenome and 0.504 in the metatranscriptome, suggesting that the transcription profiles of these crAss-like phages were similar in both the non-inflamed proximal colon and inflamed distal colon.

Considering all transcribed genes, V016904 had a higher mean gene transcription ratio (transcriptome/metagenome) than V014264 (0.62 vs. 0.23, p = 4.3e-15), while VC16904 was significantly more transcribed in the proximal colon than the distal colon (0.80 vs. 0.44, p = .00078) (Supplementary Figure 6B). The most transcribed gene clusters are highlighted in Supplementary Figure 6C, with a GIY-YIG endonuclease and major capsid protein among the highest transcribed crAss-like phage genes in this dataset. We also note the presence of highly transcribed regions without annotated ORFs (i.e. a 500 bp region at ~44.5 kb) on V014264, that could be involved in some form of regulatory role to support the stable relationship between crAss-like phages and their hosts.^17,52^ Despite significant improvement with targeted databases, virome studies continue to be limited by poor viral genome annotation. As these databases continue to grow with our understanding of bacteriophage function, metatranscriptomics have great potential in capturing the *in vivo* functional capabilities of our human viromes.

### Host-phage prediction and transcription

**Figure 5.**
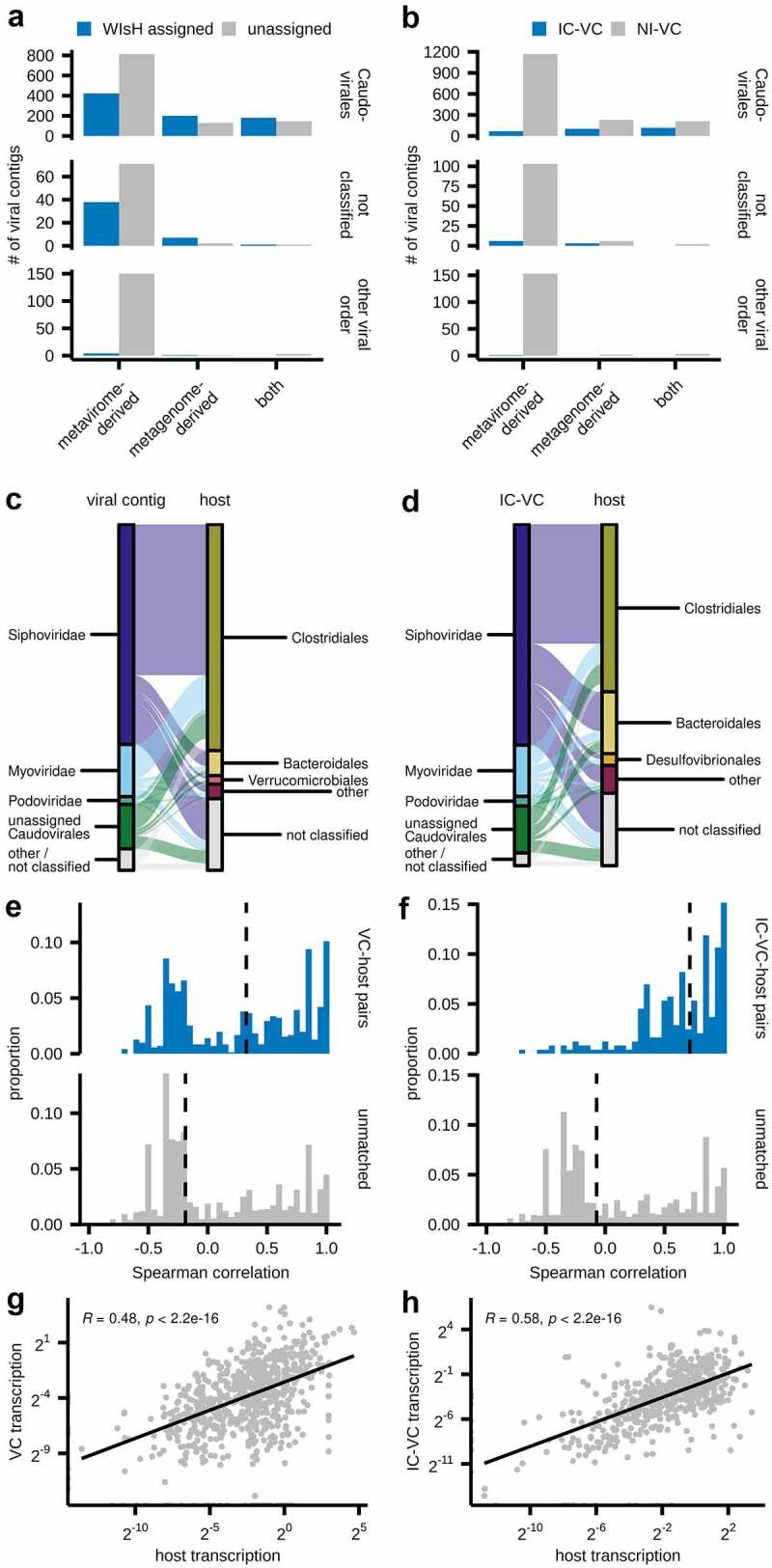
Phage-host prediction inferred from metavirome and whole metagenome assemblies. Phage host relationships as calculated using WiSH (A,C,E,G) or inferred by the presence of non-viral flanking regions of VCs (B,D,F,H). (a-b) Bar plots of all VCs by source, viral order, and their host assignment status. (c-d) VC-host or IC-VC–host combinations by viral and bacterial order. (e-f) Spearman correlations of the NRAs of matched VC-host or IC-VC host pairs, as compared to unmatched pairs. The dashed line indicates the median. (g-h) Scatter plots of transcription (i.e. metatranscriptome/metagenome NRA), with the line and formula representing the linear model and Pearson correlation between VC and host transcription.

While there are multiple methods to predict bacterial host-phage relationships, we utilized two approaches that leveraged our paired metavirome and whole metagenomic sequencing (Figure 5). First, we used a probabilistic modeling approach (WiSH) to assign VCs to their most likely host within our set of whole metagenome-assembled, non-viral contigs. After applying a false-discovery rate adjusted *p*-value threshold of 10^−5^, 855 (39.4%) VCs were assigned a host contig. Nearly all VCs assigned a host were *Caudovirales* (Figure 5a). Few *Circoviridae* (2/86) and *Microviridae* (1/53) were assigned hosts. While ssDNA viruses have shorter genomes (*Microviridae* and *Circoviridae* have a mean length of 5.2 kb and 4 kb in our dataset, respectively) and there is a correlation between VC length and host assignment, short genomes do not preclude host assignment: 25.8% of all our host-assigned VCs are less than 6 kb. Moreover, ssDNA viruses have been reported to be less adaptive to their host genomes and have a higher mutation rate,^4,53,54^ which could limit our ability to assign their hosts. Additionally, ssDNA virus enrichment due to random displacement amplification could result in a lack of host-assignment if its corresponding bacterial genome was below our sequencing or contig assembly detection threshold. Finally, three *Phycodnaviridae* VCs were assigned a bacterial host, which could represent a false prediction or an incorrect annotation, as *Phycodnaviridae* are known to infect algae rather than bacteria.

VCs identified in the metagenome were also more likely to be assigned a bacterial host than those identified solely in the metavirome (Figure 5a). We hypothesize that whole metagenome sequencing enables the identification of prophages and intracellular phage particles within their host bacteria that may be missed by metavirome sequencing, while VLP-enriched methods may capture transient VLPs that are unrelated to the resident bacteriome. Figure 5c shows the breakdown of host-viral pairs by the predicted VC family and bacterial host contig order, with most VCs assigned to bacteria in the order Clostridiales.

Our second approach for studying virome-bacteriome interactions involved our subset of IC-VCs with flanking host regions. These 296 IC-VCs were associated with 309 unique host contigs, with 320 unique VC-host pairs (23 VCs were present in 2 or 3 host contigs; 11 host contigs contained 2 VCs). Like WiSH-assigned VCs, IC-VCs were nearly all *Caudovirales* and more likely to be derived from the metagenome or both the metagenome and metavirome (Figure 5b). IC-VC hosts were also most commonly of the bacterial order Clostridiales, though compared to WIsH assignments, Bacteroidales hosts were proportionally increased from 7.3% to 18.1% (Figure 5d).

Spearman correlations were calculated between the NRA of each VC in the metavirome and the NRA of its predicted host in the metagenome across the nine paired datasets (Figure 5e, 5f). The NRA of both WiSH-assigned VC-host pairs and IC-VC-host pairs were significantly (p < 2.2e-16) more likely to be positively correlated in comparison to the NRAs of unmatched pairs, with median Spearman correlations of 0.325 and 0.713, respectively. Viral contig transcriptional enrichment (i.e. NRA of contig in the metatranscriptome/metagenome) also positively correlated with host contig transcriptional enrichment (Figure 5g and 5h). These results are indicative of phage-host coexistence in the mammalian gut mucosa, and suggests that the transkingdom equilibrium as demonstrated between ΦcrAss001 and its host *B. intestinalis* may also be reflected at the community level.^17^ The gut mucosa is thought to enable a heterogenous distribution of phages and their bacterial hosts that supports their co-existance.^55^ In our small IBD cohort, this apparent co-existence is maintained despite the presence of local host inflammation, though the impact of inflammation on virome-bacteriome interactions at the MLI requires further study.

### The presence of an integrase is a weak predictor for observed viral lysogeny

Phage integrases are viral enzymes that facilitate site-specific recombination, allowing for the integration of a viral genome into its host. Many model bacteriophages, such as *Escherichia virus Lambda*, require an integrase for lysogeny.^56,57^ Additionally, integrases tend to be relatively prevalent in virome datasets including this study, where integrases were the most common viral gene annotation overall (second-most common in complete and high-quality VCs). Integrases have therefore been commonly employed as prophage markers and as indicators of a temperate lifestyle.^4,53,58,59^

Among our 2,171 VCs, we identified 603 integrase genes (591 VOG00035 “Integrase”, 10 pVOG0275 “integrase”, 1 VOG11667 “DDE-type integrase/transposase/recombinase”, and 1 PF13495 “Phage integrase, N-terminal SAM-like domain”) on 497 VCs. All identified PFAM-annotated integrases (PF00589), commonly used for integrase identification, were encompassed within the VOG00035 integrases. These integrases were present on 25.8% of *Caudovirales* VCs (489/1892) and absent in all other viral orders. Longer and higher-quality VCs were more likely to contain an integrase gene. We thus focused on the subset of complete and high-quality *Caudovirales* VCs, of which 54.9% (219/399) contained an integrase.

**Figure 6.**
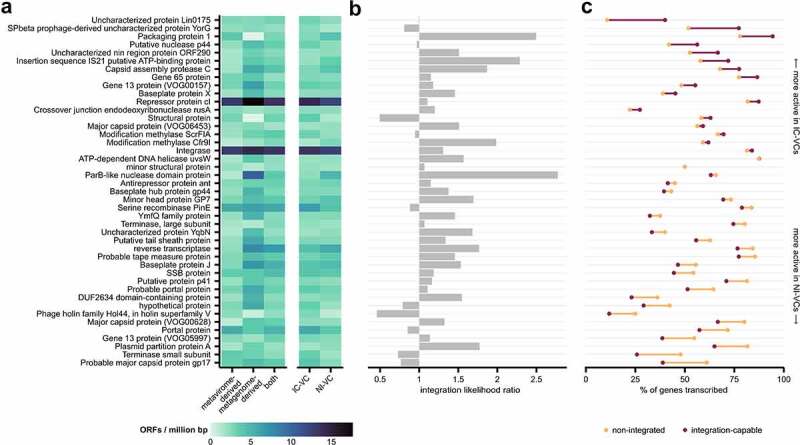
Presence and transcription of common viral ORFs including lysogenic proteins on *Caudovirales* VCs. (a) The frequency of common viral genes (≥10%) present on complete and high-quality *Caudovirales* VCs based on contig source and between IC-VCs and NI-VCs. ORF counts were normalized by contig length. (b) Integration likelihood ratios of based on presence of each gene family. (c) Proportion of genes transcribed in IC-VCs and NI-VCs, sorted by difference.

IC-VCs were more likely to contain an integrase (69.6%, 71/102) than non-integrated VCs (49.8%, 148/297); this finding was independent of contig length. While this result is expected, the majority of integrase-containing VCs were only detected as free phages, and thus suggests that many NI-VCs may be temperate phages observed only in their lytic cycle. We therefore investigated the transcription of all common gene families including integrases (present in 10% or more of high-quality or complete *Caudovirales* VCs) for their association with observed phage integration (Figure 6).

Repressor protein cI (VOG06177) and integrase (VOG00035), both known to be involved in phage lysogeny, were by far the most common gene annotations. However, both were similarly present in IC-VCs and NI-VCs when normalized by contig length and had modest integration likelihood ratios of 1.11 and 1.31, respectively (Figure 6b). In comparison, the highest observed integration likelihood ratios were for ParB-like nuclease domain protein (2.77), packaging protein 1 (2.50), and insertion sequence IS21 putative ATP-binding protein (2.29). We thus suggest that the presence of an integrase gene alone is a weak predictor of a VC lysogeny, as it is neither a sensitive nor specific indicator of phage integration. Existing databases may limit our capacity to annotate the diverse array of enzymes that enable phage recombination, which may include various integrases, transposases, and recombinases.^57^ Our own annotation sensitivity would have been further reduced if we had relied only on the commonly used PFAM integrase family PF00389, which missed 15.9% of integrase genes identified with VOG00035. Successful identification of flanking host regions is also dependent on accurate metagenomic contig assembly and subsequent annotation, and thus limits the utility of using these regions as a gold standard for lysogeny.

Given that the presence of integrase and repressor protein merely infers lysogenic potential but may not clearly differentiate phages in their lytic or lysogenic cycle, we hypothesized that transcription would be a better indicator of phage lysogeny. We thus examined the transcription of these common viral genes (Figure 6c). Integrases and repressor proteins were both highly transcribed, which supports the overall lysogenic nature of the intestinal virome community. Both integrases and repressor protein cI were slightly more transcribed in VCs with flanking host regions (Figure 6c), increasing from 81.4 to 83.9% and 81.8% to 87.3%, respectively, which supports the hypothesis that some temperate prophages are being detected during a lytic cycle. Packaging protein 1, putative nuclease p44, and two uncharacterized proteins were most likely to be transcribed in IC-VCs (+14.3–29.3%), while several terminases and major capsid protein genes were more transcribed in NI-VCs, consistent with these phages being in their lytic phase. Improved viral annotation and further phage studies will assist with the prediction of phage lifestyle, especially since phage-host relationships outside of model organisms remain poorly understood. Notably, crAss-like phages display features of temperate phages including high-level long-term persistence without plaque formation in its host, yet both ΦcrAss001 and ΦcrAss002 do not contain lysogeny modules.^17,60^ We also did not detect integrases within crAss-like phages V014264 and V016904, though integrases were noted within a larger subset of crAss-like phages.^51^ Ultimately, phage integration is likely among other lysogenic mechanisms including episomes, pseudolysogeny, phage carrier states, and other uncharacterized means, that are able to sustain stable viral populations.^61^

We thus demonstrate the potential for integrating metagenomics, metaviromics, and metatranscriptomics to evaluate viral lysogeny in a cohort of pediatric, IBD subjects. Through metatranscriptomics, we revealed that lysogenic proteins are highly transcribed at the community level, while the presence and transcription of these proteins could be used to predict phage lysogeny at the genome level. We recommend against inferring phage lifestyle based on the presence of an integrase alone, and instead include additional factors such as integrase transcription, alternative integration-associated genes, and the presence of flanking host DNA. To help overcome limitations in viral gene databases, machine-learning classification tools and alignment-free *K*-mer frequency models are also being developed to assist in phage lifestyle prediction.^62,63^

### Metatranscriptomic sequencing does not reveal a significant population of RNA phages

We also searched our metatranscriptomic data for RNA phages, which may be missed in DNA-based approaches for metagenomics and metaviromics. Predicted ORFs on metatransciptomic assemblies were searched against two protein profile databases: a set of RNA-dependent RNA polymerases (RdRps) and capsid genes curated for Cenote-Taker2, and a set of ssRNA marker genes that were used in a recent identification of 15,611 non-redundant environmental ssRNA phages.^1,14^ Both MLI samples in participant F and G contained an RdRp originally identified in a dsRNA human picobirnavirus.^64^ There were no other RdRps or ssRNA phage markers detected in our metatranscriptomic dataset. Thus, like prior studies, we report a scarcity of RNA phages in the human virome, though these observations remain hampered by limited RNA phage databases and a small, IBD cohort with no control subjects.^65^ Additionally, our metatranscriptome protocol, which like our metagenome protocol involves pelleting bacterial cells prior to DNA/RNA extraction, may exclude viral VLPs that remain in the supernatant.^11^ Alternative methods to capture RNA-based VLPs would be required to assess their true contribution to the mucosa-associated virome.

### The impact of sequencing depth on virus discovery

**Figure 7.**
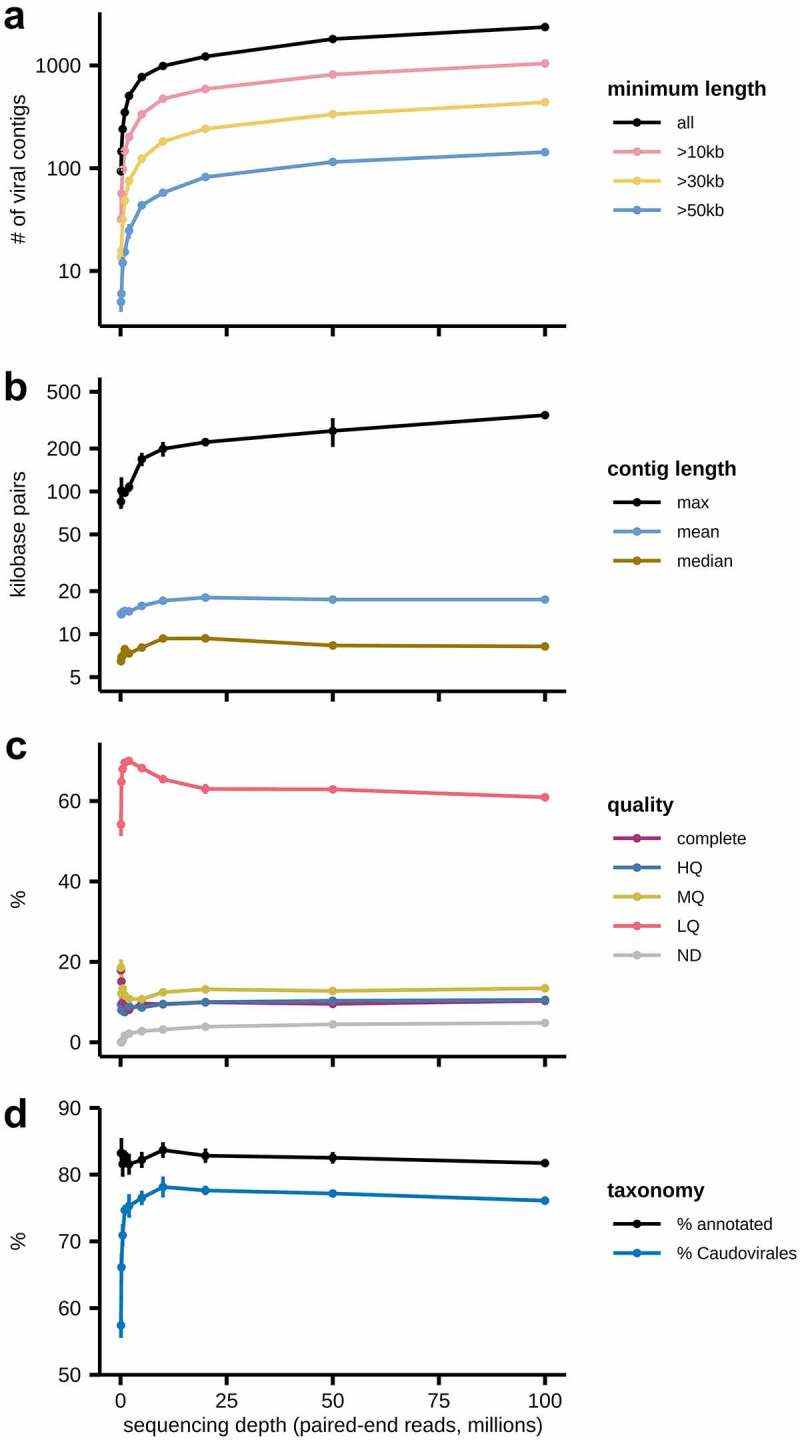
Effects of sequencing depth on viral contig identification. The impact of sequencing depth on (a) number of VCs identified, (b) VC length, (c) contig quality, and (d) annotation. Error bars show standard deviation. HQ: high-quality; MD: medium-quality; LQ: low-quality; ND: not-determined.

Virome sequencing has substantially improved over the past decade, from read counts in the 10,000s to over ten million paired-end (PE) reads. To investigate the impact of sequencing depth on virome discovery, we subsampled our metaviromic sequencing reads at increments spanning 10 k and 100 million PE reads to examine how sequencing depth affects VC identification (Figure 7). Empirically, the yield for new contigs per million bases dropped from 38.6 VCs at 1 million reads to 9.4 VCs at 10 million reads to 2.3 VCs at 100 million reads (Figure 7a). Mean contig length peaked at 20 million PE reads (18.1 kb) but was overall stable between 17.1 and 18.1 kb above 10 million reads (Figure 7b). Maximum contig length appeared to stabilize at around 10 million reads but did continue to increase as the sequencing depth reached 100 million reads. At all sequencing depths above 100,000 reads, VC quality remained remarkedly stable, with 8.1 to 10.3% of all VCs annotated as “complete” by CheckV (Figure 7c). Taxonomic annotations of VCs also remained relatively stable at sequencing depths greater than 2 million PE reads (Figure 7d). This study is one of the deepest sequenced datasets to date and allowed us to explore the impact of sequencing depth on virome identification. We show that after about 10–20 million PE reads, there were no further gains in mean contig length, viral contig quality, or contig annotation. Thus, given the existing bioinformatic tools currently available, it is unlikely that extensive sequencing depth will ultimately result in a set of “complete genomes”. As such, it may be more practical for virome researchers to design experiments to maximize the information obtained from a particular sample, as the “holy grail” of assembling all phages that may be present within a complex virome community is likely unfeasible. We also found that increasing the read depth leads to a decrease in the percent contigs which could have an annotation assigned. While shorter DNA lengths may contribute to this, it is equally likely that rarer, low-abundant viruses are less likely to be characterized and are thus more difficult to classify.

## Conclusions

In summary, we present an in-depth, multiomic spatial analysis of the human virome in a small cohort of pediatric patients with ulcerative colitis. We demonstrated that the mucosal-luminal interface virome was distinct from stool and were able to isolate two highly abundant, mucosa-associated crAss-like phages, highlighting the importance of studying the virome across our complex host biogeography. We then developed practical approaches to leverage metagenomics, metaviromics, and metatranscriptomics to study the virome at the community and contig level. Because each technique yields a different viral subset, multiomic approaches help to corroborate and contextualize observations while revealing biases that are present in metagenome or metavirome only studies. Viral contigs present across all three datasets were more likely to represent higher-quality *Caudovirales* genomes. Many of our observations were consistent with an overall temperate virome, including reduced virome representation in the metatranscriptome, co-existence of predicted phage-host pairs, and high transcription of lysogeny-associated genes. We also assessed integrases and the presence of flanking host DNA regions and show that metatranscriptomic analysis improves our ability to infer phage lysogeny.

While these findings were described in a pediatric IBD cohort and may not extend to the general population, we describe techniques that advance our ability to characterize the human virome. Providing further biogeographic resolution of our virome and employing multiomic approaches are two key frontiers in understanding how the virome interacts with the bacteriome in the context of human health and disease.

## Patients and Methods

### Resource Availability

#### Lead contact

Further information and requests for resources and reagents should be directed to and will be fulfilled by the lead contact, Alain Stintzi astintzi@uottawa.ca.

#### Materials availability

This study did not generate new unique reagents.

#### Date availability

The datasets generated during this study are available at NCBI PRJNA818303.

## Experimental Model and Participant Details

Sample collection from pediatric patients was approved by the Research Ethics Board of the Children’s Hospital of Eastern Ontario (CHEO) in Ottawa, Canada with informed consent/assent obtained from parents and/or participants. Mucosal luminal interface samples from three treatment-naïve patients were obtained during diagnostic endoscopy following a standard colonoscopy preparation protocol.^66^ Demographic and clinical information is shown in Table 1. Stool samples were collected either before or after endoscopy and stored at −80°C.

## Method Details

### Sample Collection

The collection of mucosal-luminal interface (MLI) aspirates has been described previously.^18^ In brief, sterile water was used to wash the bowel wall at the proximal and distal colon during colonoscopy to remove the loosely adherent mucous layer. The wash was then aspirated into a sterile container and stored at −80°C. Aliquots of 10 ml were used for virus-like particle purification and viral DNA extraction; 20 ml were used for RNA extraction, and 2 ml was used for whole metagenomic DNA extraction.

### Virus-like Particle Purification and Nucleic Acid Extraction

We have previously described a protocol to purify virus-like particles from mucosal aspirates.^11^ In summary, 10 ml aliquots of mucosal aspirates or 0.5 g of stool homogenized in 10 ml saline-magnesium buffer were subjected to centrifugation and sequential filtration at 5.0 and 0.45 µm filters (Sigma-Aldrich, SLSV025LS and SLHV033RB) to remove debris and cellular content. Virus-like particles were precipitated by overnight incubation at 10% w/v PEG-8000 (Fisher Scientific, BP233) and resuspended in saline-magnesium buffer. Remaining bacterial cells were lysed by treatment with 1 mg/ml lysozyme (Sigma, L4919) for 30 min at 37°C followed by 0.2 volumes of chloroform (10 min, room temperature). After centrifugation (5 min, 2500 *g*), the aqueous mixture was treated with TURBO DNase (Thermo Scientific, AM2238) and RNaseI (Life Technologies, EN0602) in a buffer of 1 mM CaCl_2_ and 5 mM MgCl_2_ for 1 h at 37°C to degrade remaining bacterial nucleic acids. Enzymes were inactivated at 70°C for 10 minutes. Virus-like particles were lysed with Proteinase K (3.2 µg/ml; Fisher Scientific, BP1700) in 3.2% SDS for 20 minutes at 55°C, then treated by 2.5% cetyltrimethylammonium bromide (Fisher Scientific, O3042) with 0.5 M NaCl for 10 minutes at 65°C. Viral DNA was then extracted by adding 1 volume of phenol-chloroform-isoamyl alcohol (25:24:1, pH 6.7) to each mixture, which was vortexed and subjected to centrifugation (10 min, 8000 *g*); this step was repeated with chloroform to remove trace phenol. Nucleic acids were purified from the aqueous layer using the Dneasy Blood and Tissue Kit (QIAGEN, 69506) and eluted in 50 µl of water. DNA was concentrated using an Eppendorf™ Vacufuge™ Concentrator to 3 µl to maximize the input DNA for the GenomiPhi^TM^ V2 DNA Amplification polymerase kit (GE Life Science, 25660032). Reactions using 1 µl of input DNA were run in triplicate, then pooled and purified with the Dneasy Blood and Tissue Kit. DNA was quantified fluorescently using the Qubit dsDNA HS Assay Kit (Thermo Fisher, Q32854).

### Whole Metagenomic and Metatranscriptomic Extraction

Whole metagenomic DNA was extracted using the FastDNA Spin Kit for DNA Isolation (MP Biomedicals, 116540600), eluted in water, and quantified using the Qubit High Sensitivity dsDNA Assay Kit as previously described.^67^

Total RNA was freshly extracted from pellets obtained from MLI aspirates, using a modified phenol/chloroform extraction protocol adapted for samples with high mucin content.^68^ Briefly, fresh 20 ml MLI aliquots were subjected to two sequential centrifugations at 4°C (700 *g* for 5 min; 13000 rpm for 20 min) to remove cellular debris and pellet bacterial cells. The pellets were resuspended in denaturing buffer (4 M guanidine thiocyanate, 25 mM sodium citrate, 0.5% N-lauroylsarcosine, 1 M 2-mercaptoethanol, 1% N-acetyl cysteine) and 0.1 volumes of 1 M sodium acetate pH 5.2. The samples were incubated at 65°C for 2 min to lyse the cells, preheated buffer-saturated phenol pH 4.3 was added at a 1:1 ratio and incubated at 65°C for 10 min with frequent inverting. The samples were then chilled by placing them directly on ice for 15 min, chloroform was added (0.5 volumes) and the tubes inverted 10 times. The aqueous phase was separated by centrifugation at 13000 rpm for 25 min at 4°C and the RNA precipitated at −80°C by adding 2.5 volumes of ethanol, 0.1 volumes 3 M sodium acetate pH 5.2 and EDTA added to a final concentration of 1 mM. Precipitated RNA extractions were further cleaned by washing the pellets 5 times with chilled 80% ethanol with 5 min 13000 rpm centrifugations at 4°C. The RNA was then resuspended in Rnase free water (Ambion, AM9937) and stored at −80°C. Contaminating DNA was removed using sequential Dnase I (Thermo Scientific, EN0521) treatments and further purified and concentrated using the RNA Clean & Concentrator-25 (Zymo Research, R1017). The absence of human and bacterial DNA was then assessed using PCR with primers targeting human actin (hACTBf2: AGTCCTACGGAAAACGGCAG, hACTBr2: CACCCTGAAGTACCCCATCG) and the bacterial V4-16S rRNA hypervariable region (V4_bc45: CCATCTCATCCCTGCGTGTCTCCGACTCAGTAACGCGTTCGAYTGGGYD TAAAGNG, V4_revs: CCTCTCTATGGGCAGTCGGTGATTA CNVGGGTATCTAATCC).^67^ PCR products were analyzed on a 1% agarose gel and the Dnase I treatment repeated if a band was present. RNA quantity and quality were evaluated using the Qubit RNA HS Assay Kit (Thermo Fisher, EN0602) and the RNA 6000 Nano Kit (Agilent Technologies, 5067–1511) on the 2100 Bioanalyzer. Samples with an RNA Integrity Number (RIN) ≥ 7 were deemed suitable for sequencing.

### DNA sequencing, quality-filtering, and host-read removal

DNA and RNA sequencing were performed at the Génome Québec CES using the NEB Ultra II and NEB rRNA-depleted stranded library preparation kits, respectively, with subsequent sequencing on the Illumina NovaSeq 6000 platform. Cutadapt 2.10 was used to trim Illumina’s universal adapters (AGATCGGAAGAG; AGATCGGAAGAG) while Trimmomatic 0.36 was used in paired-end mode to retain high-quality sequences with the settings SLIDINGWINDOW:4:20, MINLEN:60, and HEADCROP:10. Reads mapping to the human genome (GRCh38 with bowtie2ʹs ultra-sensitive mode)^69^ were removed from further analysis using samtools 1.7.^70^ Finally, low complexity reads were removed using Komplexity.^71^ Bacterial contamination was assessed by aligning reads to the *cpn60* database with bowtie2ʹs ultra-sensitive mode.^27^

An average of 230 million paired end reads (173–280 million) were obtained for each metavirome sample, totaling 34 Gbps, while an average of 154 million paired end reads (137–177 million) were obtained for each metagenome sample, totaling 23 Gbps. Quality filtering resulted in the removal of 6.16% of reads (4.91–9.10%) across all 18 samples, with no significant difference between metavirome and metagenome sequencing. Host read filtering removed 0.020–0.319% of high-quality metavirome reads and 0.0849–94.3% of high-quality metagenomic reads (p = .0005 between the two groups). Low complexity sequence filtering, which removes human microsatellite DNA and aids downstream assembly and analysis, was performed using Komplexity, which removed another 0.235–5.79% high-quality reads.^71^ Overall, this resulted in a mean of 211 (157–254) million paired-end metavirome reads and 75.4 (6.83–162) million paired-end metagenomic reads per sample.

### Metatranscriptomic analysis

Metatranscriptome sequencing reads were subject to adapter trimming and quality filtering using cutadapt and Trimmomatic as described above. TopHat2 was used to identify reads aligning to human transcripts and these were removed using samtools 1.9.^70,72^ SortMeRNA and Komplexity was then used to remove ribosomal and low complexity reads, resulting in remaining high-quality, non-human, and non-ribosomal reads.^73^ For RdRp and ssRNA marker identification, MEGAHIT v1.2.7 was used to assemble these reads at default settings (minimum contig length of 200 bp). ORFs were predicted using Prodigal 2.6.3 in its metagenomic mode,^74^ and searched against databases of RNA-dependent RNA polymerases and capsids used by Cenote-Taker 2.1.3 and ssRNA marker genes using hmmsearch with a minimum E-value of 1E-05.^1,14,75^

### Metagenomic assembly, viral contig identification, and viral gene annotation

The bioinformatic pipeline for VC identification is summarized in Supplementary Figure 2. Host-decontaminated, quality-filtered reads from each metagenome and metavirome sample were assembled using MEGAHIT v1.2.7 with a minimum contig length of 3000 bp.^76^ Metagenome and metavirome assemblies were each pooled and clustered using ClusterGenomes at 95% identity across 90% of the length of the smaller contig.^77^ Viral contigs were identified using Cenote-Taker2 using default settings for whole metagenomic assembly (including pruning of flanking host regions) and virus-like particle preparation assembly, respectively. To examine the relationship between sequencing depth, this process was repeated with quality-filtered, metaviromic sequencing reads subsetted at increments from 10,000 to 100 million paired end reads per sample to simulate various sequencing depths.

All putative viral contigs were further pooled and clustered, resulting in 2,171 VCs. During this step, all metavirome-derived VCs were also clustered against the set of metagenomic non-viral contigs: non-viral contigs clustering within a viral contig were removed from the set of non-viral contigs, while metavirome-derived viral contigs clustering within non-viral contigs were considered prophages (along with host DNA flanked prophages identified in metagenome). Non-viral contigs that contained one or more prophages had these regions masked. Whole metagenomic assemblies that were not identified as viral contigs (n = 39,735) were annotated using the Contig Annotation Tool.^78^

VCs were assessed using CheckV,^30^ annotated using Demovir,^29^ then clustered with vContact2.^31^ Members of a viral cluster were annotated by majority vote. As Demovir has not yet been updated to match the latest viral taxonomy established by ICTV, we manually removed reference to the families *Siphoviridae, Myoviridae*, and *Podoviridae*, but opted to retain references to the order *Caudovirales* (now class *Caudoviricetes*) to allow for straightforward comparisons to previous published datasets. Open reading frame (ORF) prediction was performed using Prokka 1.14.5 with – meta enabled and – kingdom Bacteria or – Viruses against the set of non-viral and viral contigs, respectively.^79^ All open reading frames were clustered using Mmseqs2 13.45111,^80^ and annotated using hmmsearch (minimum E-value of 1E-05), a part of HMMER 3.3,^75^ against the following databases: VOG (release 206),^41^ pVOG,^42^ PFAM (34.0),^43^ KEGG (98.0),^44^ and TIGRFAM (15.0).^45^ Genes were preferentially annotated in the order of the databases listed above. crAss-like phages were additionally annotated using a curated set of crAss-like phage protein profiles.^51^ SNP analysis was performed on un-clustered crAss-like phages using Snippy 4.6.0.^50^ A viral contig was considered to contain an integrase if it contained one or more of the 603 genes annotated with the term “integrase”; the majority of these (591/603) were annotated by VOG00035.

All metagenomic, metatranscriptome, and metaviromic reads were mapped to the pooled set of viral and non-viral contigs using bowtie2,^81,82^ with counts and breadth of coverage calculated using samtools depth.^70^ A breadth of coverage threshold of ≥75% of the contig should have a minimum depth of ≥1 read per bp was applied to metagenome and metavirome count tables; counts under this threshold was set to 0. The normalized relative abundance (NRA) of each VC was calculated as $\frac{x_{i}}{\sum_{x_{i}}}$ where x_i_ is the number of reads mapping to a contig divided by the contig length. Gene counts were calculated using subread featureCounts.^83^

### Viral gene transcription and host prediction

A viral contig was considered to be transcriptionally active if its NRA in the metatranscriptome was greater than 0.0001%. Gene features within transcriptionally active VCs with >1 counts were considered to be transcribed. Gene transcription was approximated using the ratio of NRA in the metatranscriptome to NRA in the metagenome.

WiSH 1.1 was run using the set of non-viral contigs against VCs.^84^ Only virus-host scores with an adjusted p-value < 10^–5^ were selected for downstream analysis. Correlation with their viral hosts was calculated using a Spearman correlation.

### Statistical analysis

Alpha-diversity and beta-diversity analysis, host-phage correlations, statistical analysis, and plotting were performed in R 4.1.3 using phyloseq 1.36.0.^85^ Figures were created with the following packages: ggplot2 3.3.0, ggthemes 4.2.0, ggpubr 0.4.0, gggenes 0.4.1, ggalluvial 0.12.3, eulerr 6.1.0, and UpSetR 1.4.0. Alpha diversity analysis was performed using read counts rarefied to 1,104,709 reads (the lowest number of viral reads identified in a sample across the dataset). The Chao1 index was calculated as $S_{0}+\frac{a_{1}^{2}}{2a_{2}}$ where *S_0_* was the total observed species, *a_1_* the number of species seen once and *a_2_* the number of species seen twice; and the Shannon index was calculated as $-\overset{S}{\underset{i}{\sum }}P_{i\;}\ln P_{i}$ where *S* was the total number of species and *P_i_* the proportion of the total population composed of species *i*. The likelihood ratio was calculated as Sensitivity/(1 – Specificity).

## Supplementary Material

Supplemental Material

## Data Availability

Host-removed, high-quality sequencing reads are available under BioProject PRJNA81830 https://www.ncbi.nlm.nih.gov/bioproject/PRJNA818303.

## Abbreviations

DC, distal colon; MLI, mucosal-luminal interface; IC, integration-capable; NI, non-integrated; NRA, normalized relative abundance; ORF: open reading frame; PC, proximal colon; STL: stool; TA, transcriptionally active; VC: viral contig; VLP: virus-like particle
