# Supplementary material for: Multiomic spatial analysis reveals a distinct mucosa-associated virome
Source: Gut Microbes. 2023 Feb 23;15(1):2177488. doi: 10.1080/19490976.2023.2177488 (PMC9980608; doi:10.1080/19490976.2023.2177488)
Supplement: Supplemental Material [file KGMI_A_2177488_SM8988.zip › Supplemental Information revised.pdf]

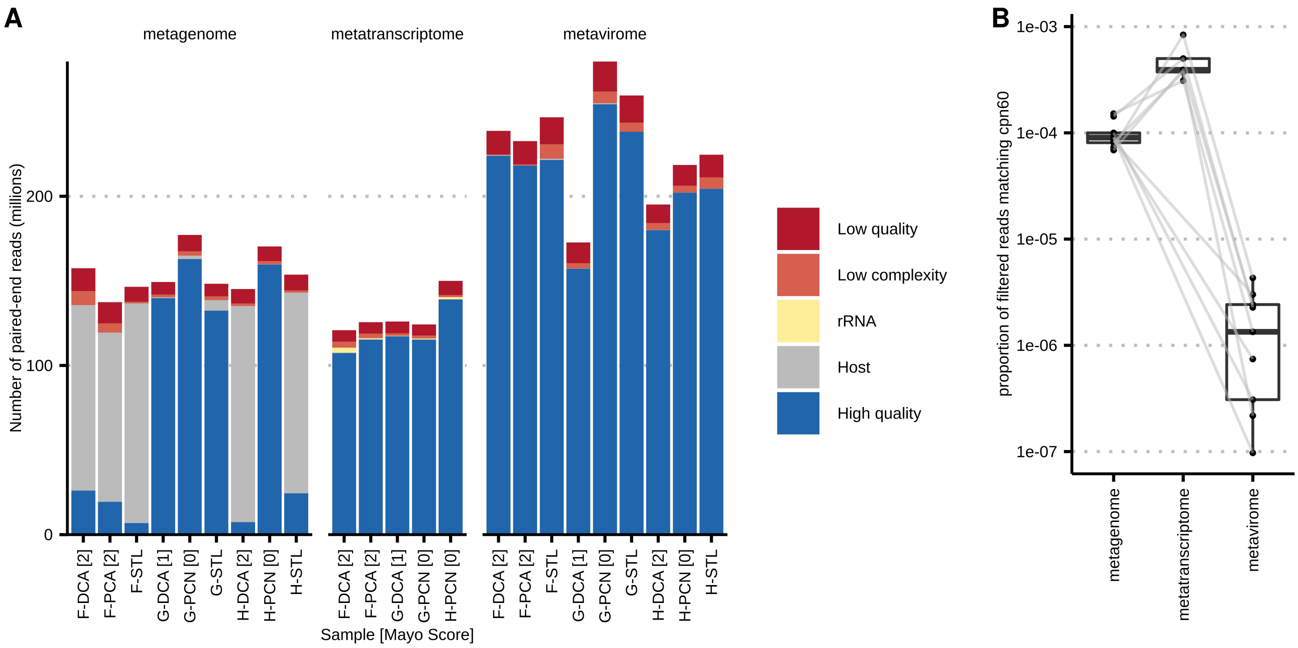

Supplementary Figure 1: Processing of raw metagenomic, metatranscriptomic, and metaviromic sequencing reads. (A) Bar plot showing the filtering of raw sequencing reads based on quality, host contamination, low-complexity, and rRNA (metatranscriptome only) for each sample, which includes the participant, site (PC: proximal colon; DC: distal colon; STL: stool), and inflammation status (A: affected; N: non-inflamed). For aspirate samples, the Mayo endoscopic score of the collection site is noted in square brackets. (B) Boxplot showing presence of *cpn60* genes in high-quality multiomic sequencing datasets to assess bacterial contamination in the VLP-filtered metavirome as compared to the metagenome and metatranscriptome with matched samples connected with lines.

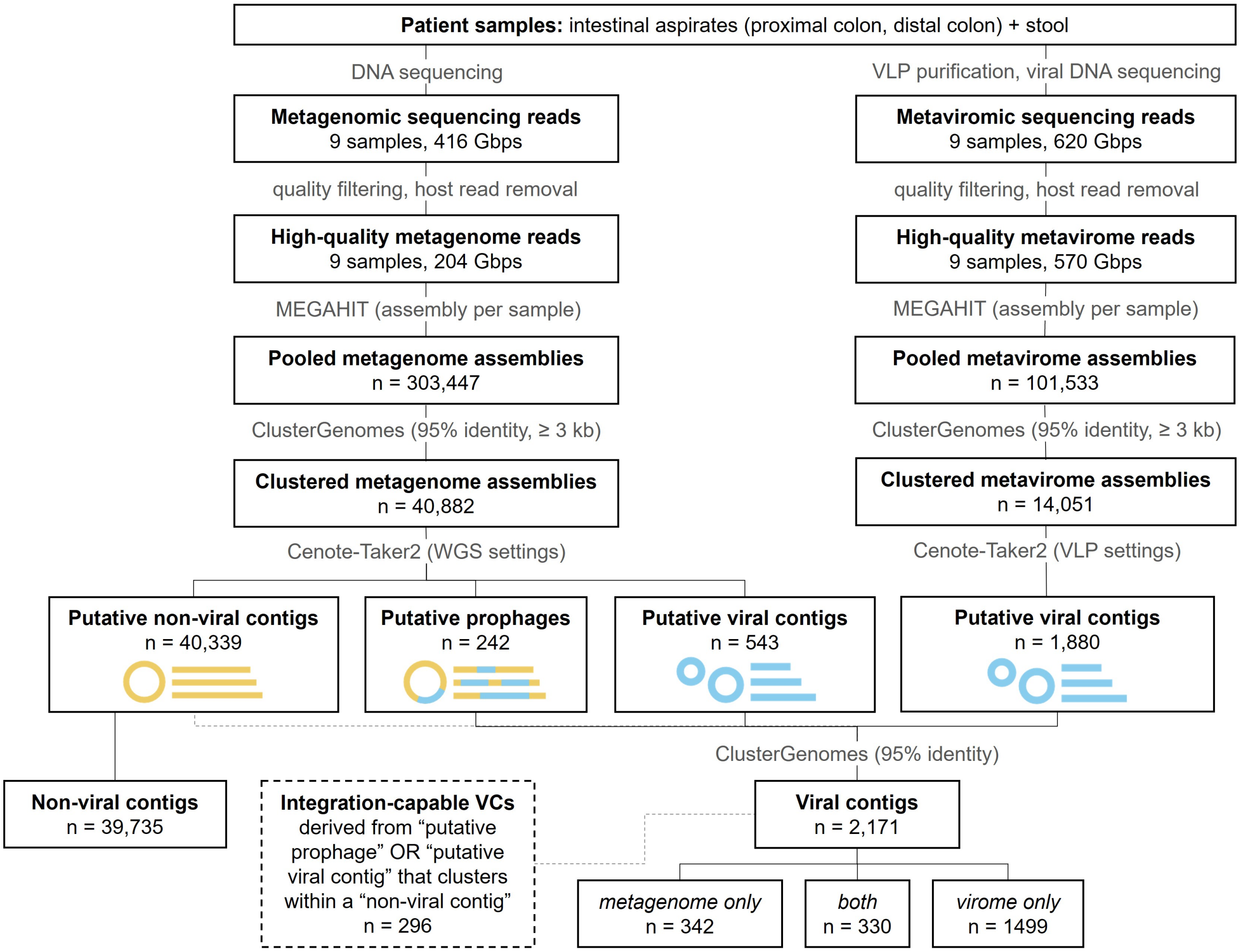

Supplementary Figure 2. Metagenomic and metaviromic sequencing pipelines for assembly and viral contig identification. High-quality sequencing reads were assembled per sample using MEGAHIT, then pooled and clustered with ClusterGenomes. These clusters were then subjected to viral sequence identification with Cenote-Taker2, utilizing WGS and VLP oriented settings for the metagenome and metavirome clusters, respectively; metagenome assemblies that were not identified as viral were classified as putative non-viral contigs. Flanking regions of putative prophages were also considered separately as non-viral contigs. Putative viral contigs and prophages from metagenome and metavirome sequencing were further clustered resulting in 2,171 viral contigs; the numbers of contigs isolated from the metagenome, metavirome, or both are specified. Additionally, viral contigs that were either derived from a putative prophage (i.e. with flanking host regions) or derived from a viral contig clustering with a non-viral contig (thus inferring flanking host regions) were identified as integration-capable VCs, representing 296 of the 2,171 VCs.

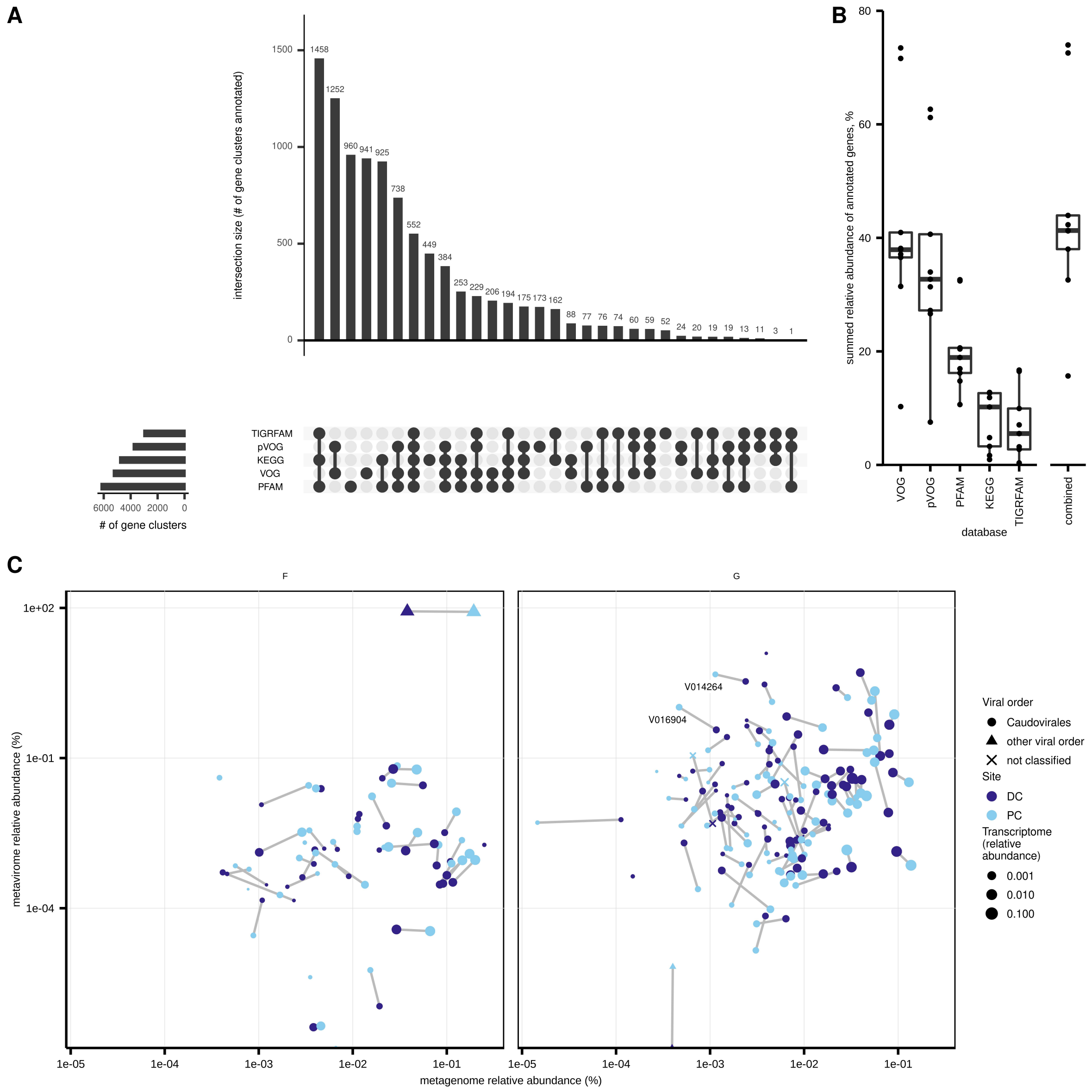

Supplementary Figure 3: Annotating viral gene clusters. (A) Plot showing the sets of the 9,647 viral gene clusters that were annotated using viral protein family databases (VOG, pVOG) and general purpose databases (PFAM, TIGRFAM, KEGG); the remaining 18,465 gene clusters were annotated. (B) Boxplot showing the combined relative abundance of annotated genes in each metaviromic sample, by individual databases and all combined databases. (C) Scatter plots highlighting transcriptionally active VCs between proximal and distal colon samples in participants F and G. Lines indicate VCs present at both sites. The two mucosa-associated crAss-like phages identified in this study are labelled.

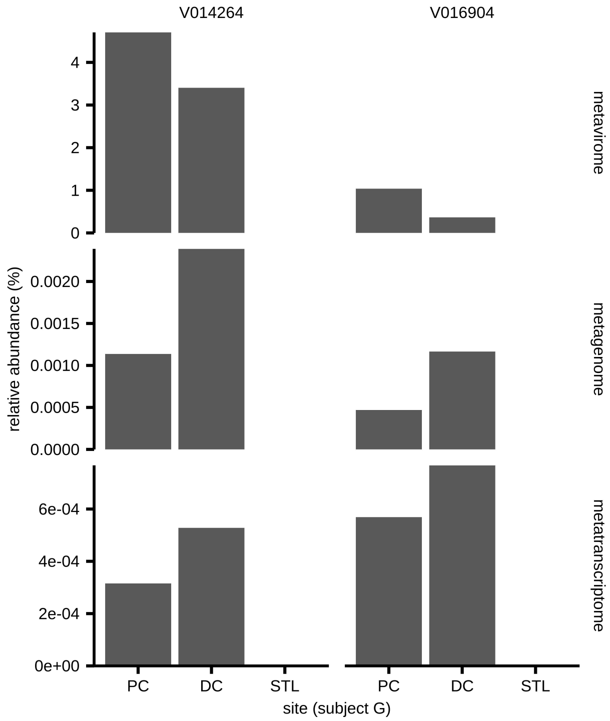

Supplementary Figure 4: Two crAss-like phages highly abundant in the colonic mucosal-luminal interface. Relative abundance of crAss-like phages V014264 and V016904 across the metavirome, metagenome, and metatranscriptome of participant G. No metatranscriptome samples were obtained from stool. PC: proximal colon; DC: distal colon; STL: stool.

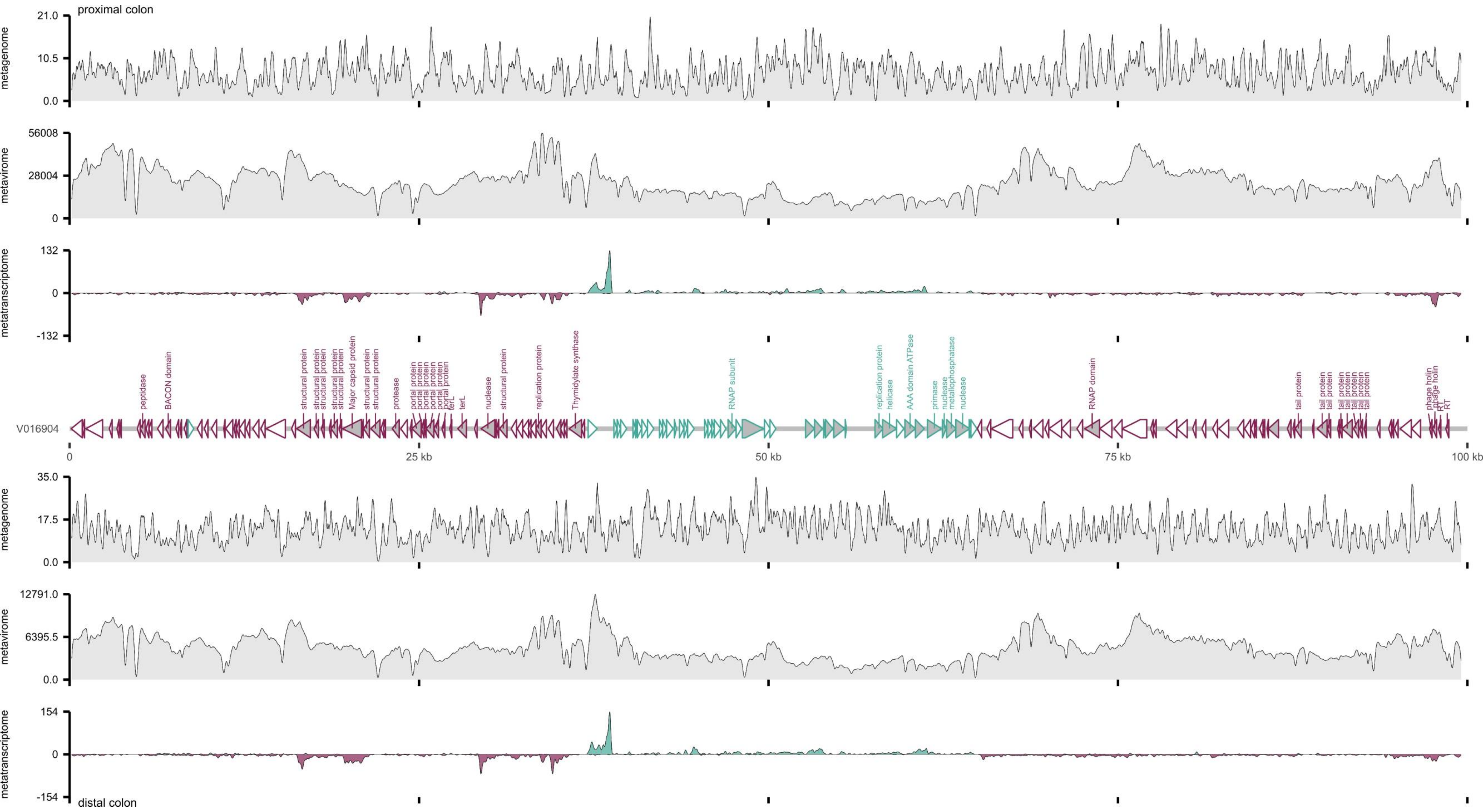
proximal colon
metagenome
metavirome
metatranscriptome
V016904
peptidase
BACON domain
structural protein
Major capsid protein
protease
portal protein
terL
nuclease
replication protein
Thymidylate synthase
RNAP subunit
helicase
AAA domain ATPase
primase
metallophosphatase
RNAP domain
tail protein
phage holin
RT
0
25 kb
50 kb
75 kb
100 kb
distal colon

Supplementary Figure 5: Multiomic sequencing map of crAss-like phage V016904. Open reading frames (ORFs) were predicted over the 99.5 kb genome coloured and oriented by forward (green) and reverse (purple) orientation. Annotated ORFs are shaded in gray and labelled (excluding annotations of ‘uncharacterized’ or ‘hypothetical’ proteins). Metagenome, metavirome, and metatranscriptome sequencing depths of the proximal colon (top) and distal colon (below) are plotted with a 151 bp sliding window; metatranscriptome reads were mapped by strand.

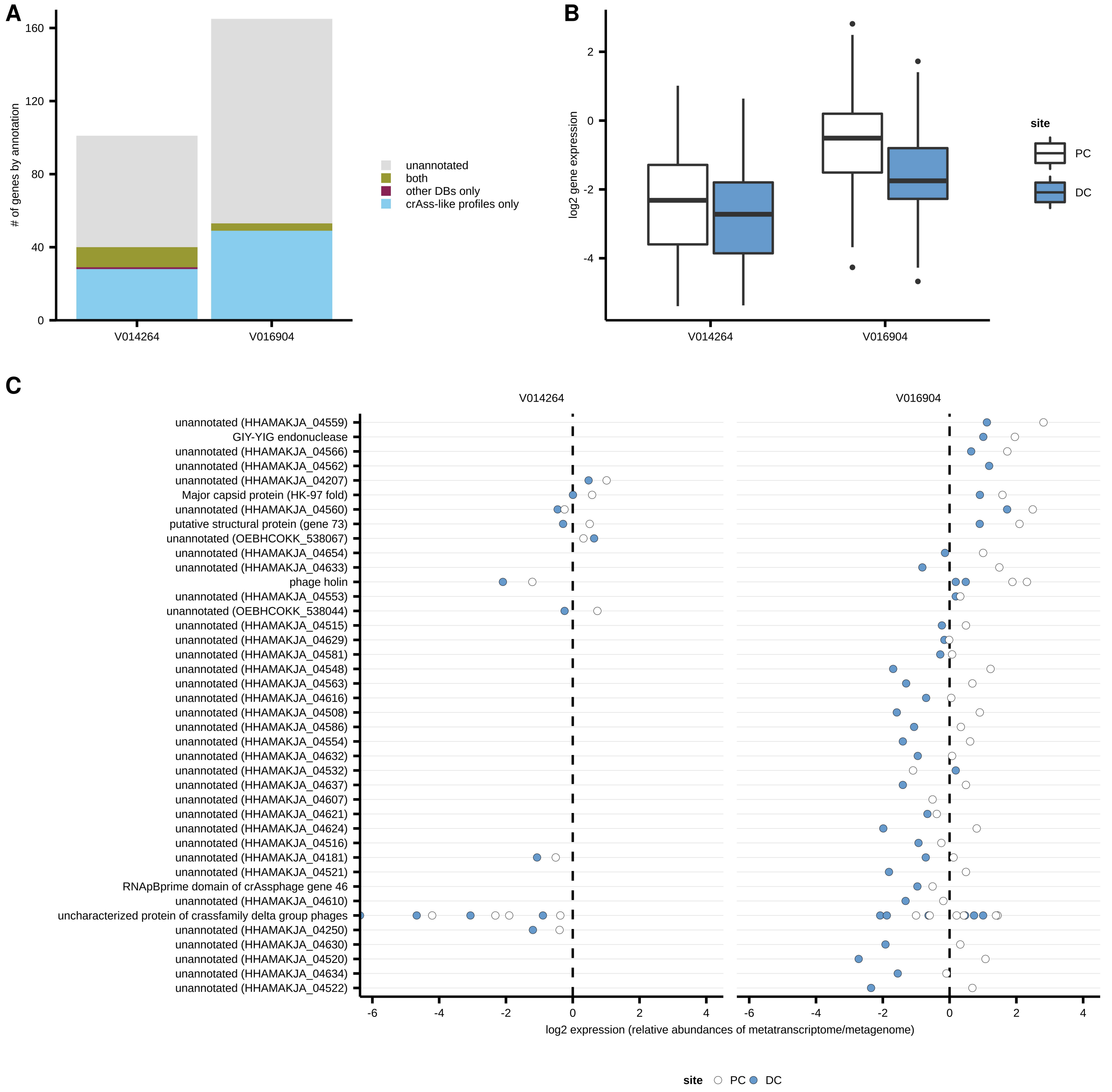

Supplementary Figure 6: Gene annotation and expression of two crAss-like phages. (A) The number of genes in each crAss-like phage that could be annotated using a curated set of crAss-like phage profiles as compared to other viral and generic databases. (B) The expression (ratio of metatranscriptome / metagenome counts) of expressed genes for V014264 and V016904, by sample site of participant G. (C) The 30 most expressed crAss-like phage gene families identified in the proximal and distal colon of participant G. The dashed line indicates an metatranscriptome / metagenome ratio of 1.

Supplementary Table 1: 2,171 viral contigs identified in the metagenome and metavirome, with annotations regarding source, taxonomy, presence of integrase or flanking regions, and maximum abundance in any given metaviromics sample (table provided separately).
