# Supplementary material for: Multiomic spatial analysis reveals a distinct mucosa-associated virome
Source: Gut Microbes. 2023 Feb 23;15(1):2177488. doi: 10.1080/19490976.2023.2177488 (PMC9980608; doi:10.1080/19490976.2023.2177488)
Supplement: Supplemental Material [file KGMI_A_2177488_SM8988.zip › Supplementary_Table_1.pdf]

viralcontig	checkv_quality	source	vc_order	vc_family
P000424	high-quality	metagenome_only	Caudovirales	Siphoviridae
P000565	high-quality	both	Caudovirales	Myoviridae
P000791	low-quality	metagenome_only	Caudovirales	Siphoviridae
P001087	low-quality	metagenome_only	Caudovirales	Unassigned
P001186	low-quality	metagenome_only	Caudovirales	Myoviridae
P001233	low-quality	metagenome_only	Caudovirales	Siphoviridae
P001414	low-quality	metagenome_only	Caudovirales	Siphoviridae
P001722	low-quality	metagenome_only	Caudovirales	Siphoviridae
P001815	low-quality	both	Caudovirales	Unassigned
P001935	medium-quality	both	Caudovirales	Siphoviridae
P002202	low-quality	metagenome_only	Caudovirales	Siphoviridae
P002272	low-quality	both	Caudovirales	Unassigned
P002417	low-quality	metagenome_only	Caudovirales	Siphoviridae
P002496	medium-quality	metagenome_only	Caudovirales	Myoviridae
P003154	high-quality	both	Caudovirales	Siphoviridae
P003520	low-quality	both	Caudovirales	Siphoviridae
P003861	low-quality	both	Caudovirales	Unassigned
P004050	low-quality	metagenome_only	Caudovirales	Siphoviridae
P004138	high-quality	metagenome_only	Caudovirales	Siphoviridae
P004187	low-quality	metagenome_only	Caudovirales	Myoviridae
P004562	medium-quality	both	Caudovirales	Siphoviridae
P004614	high-quality	both	Caudovirales	Siphoviridae
P004629	low-quality	metagenome_only	Caudovirales	Siphoviridae
P004803	low-quality	metagenome_only	Caudovirales	Myoviridae
P004850	medium-quality	metagenome_only	Caudovirales	Siphoviridae
P004884	medium-quality	metagenome_only	Caudovirales	Siphoviridae
P005687	low-quality	metagenome_only	Caudovirales	Myoviridae
P006419	high-quality	both	Caudovirales	Unassigned
P006799	low-quality	metagenome_only	Caudovirales	Unassigned
P006997	low-quality	metagenome_only	Caudovirales	Myoviridae
P007051	low-quality	metagenome_only	Caudovirales	Siphoviridae
P007053	complete	both	Caudovirales	Siphoviridae
P007611	low-quality	metagenome_only	Caudovirales	Siphoviridae
P008402	medium-quality	metagenome_only	Caudovirales	Myoviridae
P008570	low-quality	metagenome_only	Caudovirales	Unassigned
P008607	complete	both	Caudovirales	Siphoviridae
P008694	high-quality	metagenome_only	Caudovirales	Siphoviridae
P008865	medium-quality	metagenome_only	Caudovirales	Myoviridae
P009310	high-quality	both	Caudovirales	Siphoviridae
P009353	low-quality	metagenome_only	Caudovirales	Myoviridae
P009564	low-quality	both	Caudovirales	Myoviridae
P009638	low-quality	metagenome_only	Caudovirales	Myoviridae
P010499	low-quality	metagenome_only	Caudovirales	Myoviridae
P011181	low-quality	metagenome_only	Caudovirales	Unassigned
P011241	low-quality	both	Caudovirales	Siphoviridae
P011650	medium-quality	both	Caudovirales	Myoviridae

P011707	complete	both	Petitvirales	Microviridae
P011875	low-quality	both	Caudovirales	Siphoviridae
P012007	low-quality	metagenome_only	Caudovirales	Siphoviridae
P012138	low-quality	metagenome_only	Caudovirales	Siphoviridae
P012506	medium-quality	metagenome_only	Caudovirales	Siphoviridae
P012518	low-quality	metagenome_only	Caudovirales	Unassigned
P012912	low-quality	metagenome_only	Caudovirales	Myoviridae
P012941	medium-quality	both	Caudovirales	Siphoviridae
P013049	low-quality	metagenome_only	Caudovirales	Siphoviridae
P013213	medium-quality	metagenome_only	Caudovirales	Myoviridae
P013317	medium-quality	both	Caudovirales	Siphoviridae
P013984	medium-quality	both	Caudovirales	Siphoviridae
P014109	low-quality	both	Caudovirales	Siphoviridae
P014112	low-quality	metagenome_only	Caudovirales	Siphoviridae
P014136	medium-quality	metagenome_only	Caudovirales	Siphoviridae
P014147	low-quality	metagenome_only	Caudovirales	Siphoviridae
P014314	low-quality	metagenome_only	Caudovirales	Myoviridae
P014402	high-quality	both	Caudovirales	Siphoviridae
P014414	low-quality	metagenome_only	Caudovirales	Myoviridae
P014690	low-quality	metagenome_only	Caudovirales	Unassigned
P014858	low-quality	both	Caudovirales	Podoviridae
P014859	low-quality	metagenome_only	Caudovirales	Siphoviridae
P015082	low-quality	both	Caudovirales	Siphoviridae
P015761	low-quality	metagenome_only	Caudovirales	Siphoviridae
P016467	low-quality	metagenome_only	Caudovirales	Siphoviridae
P016530	low-quality	metagenome_only	Caudovirales	Siphoviridae
P017180	high-quality	both	Caudovirales	Podoviridae
P017622	low-quality	metagenome_only	Caudovirales	Myoviridae
P018038	low-quality	metagenome_only	Caudovirales	Siphoviridae
P018071	medium-quality	metagenome_only	Caudovirales	Siphoviridae
P018200	low-quality	metagenome_only	Caudovirales	Siphoviridae
P018503	medium-quality	metagenome_only	Caudovirales	Siphoviridae
P018677	low-quality	metagenome_only	Caudovirales	Siphoviridae
P018716	medium-quality	metagenome_only	Caudovirales	Siphoviridae
P018732	low-quality	metagenome_only	Caudovirales	Siphoviridae
P018805	high-quality	both	Caudovirales	Siphoviridae
P018997	medium-quality	metagenome_only	Caudovirales	Siphoviridae
P019622	low-quality	metagenome_only	Caudovirales	Unassigned
P020373	low-quality	metagenome_only	Caudovirales	Myoviridae
P020404	low-quality	metagenome_only	Caudovirales	Myoviridae
P021003	low-quality	metagenome_only	Caudovirales	Siphoviridae
P021113	high-quality	both	Caudovirales	Siphoviridae
P021890	low-quality	metagenome_only	Caudovirales	Siphoviridae
P021937	low-quality	both	Caudovirales	Siphoviridae
P022926	low-quality	metagenome_only	Caudovirales	Myoviridae
P023102	low-quality	metagenome_only	Caudovirales	Siphoviridae
P023129	low-quality	metagenome_only	Caudovirales	Siphoviridae

P023550	low-quality	metagenome_only	Caudovirales	Siphoviridae
P023770	low-quality	metagenome_only	not classified	not classified
P023882	low-quality	metagenome_only	Caudovirales	Unassigned
P023902	low-quality	both	Caudovirales	Siphoviridae
P023999	low-quality	metagenome_only	Caudovirales	Siphoviridae
P024033	low-quality	metagenome_only	Caudovirales	Siphoviridae
P024250	low-quality	metagenome_only	Caudovirales	Siphoviridae
P024435	low-quality	metagenome_only	Caudovirales	Myoviridae
P024706	low-quality	both	not classified	not classified
P025181	low-quality	metagenome_only	Caudovirales	Siphoviridae
P025362	medium-quality	metagenome_only	Caudovirales	Unassigned
P025893	low-quality	metagenome_only	Caudovirales	Myoviridae
P026450	medium-quality	both	Caudovirales	Siphoviridae
P026689	complete	both	Caudovirales	Podoviridae
P026769	complete	both	Caudovirales	Siphoviridae
P026791	low-quality	metagenome_only	Caudovirales	Myoviridae
P026942	low-quality	metagenome_only	Caudovirales	Siphoviridae
P026974	high-quality	both	Caudovirales	Myoviridae
P027054	medium-quality	metagenome_only	Caudovirales	Siphoviridae
P027284	low-quality	metagenome_only	Caudovirales	Siphoviridae
P027295	low-quality	metagenome_only	not classified	not classified
P027319	low-quality	metagenome_only	Caudovirales	Siphoviridae
P027459	low-quality	metagenome_only	Caudovirales	Unassigned
P027572	low-quality	metagenome_only	Caudovirales	Siphoviridae
P027721	low-quality	both	Caudovirales	Siphoviridae
P027862	low-quality	metagenome_only	Caudovirales	Siphoviridae
P028057	not-determined	metagenome_only	Caudovirales	Unassigned
P028454	low-quality	metagenome_only	Caudovirales	Siphoviridae
P028711	low-quality	metagenome_only	not classified	not classified
P028871	low-quality	metagenome_only	Caudovirales	Siphoviridae
P029577	medium-quality	metagenome_only	Caudovirales	Siphoviridae
P029706	low-quality	both	Caudovirales	Siphoviridae
P029719	low-quality	metagenome_only	Caudovirales	Siphoviridae
P030067	low-quality	metagenome_only	Caudovirales	Siphoviridae
P030112	medium-quality	both	Caudovirales	Siphoviridae
P030471	high-quality	both	Caudovirales	Siphoviridae
P030917	low-quality	both	Caudovirales	Podoviridae
P030959	medium-quality	metagenome_only	Caudovirales	Siphoviridae
P031138	medium-quality	both	Caudovirales	Siphoviridae
P031176	high-quality	both	Caudovirales	Myoviridae
P031251	medium-quality	metagenome_only	Caudovirales	Myoviridae
P031465	high-quality	both	Caudovirales	Siphoviridae
P031774	high-quality	both	Caudovirales	Siphoviridae
P031845	low-quality	metagenome_only	Caudovirales	Siphoviridae
P032059	low-quality	metagenome_only	Caudovirales	Unassigned
P032134	low-quality	metagenome_only	Caudovirales	Unassigned
P032684	low-quality	metagenome_only	Caudovirales	Unassigned

P033107	low-quality	metagenome_only	Caudovirales	Unassigned
P033250	low-quality	metagenome_only	Caudovirales	Unassigned
P033779	not-determined	metagenome_only	no_order_Phycodnaviridae	Phycodnaviridae
P034251	high-quality	metagenome_only	Caudovirales	Siphoviridae
P034884	medium-quality	both	Caudovirales	Siphoviridae
P035171	low-quality	metagenome_only	Caudovirales	Myoviridae
P036020	high-quality	both	Caudovirales	Siphoviridae
P036083	medium-quality	both	Caudovirales	Siphoviridae
P036218	high-quality	both	Caudovirales	Siphoviridae
P036262	medium-quality	metagenome_only	Caudovirales	Myoviridae
P036535	low-quality	metagenome_only	Caudovirales	Siphoviridae
P037012	low-quality	metagenome_only	Caudovirales	Podoviridae
P037356	low-quality	metagenome_only	Caudovirales	Siphoviridae
P037383	complete	both	Caudovirales	Myoviridae
P037439	high-quality	both	Caudovirales	Myoviridae
P037501	low-quality	metagenome_only	Caudovirales	Myoviridae
P037656	high-quality	metagenome_only	not classified	not classified
P038442	medium-quality	both	Caudovirales	Siphoviridae
P038469	low-quality	both	Caudovirales	Unassigned
P039352	low-quality	metagenome_only	Caudovirales	Siphoviridae
P039356	low-quality	metagenome_only	Caudovirales	Siphoviridae
P039967	low-quality	metagenome_only	Caudovirales	Unassigned
P040044	high-quality	both	Caudovirales	Siphoviridae
P040056	low-quality	metagenome_only	Caudovirales	Siphoviridae
P040293	low-quality	both	Caudovirales	Siphoviridae
P040589	low-quality	both	Caudovirales	Siphoviridae
P040862_1	low-quality	metagenome_only	Caudovirales	Myoviridae
P040862_2	medium-quality	metagenome_only	Caudovirales	Myoviridae
P041290	low-quality	metagenome_only	Caudovirales	Unassigned
P041295	medium-quality	both	Caudovirales	Siphoviridae
P041675	high-quality	both	Caudovirales	Siphoviridae
P041895	low-quality	both	Caudovirales	Unassigned
P041976	medium-quality	both	Caudovirales	Myoviridae
P042280	not-determined	metagenome_only	Caudovirales	Unassigned
P042396	low-quality	metagenome_only	Caudovirales	Siphoviridae
P042601	medium-quality	metagenome_only	Caudovirales	Siphoviridae
P043135	low-quality	metagenome_only	Caudovirales	Siphoviridae
P043186	low-quality	both	Caudovirales	Myoviridae
P043445	medium-quality	both	Caudovirales	Siphoviridae
P043464	low-quality	metagenome_only	Caudovirales	Siphoviridae
P043501	low-quality	metagenome_only	Caudovirales	Siphoviridae
P044308	low-quality	metagenome_only	Caudovirales	Myoviridae
P044419	high-quality	metagenome_only	Caudovirales	Siphoviridae
P045514	low-quality	metagenome_only	Caudovirales	Siphoviridae
P045596	medium-quality	both	Caudovirales	Siphoviridae
P045665	low-quality	metagenome_only	Caudovirales	Siphoviridae
P046544	complete	both	Caudovirales	Siphoviridae

P046809	low-quality	metagenome_only	Caudovirales	Myoviridae
P047048	medium-quality	both	Caudovirales	Podoviridae
P047147	low-quality	metagenome_only	Caudovirales	Myoviridae
P047308	medium-quality	metagenome_only	Caudovirales	Siphoviridae
P047339	low-quality	metagenome_only	Caudovirales	Siphoviridae
P047355	medium-quality	both	Caudovirales	Myoviridae
P047533	low-quality	metagenome_only	Caudovirales	Siphoviridae
P047585	high-quality	both	Caudovirales	Siphoviridae
P047724	medium-quality	metagenome_only	Caudovirales	Siphoviridae
P047736	high-quality	both	Caudovirales	Siphoviridae
P047963	low-quality	metagenome_only	Caudovirales	Siphoviridae
P048258	low-quality	metagenome_only	Caudovirales	Unassigned
P048387	medium-quality	metagenome_only	Caudovirales	Siphoviridae
P049274	low-quality	both	Caudovirales	Unassigned
P049358	medium-quality	both	Caudovirales	Siphoviridae
P049505	low-quality	both	Caudovirales	Siphoviridae
P049506	medium-quality	metagenome_only	Caudovirales	Siphoviridae
P049507	low-quality	metagenome_only	Caudovirales	Siphoviridae
P049529	medium-quality	metagenome_only	Caudovirales	Myoviridae
P049757	low-quality	metagenome_only	Caudovirales	Siphoviridae
P049778	high-quality	both	Caudovirales	Siphoviridae
P049924	low-quality	metagenome_only	Caudovirales	Unassigned
P050156	not-determined	metagenome_only	not classified	not classified
P050442	high-quality	both	Caudovirales	Siphoviridae
P050516	high-quality	both	Caudovirales	Siphoviridae
P050700	medium-quality	metagenome_only	Caudovirales	Siphoviridae
P050991	medium-quality	metagenome_only	Caudovirales	Siphoviridae
P051093	medium-quality	metagenome_only	Caudovirales	Siphoviridae
P051449	low-quality	metagenome_only	Caudovirales	Myoviridae
P051468	medium-quality	both	Caudovirales	Siphoviridae
P051509	high-quality	metagenome_only	Caudovirales	Myoviridae
P051669	low-quality	metagenome_only	Caudovirales	Siphoviridae
P052072	low-quality	metagenome_only	Caudovirales	Unassigned
P052149	high-quality	metagenome_only	Caudovirales	Podoviridae
P052741	low-quality	metagenome_only	Caudovirales	Siphoviridae
P052842	low-quality	metagenome_only	Caudovirales	Siphoviridae
P052894	low-quality	metagenome_only	Caudovirales	Siphoviridae
P053121	low-quality	metagenome_only	Caudovirales	Unassigned
P053343	low-quality	metagenome_only	Caudovirales	Myoviridae
P053406	low-quality	metagenome_only	Caudovirales	Siphoviridae
P053466	medium-quality	both	Caudovirales	Siphoviridae
P053540	low-quality	metagenome_only	Caudovirales	Myoviridae
P053584	high-quality	both	Caudovirales	Myoviridae
P053697	low-quality	both	not classified	not classified
P054026	low-quality	metagenome_only	Caudovirales	Siphoviridae
P054031	low-quality	metagenome_only	Caudovirales	Siphoviridae
P054069	low-quality	metagenome_only	Caudovirales	Unassigned

P054162	low-quality	metagenome_only	Caudovirales	Siphoviridae
P054242	low-quality	metagenome_only	Caudovirales	Myoviridae
P054543	low-quality	metagenome_only	Caudovirales	Myoviridae
P054545	low-quality	metagenome_only	Caudovirales	Unassigned
P055304	medium-quality	metagenome_only	Caudovirales	Siphoviridae
P055406	medium-quality	both	Caudovirales	Myoviridae
P055700	medium-quality	both	Caudovirales	Myoviridae
P056050	low-quality	both	Caudovirales	Unassigned
P056201	not-determined	metagenome_only	Caudovirales	Siphoviridae
P056437	high-quality	metagenome_only	Caudovirales	Siphoviridae
P056545	low-quality	metagenome_only	Caudovirales	Siphoviridae
P056603	high-quality	both	Caudovirales	Siphoviridae
P057128	low-quality	metagenome_only	Caudovirales	Siphoviridae
P057758	low-quality	metagenome_only	Caudovirales	Unassigned
P057929	low-quality	metagenome_only	Caudovirales	Myoviridae
P058010	low-quality	metagenome_only	Caudovirales	Siphoviridae
P058017	low-quality	metagenome_only	Caudovirales	Siphoviridae
P058147	low-quality	metagenome_only	Caudovirales	Siphoviridae
P058199	low-quality	metagenome_only	Caudovirales	Siphoviridae
P058206	low-quality	metagenome_only	Caudovirales	Myoviridae
P058207	low-quality	metagenome_only	Caudovirales	Siphoviridae
P058233	low-quality	metagenome_only	Caudovirales	Unassigned
P058851	low-quality	metagenome_only	Caudovirales	Siphoviridae
P059034	low-quality	metagenome_only	Caudovirales	Siphoviridae
P060044	low-quality	metagenome_only	Caudovirales	Siphoviridae
P060905	low-quality	metagenome_only	Caudovirales	Siphoviridae
P061540	high-quality	metagenome_only	Caudovirales	Myoviridae
P061852	high-quality	both	Caudovirales	Unassigned
P061891	low-quality	metagenome_only	Caudovirales	Siphoviridae
P061970	low-quality	metagenome_only	Caudovirales	Siphoviridae
P062048	low-quality	metagenome_only	Caudovirales	Siphoviridae
P062246	low-quality	both	Caudovirales	Siphoviridae
P062287	low-quality	metagenome_only	Caudovirales	Siphoviridae
P062453	high-quality	both	Caudovirales	Siphoviridae
P062616	low-quality	metagenome_only	Caudovirales	Myoviridae
P062674	low-quality	metagenome_only	Caudovirales	Siphoviridae
P062923	low-quality	metagenome_only	Caudovirales	Siphoviridae
P063509	low-quality	metagenome_only	Caudovirales	Siphoviridae
P063679	low-quality	metagenome_only	Caudovirales	Unassigned
P063802	high-quality	metagenome_only	Caudovirales	Siphoviridae
P063818	low-quality	metagenome_only	Caudovirales	Siphoviridae
P063930	low-quality	metagenome_only	Caudovirales	Siphoviridae
P063933	medium-quality	both	Caudovirales	Siphoviridae
P064284	medium-quality	metagenome_only	Caudovirales	Siphoviridae
P064291	low-quality	metagenome_only	Caudovirales	Siphoviridae
P064765	low-quality	metagenome_only	Caudovirales	Siphoviridae
P064995	medium-quality	metagenome_only	Caudovirales	Siphoviridae

P066264	high-quality	both	Caudovirales	Siphoviridae
P066521	low-quality	metagenome_only	Caudovirales	Siphoviridae
P067229	low-quality	metagenome_only	Caudovirales	Siphoviridae
P067239	medium-quality	metagenome_only	Caudovirales	Siphoviridae
P067377	low-quality	metagenome_only	Caudovirales	Unassigned
P067498	medium-quality	metagenome_only	Caudovirales	Siphoviridae
P067712	low-quality	metagenome_only	Caudovirales	Siphoviridae
P068008	low-quality	metagenome_only	Caudovirales	Siphoviridae
P068130	medium-quality	both	Caudovirales	Siphoviridae
P068431	medium-quality	both	Caudovirales	Myoviridae
P068662	low-quality	metagenome_only	Caudovirales	Siphoviridae
P068786	medium-quality	metagenome_only	Caudovirales	Siphoviridae
P068822	low-quality	metagenome_only	Caudovirales	Myoviridae
P069079	low-quality	metagenome_only	Caudovirales	Myoviridae
P069125	high-quality	both	Caudovirales	Siphoviridae
P069318	medium-quality	metagenome_only	Caudovirales	Siphoviridae
P069408	medium-quality	both	Caudovirales	Siphoviridae
P069943	low-quality	metagenome_only	Caudovirales	Myoviridae
P070186	low-quality	metagenome_only	Caudovirales	Siphoviridae
P070522	low-quality	both	Caudovirales	Siphoviridae
P070821	low-quality	metagenome_only	Caudovirales	Siphoviridae
P071091	high-quality	metagenome_only	Caudovirales	Siphoviridae
P071300	low-quality	metagenome_only	Caudovirales	Myoviridae
P072135	low-quality	metagenome_only	Caudovirales	Siphoviridae
P072213	low-quality	metagenome_only	Caudovirales	Siphoviridae
P073036	high-quality	metagenome_only	Caudovirales	Myoviridae
P073345	low-quality	metagenome_only	Caudovirales	Siphoviridae
P073701	low-quality	metagenome_only	Caudovirales	Myoviridae
P073778	low-quality	metagenome_only	Caudovirales	Unassigned
P074303	low-quality	metagenome_only	Caudovirales	Siphoviridae
P074632	low-quality	metagenome_only	Caudovirales	Siphoviridae
P074661	low-quality	metagenome_only	Caudovirales	Siphoviridae
P074969	low-quality	metagenome_only	Caudovirales	Siphoviridae
P075119	low-quality	metagenome_only	Caudovirales	Siphoviridae
P075262	low-quality	metagenome_only	Caudovirales	Siphoviridae
P075968	low-quality	metagenome_only	Caudovirales	not classified
P076060	medium-quality	both	Caudovirales	Siphoviridae
P076350	complete	metagenome_only	Caudovirales	Myoviridae
P076599	low-quality	metagenome_only	Caudovirales	Unassigned
P077059	low-quality	metagenome_only	Caudovirales	Unassigned
P077684	high-quality	both	Caudovirales	Siphoviridae
P078298	high-quality	both	Caudovirales	Siphoviridae
P078653	medium-quality	metagenome_only	Caudovirales	Siphoviridae
P078728	low-quality	metagenome_only	Caudovirales	Unassigned
P078833	low-quality	both	Caudovirales	Siphoviridae
P079612	low-quality	metagenome_only	Caudovirales	Siphoviridae
P079628	low-quality	metagenome_only	Caudovirales	Siphoviridae

P079652	low-quality	metagenome_only	Caudovirales	Unassigned
P079919	low-quality	metagenome_only	Caudovirales	Myoviridae
P079922	low-quality	metagenome_only	Caudovirales	Siphoviridae
P080013	low-quality	metagenome_only	Caudovirales	Myoviridae
P080128	low-quality	both	Caudovirales	Unassigned
P080308	high-quality	both	Caudovirales	Siphoviridae
P080542	high-quality	both	Caudovirales	Siphoviridae
P080684	low-quality	metagenome_only	Caudovirales	Siphoviridae
P080872	low-quality	metagenome_only	Caudovirales	Siphoviridae
P080909	high-quality	both	Caudovirales	Siphoviridae
P081479	low-quality	metagenome_only	Caudovirales	Siphoviridae
P082073	low-quality	metagenome_only	Caudovirales	Myoviridae
P082151	medium-quality	metagenome_only	Caudovirales	Siphoviridae
P082307	low-quality	metagenome_only	Caudovirales	Siphoviridae
P082313	low-quality	metagenome_only	Caudovirales	Siphoviridae
P082839	low-quality	metagenome_only	Caudovirales	Siphoviridae
P082928	high-quality	both	Caudovirales	Siphoviridae
P083190	low-quality	metagenome_only	Caudovirales	Siphoviridae
P083285	medium-quality	both	Caudovirales	Siphoviridae
P083456	high-quality	both	Caudovirales	Siphoviridae
P083589	high-quality	both	Caudovirales	Unassigned
P083614	low-quality	metagenome_only	Caudovirales	Siphoviridae
P084114	high-quality	both	Caudovirales	Siphoviridae
P084123	high-quality	both	Caudovirales	Siphoviridae
P084132	low-quality	metagenome_only	Caudovirales	Siphoviridae
P084977	low-quality	metagenome_only	Caudovirales	Siphoviridae
P085885	low-quality	metagenome_only	not classified	not classified
P086591	low-quality	metagenome_only	Caudovirales	Siphoviridae
P086654	medium-quality	both	Caudovirales	Siphoviridae
P086906	low-quality	metagenome_only	Caudovirales	Unassigned
P086995	low-quality	metagenome_only	Caudovirales	Unassigned
P087430	low-quality	metagenome_only	Caudovirales	Unassigned
P088396	low-quality	metagenome_only	Caudovirales	Siphoviridae
P088527	medium-quality	both	Caudovirales	Siphoviridae
P088858	low-quality	metagenome_only	Caudovirales	Siphoviridae
P089004	low-quality	metagenome_only	Caudovirales	Siphoviridae
P089026	medium-quality	metagenome_only	Caudovirales	Siphoviridae
P089585	complete	metagenome_only	Caudovirales	Siphoviridae
P089600	low-quality	both	Caudovirales	Siphoviridae
P090522	medium-quality	metagenome_only	Caudovirales	Myoviridae
P090735	high-quality	metagenome_only	Caudovirales	Siphoviridae
P090901	low-quality	metagenome_only	Caudovirales	Siphoviridae
P090907	high-quality	both	Caudovirales	Siphoviridae
P091508	medium-quality	metagenome_only	Caudovirales	Siphoviridae
P091588	low-quality	metagenome_only	Caudovirales	Myoviridae
P091870	low-quality	metagenome_only	Caudovirales	Siphoviridae
P091937	low-quality	metagenome_only	Caudovirales	Unassigned

P092011	low-quality	metagenome_only	Caudovirales	Siphoviridae
P092438	high-quality	metagenome_only	Caudovirales	Siphoviridae
P092946	medium-quality	metagenome_only	Caudovirales	Siphoviridae
P093298	high-quality	both	Caudovirales	Siphoviridae
P093299	low-quality	metagenome_only	Caudovirales	Siphoviridae
P093438	low-quality	metagenome_only	Caudovirales	Podoviridae
P093872	medium-quality	metagenome_only	Caudovirales	Myoviridae
P093943	medium-quality	metagenome_only	Caudovirales	Siphoviridae
P093983	low-quality	metagenome_only	Caudovirales	Myoviridae
P094053	medium-quality	metagenome_only	Caudovirales	Siphoviridae
P094119	low-quality	metagenome_only	Caudovirales	Myoviridae
P094150	low-quality	metagenome_only	Caudovirales	Siphoviridae
P094237	low-quality	metagenome_only	Caudovirales	Siphoviridae
P094712	low-quality	metagenome_only	Caudovirales	Siphoviridae
P094943	medium-quality	both	Caudovirales	Siphoviridae
P095012	medium-quality	metagenome_only	Caudovirales	Siphoviridae
P095056	low-quality	metagenome_only	Caudovirales	Siphoviridae
P095505	medium-quality	metagenome_only	Caudovirales	Siphoviridae
P095703	low-quality	metagenome_only	Caudovirales	Myoviridae
P095749	low-quality	metagenome_only	Caudovirales	Unassigned
P095763	low-quality	metagenome_only	Caudovirales	Myoviridae
P096188	low-quality	metagenome_only	Caudovirales	Unassigned
P096471	low-quality	metagenome_only	Caudovirales	Unassigned
P096642	low-quality	metagenome_only	Caudovirales	Siphoviridae
P096817	medium-quality	metagenome_only	Caudovirales	Myoviridae
P096881	medium-quality	metagenome_only	Caudovirales	Siphoviridae
P097176	low-quality	metagenome_only	Caudovirales	Myoviridae
P097357	high-quality	both	Caudovirales	Siphoviridae
P097816	complete	both	Caudovirales	Siphoviridae
P098451	low-quality	metagenome_only	Caudovirales	Siphoviridae
P098629	medium-quality	metagenome_only	Caudovirales	Siphoviridae
P098656	complete	metagenome_only	Tubulavirales	Inoviridae
P098687	medium-quality	metagenome_only	Caudovirales	Siphoviridae
P098789	low-quality	both	Caudovirales	Siphoviridae
P098903	low-quality	metagenome_only	Caudovirales	Siphoviridae
P099339	high-quality	both	Petitvirales	Microviridae
P099651	medium-quality	metagenome_only	Caudovirales	Siphoviridae
P100157	low-quality	metagenome_only	Caudovirales	Myoviridae
P100347	medium-quality	metagenome_only	Caudovirales	Siphoviridae
P100620	low-quality	metagenome_only	Caudovirales	Siphoviridae
P100689	high-quality	both	Caudovirales	Siphoviridae
P100710	low-quality	metagenome_only	Caudovirales	not classified
P100736	high-quality	metagenome_only	Caudovirales	Siphoviridae
P100784	medium-quality	both	Caudovirales	Siphoviridae
P100877	low-quality	metagenome_only	Caudovirales	Siphoviridae
P100943	high-quality	both	Caudovirales	Siphoviridae
P101113	high-quality	both	Caudovirales	Siphoviridae

P101116	low-quality	both	Caudovirales	Siphoviridae
P101373	low-quality	metagenome_only	Caudovirales	Unassigned
P102042	low-quality	metagenome_only	Caudovirales	Siphoviridae
P102199	complete	metagenome_only	Caudovirales	Siphoviridae
P102265	high-quality	metagenome_only	Caudovirales	Siphoviridae
P102303	medium-quality	both	Caudovirales	Siphoviridae
P102357	high-quality	metagenome_only	Caudovirales	Siphoviridae
P102756	low-quality	metagenome_only	Caudovirales	Siphoviridae
P102800	low-quality	metagenome_only	Caudovirales	Siphoviridae
P102943	low-quality	metagenome_only	Caudovirales	Siphoviridae
P103054	high-quality	metagenome_only	Caudovirales	Siphoviridae
P103271	high-quality	metagenome_only	Caudovirales	Siphoviridae
P104210	low-quality	metagenome_only	Caudovirales	Siphoviridae
P104228	low-quality	metagenome_only	not classified	not classified
P104330	high-quality	metagenome_only	Caudovirales	Siphoviridae
P104530	high-quality	metagenome_only	Caudovirales	Siphoviridae
P104557	low-quality	metagenome_only	Caudovirales	Siphoviridae
P104906	high-quality	both	Caudovirales	Unassigned
P105222	complete	both	Petitvirales	Microviridae
P105432	low-quality	metagenome_only	Caudovirales	Myoviridae
P105582	low-quality	metagenome_only	Caudovirales	Siphoviridae
P105796	medium-quality	metagenome_only	Caudovirales	Siphoviridae
P105898	high-quality	metagenome_only	Caudovirales	Siphoviridae
P106099	low-quality	metagenome_only	Caudovirales	Unassigned
P106309	low-quality	both	Caudovirales	Siphoviridae
P106465	low-quality	metagenome_only	Caudovirales	Siphoviridae
P106626	medium-quality	metagenome_only	Caudovirales	Siphoviridae
P107118	low-quality	metagenome_only	Caudovirales	Podoviridae
P107219	high-quality	metagenome_only	Caudovirales	Siphoviridae
P107797	high-quality	metagenome_only	Caudovirales	Siphoviridae
P108112	low-quality	both	Caudovirales	Siphoviridae
P108264	medium-quality	both	Caudovirales	Siphoviridae
P108299	low-quality	metagenome_only	Caudovirales	Unassigned
P108865	complete	both	Caudovirales	Siphoviridae
P109158	low-quality	metagenome_only	Caudovirales	Siphoviridae
P109283	complete	both	Caudovirales	Myoviridae
P109551	low-quality	metagenome_only	Caudovirales	Siphoviridae
P109917	medium-quality	metagenome_only	Caudovirales	Siphoviridae
P110168	low-quality	metagenome_only	Caudovirales	Siphoviridae
P110315	complete	both	Caudovirales	Siphoviridae
P111556	high-quality	both	Caudovirales	Siphoviridae
P111588	low-quality	metagenome_only	Caudovirales	Siphoviridae
P111624	low-quality	metagenome_only	Caudovirales	Myoviridae
P111732	low-quality	metagenome_only	Caudovirales	Siphoviridae
P112088	low-quality	metagenome_only	not classified	not classified
P112148	low-quality	metagenome_only	Caudovirales	Unassigned
P112297	low-quality	metagenome_only	not classified	not classified

P112607	medium-quality	metagenome_only	Caudovirales	Myoviridae
P112851	high-quality	metagenome_only	Caudovirales	Podoviridae
P113177	medium-quality	metagenome_only	Caudovirales	Siphoviridae
P113279	high-quality	both	Caudovirales	Siphoviridae
V000022	low-quality	virome_only	not classified	not classified
V000031	low-quality	virome_only	Caudovirales	Myoviridae
V000034	low-quality	virome_only	Caudovirales	Siphoviridae
V000050	high-quality	virome_only	Caudovirales	Siphoviridae
V000066	medium-quality	virome_only	Cirlivirales	Circoviridae
V000080	medium-quality	virome_only	Caudovirales	Siphoviridae
V000111	high-quality	virome_only	Cirlivirales	Circoviridae
V000143	medium-quality	virome_only	Petitvirales	Microviridae
V000150	high-quality	virome_only	Caudovirales	Siphoviridae
V000220	low-quality	virome_only	Caudovirales	Siphoviridae
V000224	high-quality	both	Caudovirales	Siphoviridae
V000233	high-quality	virome_only	Cirlivirales	Circoviridae
V000237	medium-quality	virome_only	not classified	not classified
V000252	low-quality	virome_only	Caudovirales	Unassigned
V000253	low-quality	virome_only	Caudovirales	Siphoviridae
V000256	low-quality	both	Caudovirales	Unassigned
V000262	high-quality	virome_only	Petitvirales	Microviridae
V000278	low-quality	virome_only	Caudovirales	Siphoviridae
V000291	complete	virome_only	not classified	not classified
V000300	high-quality	virome_only	not classified	not classified
V000331	high-quality	virome_only	Cirlivirales	Circoviridae
V000332	high-quality	virome_only	Cirlivirales	Circoviridae
V000354	high-quality	virome_only	Caudovirales	Siphoviridae
V000357	medium-quality	virome_only	Caudovirales	Siphoviridae
V000376	medium-quality	both	Caudovirales	Unassigned
V000398	high-quality	virome_only	Caudovirales	Siphoviridae
V000423	low-quality	virome_only	Caudovirales	Siphoviridae
V000448	medium-quality	virome_only	not classified	not classified
V000449	complete	virome_only	Caudovirales	Siphoviridae
V000472	high-quality	virome_only	not classified	not classified
V000492	complete	virome_only	not classified	not classified
V000497	complete	virome_only	Cirlivirales	Circoviridae
V000511	high-quality	virome_only	Caudovirales	Siphoviridae
V000517	not-determined	virome_only	Caudovirales	Unassigned
V000556	low-quality	virome_only	Caudovirales	Siphoviridae
V000576	low-quality	virome_only	Caudovirales	Myoviridae
V000580	high-quality	virome_only	Caudovirales	Siphoviridae
V000583	low-quality	virome_only	Caudovirales	Siphoviridae
V000603	medium-quality	virome_only	Caudovirales	Siphoviridae
V000628	low-quality	virome_only	Caudovirales	Myoviridae
V000659	low-quality	virome_only	not classified	not classified
V000683	medium-quality	virome_only	not classified	not classified
V000693	high-quality	virome_only	Cirlivirales	Circoviridae

V000716	complete	virome_only	Caudovirales	Siphoviridae
V000723	high-quality	virome_only	Caudovirales	Siphoviridae
V000730	complete	virome_only	not classified	not classified
V000734	low-quality	virome_only	Caudovirales	Myoviridae
V000751	complete	virome_only	Cirlivirales	Circoviridae
V000807	complete	virome_only	not classified	not classified
V000852	high-quality	virome_only	Cirlivirales	Circoviridae
V000859	complete	virome_only	not classified	not classified
V000863	high-quality	virome_only	Cirlivirales	Circoviridae
V000881	complete	virome_only	Cirlivirales	Circoviridae
V000896	high-quality	virome_only	not classified	not classified
V000908	high-quality	virome_only	Cirlivirales	Circoviridae
V000918	complete	virome_only	Caudovirales	Siphoviridae
V000934	low-quality	virome_only	Caudovirales	Siphoviridae
V000936	high-quality	virome_only	Cirlivirales	Circoviridae
V000957	complete	virome_only	not classified	not classified
V000969	medium-quality	virome_only	Petitvirales	Microviridae
V000974	high-quality	virome_only	Cirlivirales	Circoviridae
V000978	medium-quality	virome_only	Cirlivirales	Circoviridae
V000979	high-quality	virome_only	Cirlivirales	Circoviridae
V001019	complete	virome_only	not classified	not classified
V001041	low-quality	virome_only	Caudovirales	Siphoviridae
V001045	medium-quality	virome_only	not classified	not classified
V001073	medium-quality	virome_only	Petitvirales	Microviridae
V001097	high-quality	virome_only	Cirlivirales	Circoviridae
V001161	not-determined	virome_only	Caudovirales	Siphoviridae
V001204	complete	virome_only	Caudovirales	Siphoviridae
V001218	medium-quality	virome_only	Caudovirales	Siphoviridae
V001224	high-quality	virome_only	Caudovirales	Siphoviridae
V001239	high-quality	virome_only	Caudovirales	Siphoviridae
V001250	low-quality	virome_only	Caudovirales	Unassigned
V001266	low-quality	virome_only	Caudovirales	Myoviridae
V001276	not-determined	virome_only	Caudovirales	Siphoviridae
V001300	low-quality	virome_only	Caudovirales	Siphoviridae
V001304	medium-quality	virome_only	Caudovirales	Siphoviridae
V001315	complete	virome_only	Cirlivirales	Circoviridae
V001330	low-quality	virome_only	Caudovirales	Siphoviridae
V001358	medium-quality	virome_only	not classified	not classified
V001378	high-quality	virome_only	Cirlivirales	Circoviridae
V001379	high-quality	virome_only	Caudovirales	Siphoviridae
V001384	medium-quality	virome_only	Caudovirales	Siphoviridae
V001413	high-quality	virome_only	Geplafuvirales	Geminiviridae
V001429	low-quality	virome_only	Caudovirales	Unassigned
V001431	medium-quality	virome_only	Petitvirales	Microviridae
V001461	medium-quality	virome_only	Caudovirales	Siphoviridae
V001601	low-quality	virome_only	Caudovirales	Siphoviridae
V001611	low-quality	virome_only	Caudovirales	Siphoviridae

V001616	medium-quality	virome_only	Caudovirales	Siphoviridae
V001620	high-quality	virome_only	Cirlivirales	Circoviridae
V001623	high-quality	virome_only	Cirlivirales	Circoviridae
V001627	complete	both	Caudovirales	Siphoviridae
V001629	complete	virome_only	Cirlivirales	Circoviridae
V001669	low-quality	virome_only	Caudovirales	Siphoviridae
V001695	medium-quality	virome_only	not classified	not classified
V001717	complete	virome_only	no_order_Cruciviridae	Cruciviridae
V001725	high-quality	virome_only	Petitvirales	Microviridae
V001726	medium-quality	virome_only	Cirlivirales	Circoviridae
V001760	high-quality	virome_only	Cirlivirales	Circoviridae
V001765	low-quality	virome_only	Caudovirales	Siphoviridae
V001788	medium-quality	virome_only	Caudovirales	Siphoviridae
V001793	high-quality	virome_only	not classified	not classified
V001810	high-quality	virome_only	Cirlivirales	Circoviridae
V001827	low-quality	virome_only	Caudovirales	Siphoviridae
V001836	low-quality	virome_only	Caudovirales	Myoviridae
V001852	low-quality	virome_only	Caudovirales	Siphoviridae
V001853	low-quality	virome_only	Caudovirales	Unassigned
V001888	high-quality	virome_only	Cirlivirales	Circoviridae
V001924	low-quality	virome_only	not classified	not classified
V001954	medium-quality	virome_only	not classified	not classified
V001965	medium-quality	virome_only	not classified	not classified
V001989	low-quality	virome_only	Caudovirales	Siphoviridae
V001990	low-quality	virome_only	Caudovirales	Siphoviridae
V002010	high-quality	virome_only	Geplafuvirales	Geminiviridae
V002034	complete	virome_only	Caudovirales	Siphoviridae
V002043	low-quality	virome_only	Caudovirales	Siphoviridae
V002073	low-quality	virome_only	Caudovirales	Unassigned
V002081	low-quality	virome_only	Caudovirales	Siphoviridae
V002104	low-quality	virome_only	Caudovirales	Unassigned
V002124	medium-quality	virome_only	Petitvirales	Microviridae
V002126	complete	virome_only	not classified	not classified
V002172	high-quality	virome_only	not classified	not classified
V002173	high-quality	both	Caudovirales	Siphoviridae
V002213	high-quality	virome_only	Cirlivirales	Circoviridae
V002216	low-quality	virome_only	Caudovirales	Siphoviridae
V002217	low-quality	virome_only	Caudovirales	Siphoviridae
V002234	low-quality	virome_only	Caudovirales	Siphoviridae
V002272	high-quality	virome_only	not classified	not classified
V002290	not-determined	virome_only	not classified	not classified
V002333	low-quality	virome_only	Caudovirales	Siphoviridae
V002348	high-quality	virome_only	Caudovirales	Siphoviridae
V002367	low-quality	virome_only	Caudovirales	Siphoviridae
V002410	complete	virome_only	Caudovirales	Siphoviridae
V002443	complete	virome_only	not classified	not classified
V002467	high-quality	virome_only	Caudovirales	Siphoviridae

V002468	high-quality	virome_only	Cirlivirales	Circoviridae
V002506	medium-quality	virome_only	Caudovirales	Siphoviridae
V002528	complete	virome_only	Caudovirales	Siphoviridae
V002552	medium-quality	virome_only	no_order_Cruciviridae	Cruciviridae
V002566	medium-quality	virome_only	Caudovirales	Siphoviridae
V002574	medium-quality	virome_only	Cirlivirales	Circoviridae
V002579	low-quality	virome_only	Caudovirales	Siphoviridae
V002580	low-quality	virome_only	Caudovirales	Siphoviridae
V002594	high-quality	virome_only	Petitvirales	Microviridae
V002614	low-quality	virome_only	Caudovirales	Siphoviridae
V002618	medium-quality	virome_only	not classified	not classified
V002632	low-quality	virome_only	Caudovirales	Unassigned
V002638	complete	virome_only	Caudovirales	Siphoviridae
V002643	complete	virome_only	Petitvirales	Microviridae
V002647	high-quality	virome_only	not classified	not classified
V002692	low-quality	virome_only	Caudovirales	Siphoviridae
V002720	medium-quality	virome_only	not classified	not classified
V002773	complete	virome_only	Petitvirales	Microviridae
V002792	complete	virome_only	not classified	not classified
V002839	complete	virome_only	Caudovirales	Myoviridae
V002850	low-quality	virome_only	Caudovirales	Siphoviridae
V002871	complete	virome_only	Caudovirales	Myoviridae
V002874	complete	virome_only	Petitvirales	Microviridae
V002902	complete	virome_only	Cirlivirales	Circoviridae
V002915	low-quality	virome_only	Caudovirales	Siphoviridae
V002917	complete	virome_only	Caudovirales	Siphoviridae
V002938	complete	virome_only	not classified	not classified
V002960	low-quality	virome_only	Caudovirales	Siphoviridae
V002962	complete	virome_only	not classified	not classified
V002977	medium-quality	virome_only	Caudovirales	Siphoviridae
V002978	complete	virome_only	Petitvirales	Microviridae
V002988	complete	virome_only	Petitvirales	Microviridae
V002992	complete	virome_only	not classified	not classified
V003002	medium-quality	virome_only	no_order_Cruciviridae	Cruciviridae
V003020	complete	virome_only	Petitvirales	Microviridae
V003058	complete	virome_only	Petitvirales	Microviridae
V003082	complete	virome_only	Cirlivirales	Circoviridae
V003090	medium-quality	virome_only	Caudovirales	Siphoviridae
V003115	low-quality	virome_only	Caudovirales	Unassigned
V003206	low-quality	virome_only	Caudovirales	Siphoviridae
V003234	medium-quality	virome_only	Petitvirales	Microviridae
V003235	low-quality	virome_only	Caudovirales	Siphoviridae
V003263	high-quality	virome_only	Caudovirales	Siphoviridae
V003287	medium-quality	virome_only	Caudovirales	Siphoviridae
V003296	complete	virome_only	Caudovirales	Siphoviridae
V003321	medium-quality	virome_only	Caudovirales	Siphoviridae
V003327	high-quality	virome_only	Cirlivirales	Circoviridae

V003357	complete	both	Caudovirales	Siphoviridae
V003376	medium-quality	virome_only	not classified	not classified
V003379	high-quality	virome_only	Cirlivirales	Circoviridae
V003387	low-quality	virome_only	Caudovirales	Unassigned
V003408	medium-quality	virome_only	Petitvirales	Microviridae
V003441	high-quality	virome_only	Cirlivirales	Circoviridae
V003448	medium-quality	virome_only	Caudovirales	Siphoviridae
V003458	high-quality	virome_only	Cirlivirales	Circoviridae
V003479	medium-quality	virome_only	Caudovirales	Siphoviridae
V003482	medium-quality	virome_only	Caudovirales	Siphoviridae
V003526	not-determined	virome_only	Caudovirales	Siphoviridae
V003528	low-quality	virome_only	Caudovirales	Unassigned
V003543	low-quality	virome_only	Caudovirales	Myoviridae
V003547	complete	virome_only	Caudovirales	Siphoviridae
V003586	low-quality	virome_only	Caudovirales	Siphoviridae
V003589	medium-quality	virome_only	Caudovirales	Siphoviridae
V003592	low-quality	virome_only	Caudovirales	Siphoviridae
V003595	high-quality	virome_only	Cirlivirales	Circoviridae
V003596	low-quality	virome_only	Caudovirales	Siphoviridae
V003598	low-quality	virome_only	Caudovirales	Myoviridae
V003644	medium-quality	virome_only	Caudovirales	Siphoviridae
V003707	high-quality	virome_only	Cirlivirales	Circoviridae
V003722	medium-quality	virome_only	Caudovirales	Siphoviridae
V003730	complete	virome_only	not classified	not classified
V003780	high-quality	both	Caudovirales	Siphoviridae
V003781	medium-quality	virome_only	Caudovirales	Siphoviridae
V003797	high-quality	virome_only	Caudovirales	Siphoviridae
V003811	high-quality	virome_only	Cirlivirales	Circoviridae
V003821	medium-quality	virome_only	not classified	not classified
V003826	complete	virome_only	not classified	not classified
V003836	low-quality	virome_only	Caudovirales	Siphoviridae
V003855	high-quality	virome_only	Cirlivirales	Circoviridae
V003856	low-quality	virome_only	Caudovirales	Siphoviridae
V003871	not-determined	virome_only	Caudovirales	Siphoviridae
V003905	high-quality	virome_only	Caudovirales	Siphoviridae
V003909	high-quality	virome_only	not classified	not classified
V003924	high-quality	virome_only	Cirlivirales	Circoviridae
V003931	medium-quality	virome_only	Caudovirales	Siphoviridae
V003941	high-quality	virome_only	Caudovirales	Siphoviridae
V003942	low-quality	virome_only	Caudovirales	Myoviridae
V003943	medium-quality	virome_only	Caudovirales	Siphoviridae
V003946	medium-quality	virome_only	Caudovirales	Siphoviridae
V003962	low-quality	virome_only	Caudovirales	Siphoviridae
V003974	high-quality	virome_only	Cirlivirales	Circoviridae
V003985	complete	virome_only	Caudovirales	Siphoviridae
V003986	low-quality	virome_only	Caudovirales	Siphoviridae
V004034	complete	virome_only	no_order_Cruciviridae	Cruciviridae

V004037	high-quality	virome_only	Cirlivirales	Circoviridae
V004041	high-quality	virome_only	Cirlivirales	Circoviridae
V004074	high-quality	virome_only	Caudovirales	Siphoviridae
V004081	medium-quality	virome_only	not classified	not classified
V004089	high-quality	virome_only	Cirlivirales	Circoviridae
V004134	not-determined	virome_only	Caudovirales	Myoviridae
V004163	high-quality	virome_only	Petitvirales	Microviridae
V004170	low-quality	virome_only	Caudovirales	Siphoviridae
V004183	low-quality	virome_only	Caudovirales	Siphoviridae
V004226	high-quality	virome_only	Caudovirales	Siphoviridae
V004237	complete	virome_only	Caudovirales	Siphoviridae
V004254	complete	virome_only	Petitvirales	Microviridae
V004258	low-quality	virome_only	Caudovirales	Siphoviridae
V004260	low-quality	virome_only	Caudovirales	Siphoviridae
V004280	low-quality	virome_only	Caudovirales	Siphoviridae
V004297	medium-quality	virome_only	Cirlivirales	Circoviridae
V004322	high-quality	virome_only	Caudovirales	Siphoviridae
V004332	low-quality	virome_only	Caudovirales	Siphoviridae
V004333	low-quality	virome_only	Caudovirales	Siphoviridae
V004349	medium-quality	virome_only	Cirlivirales	Circoviridae
V004384	high-quality	virome_only	Cirlivirales	Circoviridae
V004405	low-quality	virome_only	Caudovirales	Siphoviridae
V004424	medium-quality	virome_only	Caudovirales	Siphoviridae
V004433	low-quality	virome_only	Caudovirales	Siphoviridae
V004444	complete	virome_only	Caudovirales	Siphoviridae
V004467	high-quality	virome_only	Cirlivirales	Circoviridae
V004498	low-quality	virome_only	Caudovirales	Siphoviridae
V004540	low-quality	virome_only	Caudovirales	Siphoviridae
V004555	high-quality	virome_only	not classified	not classified
V004563	high-quality	virome_only	Caudovirales	Siphoviridae
V004584	complete	virome_only	Cirlivirales	Circoviridae
V004605	low-quality	both	Caudovirales	Unassigned
V004623	medium-quality	virome_only	not classified	not classified
V004634	high-quality	virome_only	not classified	not classified
V004669	high-quality	virome_only	Caudovirales	Siphoviridae
V004715	complete	virome_only	not classified	not classified
V004730	low-quality	virome_only	Caudovirales	Siphoviridae
V004776	complete	virome_only	Cirlivirales	Circoviridae
V004803	high-quality	virome_only	Cirlivirales	Circoviridae
V004857	low-quality	virome_only	Caudovirales	Unassigned
V004868	high-quality	virome_only	Caudovirales	Siphoviridae
V004876	high-quality	virome_only	Caudovirales	Siphoviridae
V004898	low-quality	virome_only	Caudovirales	Myoviridae
V004903	high-quality	virome_only	Cirlivirales	Circoviridae
V004918	low-quality	virome_only	Caudovirales	Siphoviridae
V004935	low-quality	virome_only	Caudovirales	Siphoviridae
V004981	complete	virome_only	Cirlivirales	Circoviridae

V004983	medium-quality	virome_only	Cirlivirales	Circoviridae
V005027	medium-quality	virome_only	not classified	not classified
V005077	not-determined	virome_only	Caudovirales	Siphoviridae
V005078	low-quality	virome_only	Caudovirales	Siphoviridae
V005079	low-quality	virome_only	Caudovirales	Siphoviridae
V005099	medium-quality	virome_only	Caudovirales	Siphoviridae
V005114	low-quality	virome_only	Caudovirales	Siphoviridae
V005115	high-quality	virome_only	Caudovirales	Siphoviridae
V005137	low-quality	virome_only	Caudovirales	Siphoviridae
V005141	high-quality	virome_only	Cirlivirales	Circoviridae
V005164	high-quality	virome_only	Petitvirales	Microviridae
V005167	high-quality	virome_only	Cirlivirales	Circoviridae
V005169	high-quality	virome_only	Caudovirales	Siphoviridae
V005181	low-quality	virome_only	Caudovirales	Unassigned
V005190	medium-quality	virome_only	Caudovirales	Unassigned
V005193	low-quality	virome_only	Caudovirales	Siphoviridae
V005226	not-determined	virome_only	Caudovirales	Unassigned
V005249	complete	virome_only	Caudovirales	Siphoviridae
V005259	low-quality	virome_only	Caudovirales	Siphoviridae
V005267	low-quality	virome_only	Caudovirales	Siphoviridae
V005269	low-quality	virome_only	Caudovirales	Myoviridae
V005274	high-quality	virome_only	Cirlivirales	Circoviridae
V005279	complete	virome_only	Cirlivirales	Circoviridae
V005283	low-quality	virome_only	Caudovirales	Unassigned
V005284	complete	both	Caudovirales	Siphoviridae
V005318	low-quality	virome_only	Caudovirales	Siphoviridae
V005343	medium-quality	virome_only	Caudovirales	Siphoviridae
V005348	low-quality	virome_only	Caudovirales	Siphoviridae
V005349	medium-quality	virome_only	not classified	not classified
V005354	low-quality	virome_only	Caudovirales	Siphoviridae
V005361	high-quality	virome_only	Caudovirales	Siphoviridae
V005371	low-quality	virome_only	Caudovirales	Siphoviridae
V005375	not-determined	virome_only	Caudovirales	Siphoviridae
V005377	low-quality	virome_only	Caudovirales	Siphoviridae
V005380	high-quality	virome_only	Petitvirales	Microviridae
V005397	low-quality	virome_only	Caudovirales	Siphoviridae
V005399	high-quality	virome_only	Caudovirales	Siphoviridae
V005405	low-quality	virome_only	Caudovirales	Siphoviridae
V005416	low-quality	virome_only	Caudovirales	Siphoviridae
V005421	medium-quality	virome_only	Caudovirales	Siphoviridae
V005435	medium-quality	virome_only	Caudovirales	Siphoviridae
V005460	low-quality	virome_only	Caudovirales	Siphoviridae
V005483	complete	both	Caudovirales	Siphoviridae
V005497	high-quality	virome_only	not classified	not classified
V005500	medium-quality	virome_only	not classified	not classified
V005507	medium-quality	virome_only	Caudovirales	Myoviridae
V005537	high-quality	virome_only	Cirlivirales	Circoviridae

V005547	complete	virome_only	Caudovirales	Siphoviridae
V005573	medium-quality	virome_only	Caudovirales	Siphoviridae
V005582	complete	virome_only	Caudovirales	Siphoviridae
V005597	medium-quality	virome_only	Caudovirales	Siphoviridae
V005605	low-quality	virome_only	Caudovirales	Unassigned
V005623	high-quality	virome_only	Cirlivirales	Circoviridae
V005630	high-quality	virome_only	Caudovirales	Siphoviridae
V005631	medium-quality	virome_only	not classified	not classified
V005709	not-determined	virome_only	Caudovirales	Myoviridae
V005723	low-quality	virome_only	Caudovirales	Siphoviridae
V005725	medium-quality	virome_only	not classified	not classified
V005738	complete	virome_only	not classified	not classified
V005743	low-quality	virome_only	Caudovirales	Unassigned
V005746	medium-quality	virome_only	Caudovirales	Siphoviridae
V005747	low-quality	both	Caudovirales	Siphoviridae
V005752	not-determined	virome_only	Caudovirales	Siphoviridae
V005757	low-quality	virome_only	Caudovirales	Siphoviridae
V005789	complete	virome_only	Caudovirales	Siphoviridae
V005790	low-quality	virome_only	Caudovirales	Unassigned
V005799	high-quality	virome_only	Caudovirales	Siphoviridae
V005802	complete	virome_only	Caudovirales	Siphoviridae
V005814	complete	virome_only	Caudovirales	Siphoviridae
V005839	high-quality	virome_only	Caudovirales	Siphoviridae
V005845	complete	virome_only	not classified	not classified
V005866	medium-quality	virome_only	Caudovirales	Siphoviridae
V005871	complete	virome_only	Caudovirales	Myoviridae
V005873	medium-quality	virome_only	Caudovirales	Siphoviridae
V005877	low-quality	virome_only	Caudovirales	Siphoviridae
V005880	complete	virome_only	not classified	not classified
V005887	low-quality	virome_only	Caudovirales	Unassigned
V005907	low-quality	both	Caudovirales	Siphoviridae
V005984	not-determined	virome_only	Caudovirales	Siphoviridae
V005991	low-quality	virome_only	Caudovirales	Unassigned
V006007	low-quality	virome_only	Caudovirales	Siphoviridae
V006010	high-quality	virome_only	Caudovirales	Siphoviridae
V006012	high-quality	virome_only	Caudovirales	Unassigned
V006014	low-quality	virome_only	Caudovirales	Siphoviridae
V006017	high-quality	both	Caudovirales	Siphoviridae
V006028	not-determined	virome_only	Caudovirales	Myoviridae
V006032	complete	both	Caudovirales	Siphoviridae
V006048	low-quality	virome_only	not classified	not classified
V006051	low-quality	virome_only	Caudovirales	Myoviridae
V006091	low-quality	virome_only	Caudovirales	Myoviridae
V006093	complete	virome_only	Caudovirales	Siphoviridae
V006120	high-quality	virome_only	Cirlivirales	Circoviridae
V006123	high-quality	virome_only	Cirlivirales	Circoviridae
V006125	complete	virome_only	Caudovirales	Siphoviridae

V006190	high-quality	virome_only	Caudovirales	Myoviridae
V006212	complete	virome_only	Caudovirales	Siphoviridae
V006215	complete	virome_only	Cirlivirales	Circoviridae
V006228	low-quality	virome_only	Caudovirales	Siphoviridae
V006239	high-quality	virome_only	Cirlivirales	Circoviridae
V006240	medium-quality	virome_only	Caudovirales	Siphoviridae
V006247	low-quality	virome_only	Caudovirales	Myoviridae
V006250	low-quality	virome_only	Caudovirales	Siphoviridae
V006265	medium-quality	virome_only	Caudovirales	Siphoviridae
V006271	low-quality	virome_only	Caudovirales	Siphoviridae
V006273	high-quality	virome_only	Petitvirales	Microviridae
V006278	low-quality	virome_only	Caudovirales	Siphoviridae
V006280	low-quality	virome_only	Caudovirales	Unassigned
V006297	complete	virome_only	Petitvirales	Microviridae
V006306	complete	virome_only	Cirlivirales	Circoviridae
V006308	complete	virome_only	Cirlivirales	Circoviridae
V006314	complete	virome_only	Cirlivirales	Circoviridae
V006315	high-quality	virome_only	Cirlivirales	Circoviridae
V006325	high-quality	virome_only	Petitvirales	Microviridae
V006337	complete	virome_only	Cirlivirales	Circoviridae
V006339	complete	virome_only	not classified	not classified
V006364	medium-quality	virome_only	Caudovirales	Siphoviridae
V006380	complete	virome_only	Petitvirales	Microviridae
V006396	complete	virome_only	Cirlivirales	Circoviridae
V006407	complete	virome_only	Petitvirales	Microviridae
V006408	complete	virome_only	Caudovirales	Siphoviridae
V006411	low-quality	virome_only	Caudovirales	Siphoviridae
V006419	low-quality	virome_only	Caudovirales	Siphoviridae
V006444	complete	virome_only	not classified	not classified
V006458	high-quality	virome_only	Petitvirales	Microviridae
V006467	medium-quality	virome_only	Caudovirales	Siphoviridae
V006469	medium-quality	virome_only	not classified	not classified
V006483	complete	virome_only	Caudovirales	Siphoviridae
V006485	medium-quality	virome_only	Caudovirales	Siphoviridae
V006501	complete	virome_only	Petitvirales	Microviridae
V006506	complete	virome_only	Cirlivirales	Circoviridae
V006575	low-quality	virome_only	Caudovirales	Siphoviridae
V006665	not-determined	virome_only	Caudovirales	Siphoviridae
V006691	complete	virome_only	Caudovirales	Podoviridae
V006704	low-quality	virome_only	Caudovirales	Siphoviridae
V006709	low-quality	virome_only	Caudovirales	Myoviridae
V006732	low-quality	virome_only	Petitvirales	Microviridae
V006749	low-quality	virome_only	Caudovirales	Siphoviridae
V006763	low-quality	virome_only	Caudovirales	Siphoviridae
V006769	complete	virome_only	Caudovirales	Siphoviridae
V006790	low-quality	virome_only	Caudovirales	Unassigned
V006792	high-quality	virome_only	Caudovirales	Siphoviridae

V006822	high-quality	both	Caudovirales	Siphoviridae
V006824	high-quality	virome_only	no_order_Anelloviridae	Anelloviridae
V006867	low-quality	virome_only	Caudovirales	Unassigned
V006938	low-quality	virome_only	Caudovirales	Siphoviridae
V006952	medium-quality	virome_only	Caudovirales	Unassigned
V006960	high-quality	both	Caudovirales	Siphoviridae
V006971	medium-quality	virome_only	Caudovirales	Siphoviridae
V006984	low-quality	virome_only	not classified	not classified
V007049	not-determined	virome_only	Caudovirales	Podoviridae
V007078	complete	virome_only	Caudovirales	Siphoviridae
V007095	low-quality	virome_only	Caudovirales	Myoviridae
V007120	not-determined	virome_only	Caudovirales	Siphoviridae
V007201	low-quality	virome_only	Caudovirales	Unassigned
V007241	high-quality	both	Caudovirales	Siphoviridae
V007242	medium-quality	virome_only	Caudovirales	Siphoviridae
V007246	high-quality	virome_only	not classified	not classified
V007252	low-quality	virome_only	Caudovirales	Microviridae
V007256	high-quality	both	Caudovirales	Siphoviridae
V007281	low-quality	virome_only	Caudovirales	Siphoviridae
V007300	high-quality	both	Caudovirales	Myoviridae
V007343	medium-quality	virome_only	Caudovirales	Siphoviridae
V007356	low-quality	virome_only	Caudovirales	Unassigned
V007374	complete	virome_only	Petitvirales	Microviridae
V007465	low-quality	virome_only	Caudovirales	Siphoviridae
V007549	medium-quality	both	Caudovirales	Unassigned
V007551	not-determined	virome_only	Caudovirales	Siphoviridae
V007552	low-quality	virome_only	Caudovirales	Siphoviridae
V007566	complete	virome_only	Caudovirales	Siphoviridae
V007576	low-quality	virome_only	Caudovirales	Siphoviridae
V007611	low-quality	virome_only	Caudovirales	Siphoviridae
V007621	high-quality	virome_only	Caudovirales	Siphoviridae
V007633	low-quality	virome_only	Caudovirales	Siphoviridae
V007658	low-quality	virome_only	Caudovirales	Unassigned
V007676	high-quality	both	Caudovirales	Siphoviridae
V007678	high-quality	virome_only	Caudovirales	Unassigned
V007703	high-quality	virome_only	not classified	not classified
V007710	low-quality	virome_only	Caudovirales	Unassigned
V007724	low-quality	virome_only	Caudovirales	Siphoviridae
V007749	low-quality	virome_only	Caudovirales	Unassigned
V007758	low-quality	virome_only	Caudovirales	Siphoviridae
V007811	high-quality	virome_only	not classified	not classified
V007832	medium-quality	virome_only	Caudovirales	Myoviridae
V007898	medium-quality	virome_only	Cirlivirales	Circoviridae
V007914	low-quality	virome_only	Caudovirales	Siphoviridae
V007919	low-quality	virome_only	Caudovirales	Siphoviridae
V007923	low-quality	virome_only	Caudovirales	Siphoviridae
V007942	low-quality	virome_only	Caudovirales	Unassigned

V007943	complete	virome_only	Caudovirales	Siphoviridae
V007959	low-quality	virome_only	Caudovirales	Siphoviridae
V007964	low-quality	virome_only	Caudovirales	Siphoviridae
V008001	high-quality	virome_only	no_order_Cruciviridae	Cruciviridae
V008015	high-quality	virome_only	Mulpavirales	Nanoviridae
V008020	low-quality	virome_only	Caudovirales	Siphoviridae
V008024	low-quality	virome_only	Caudovirales	Siphoviridae
V008033	high-quality	virome_only	Caudovirales	Siphoviridae
V008049	low-quality	virome_only	Caudovirales	Unassigned
V008054	low-quality	virome_only	Caudovirales	Unassigned
V008056	complete	both	Caudovirales	Podoviridae
V008105	low-quality	virome_only	Caudovirales	Siphoviridae
V008116	medium-quality	virome_only	Caudovirales	Siphoviridae
V008128	high-quality	both	Caudovirales	Siphoviridae
V008140	low-quality	virome_only	Caudovirales	Myoviridae
V008152	medium-quality	virome_only	Cirlivirales	Circoviridae
V008200	low-quality	virome_only	Caudovirales	Myoviridae
V008229	low-quality	virome_only	Caudovirales	Siphoviridae
V008272	high-quality	virome_only	Caudovirales	Siphoviridae
V008305	medium-quality	virome_only	Caudovirales	Myoviridae
V008330	complete	virome_only	Caudovirales	Siphoviridae
V008353	low-quality	virome_only	Caudovirales	Siphoviridae
V008378	medium-quality	virome_only	Caudovirales	Podoviridae
V008389	high-quality	virome_only	Caudovirales	Siphoviridae
V008399	low-quality	virome_only	Caudovirales	Unassigned
V008465	medium-quality	virome_only	Caudovirales	Siphoviridae
V008467	not-determined	virome_only	Caudovirales	Siphoviridae
V008485	low-quality	virome_only	Caudovirales	Siphoviridae
V008492	high-quality	both	Caudovirales	Myoviridae
V008500	low-quality	virome_only	Caudovirales	Siphoviridae
V008503	medium-quality	virome_only	Caudovirales	Myoviridae
V008504	high-quality	virome_only	Caudovirales	Siphoviridae
V008508	medium-quality	both	Caudovirales	Unassigned
V008532	complete	virome_only	Caudovirales	Siphoviridae
V008543	low-quality	virome_only	Caudovirales	Podoviridae
V008555	medium-quality	virome_only	Caudovirales	Podoviridae
V008563	low-quality	virome_only	Caudovirales	Siphoviridae
V008574	high-quality	virome_only	Caudovirales	Siphoviridae
V008579	medium-quality	virome_only	Caudovirales	Unassigned
V008607	low-quality	virome_only	Caudovirales	Siphoviridae
V008617	high-quality	virome_only	Caudovirales	Myoviridae
V008621	medium-quality	virome_only	Caudovirales	Siphoviridae
V008703	low-quality	virome_only	Caudovirales	Siphoviridae
V008723	not-determined	virome_only	Caudovirales	Unassigned
V008742	high-quality	both	Caudovirales	Myoviridae
V008749	low-quality	virome_only	Caudovirales	Siphoviridae
V008787	high-quality	virome_only	Caudovirales	Siphoviridae

V008821	complete	virome_only	Caudovirales	Siphoviridae
V008826	low-quality	virome_only	Caudovirales	Siphoviridae
V008850	low-quality	virome_only	Caudovirales	Siphoviridae
V008865	high-quality	virome_only	Caudovirales	Siphoviridae
V008946	low-quality	virome_only	Caudovirales	Siphoviridae
V008949	not-determined	virome_only	Caudovirales	Siphoviridae
V008952	medium-quality	virome_only	Caudovirales	Siphoviridae
V008968	high-quality	virome_only	Caudovirales	Siphoviridae
V008978	low-quality	virome_only	Caudovirales	Siphoviridae
V008996	low-quality	virome_only	Caudovirales	Siphoviridae
V008998	low-quality	virome_only	Caudovirales	Siphoviridae
V009031	low-quality	virome_only	Caudovirales	Siphoviridae
V009062	medium-quality	virome_only	Caudovirales	Siphoviridae
V009082	low-quality	virome_only	Caudovirales	Myoviridae
V009119	not-determined	virome_only	Caudovirales	Siphoviridae
V009139	medium-quality	virome_only	Caudovirales	Siphoviridae
V009151	high-quality	virome_only	Caudovirales	Siphoviridae
V009169	not-determined	virome_only	Caudovirales	Siphoviridae
V009174	low-quality	virome_only	not classified	not classified
V009178	medium-quality	both	Caudovirales	Myoviridae
V009199	low-quality	virome_only	Caudovirales	Unassigned
V009203	high-quality	virome_only	Caudovirales	Siphoviridae
V009231	not-determined	virome_only	Caudovirales	Podoviridae
V009246	low-quality	virome_only	Caudovirales	Myoviridae
V009258	low-quality	virome_only	Caudovirales	Siphoviridae
V009259	low-quality	virome_only	Caudovirales	Podoviridae
V009276	low-quality	virome_only	Caudovirales	Siphoviridae
V009292	low-quality	virome_only	Caudovirales	Siphoviridae
V009293	high-quality	virome_only	Cirlivirales	Circoviridae
V009313	low-quality	virome_only	Caudovirales	Siphoviridae
V009316	low-quality	virome_only	Caudovirales	Unassigned
V009320	high-quality	virome_only	Caudovirales	Siphoviridae
V009332	low-quality	virome_only	Caudovirales	Unassigned
V009338	low-quality	virome_only	Caudovirales	Unassigned
V009343	low-quality	virome_only	Caudovirales	Siphoviridae
V009349	low-quality	virome_only	Caudovirales	Myoviridae
V009354	low-quality	virome_only	Caudovirales	Siphoviridae
V009380	high-quality	virome_only	Petitvirales	Microviridae
V009460	low-quality	virome_only	Caudovirales	Siphoviridae
V009503	not-determined	virome_only	Caudovirales	Unassigned
V009523	low-quality	virome_only	Caudovirales	Unassigned
V009540	low-quality	virome_only	Caudovirales	Siphoviridae
V009561	low-quality	virome_only	Caudovirales	Siphoviridae
V009570	low-quality	virome_only	Caudovirales	Unassigned
V009617	high-quality	virome_only	Caudovirales	Siphoviridae
V009644	low-quality	virome_only	Caudovirales	Siphoviridae
V009649	high-quality	both	Caudovirales	Siphoviridae

V009653	low-quality	virome_only	Caudovirales	Siphoviridae
V009678	low-quality	virome_only	Caudovirales	Myoviridae
V009694	low-quality	virome_only	Caudovirales	Podoviridae
V009698	low-quality	virome_only	Caudovirales	Siphoviridae
V009700	high-quality	virome_only	Caudovirales	Podoviridae
V009752	medium-quality	both	Caudovirales	Siphoviridae
V009813	low-quality	virome_only	Caudovirales	Siphoviridae
V009867	low-quality	virome_only	Caudovirales	Myoviridae
V009971	high-quality	both	Caudovirales	Siphoviridae
V009995	medium-quality	virome_only	Caudovirales	Myoviridae
V010001	medium-quality	both	Caudovirales	Siphoviridae
V010007	low-quality	virome_only	Caudovirales	Siphoviridae
V010022	medium-quality	both	Caudovirales	Siphoviridae
V010064	high-quality	virome_only	Caudovirales	Siphoviridae
V010117	medium-quality	virome_only	Caudovirales	Siphoviridae
V010126	high-quality	virome_only	Caudovirales	Myoviridae
V010129	low-quality	virome_only	Caudovirales	Siphoviridae
V010148	low-quality	virome_only	Caudovirales	Unassigned
V010176	low-quality	virome_only	Caudovirales	Unassigned
V010237	low-quality	virome_only	not classified	not classified
V010241	complete	virome_only	Caudovirales	Siphoviridae
V010247	low-quality	virome_only	Caudovirales	Myoviridae
V010251	low-quality	virome_only	Caudovirales	Myoviridae
V010276	low-quality	virome_only	Caudovirales	Siphoviridae
V010289	low-quality	virome_only	Caudovirales	Siphoviridae
V010314	high-quality	both	Caudovirales	Siphoviridae
V010315	low-quality	virome_only	Caudovirales	Siphoviridae
V010316	complete	virome_only	Caudovirales	Siphoviridae
V010336	medium-quality	virome_only	Caudovirales	Siphoviridae
V010387	low-quality	virome_only	Caudovirales	Siphoviridae
V010398	low-quality	virome_only	Caudovirales	Siphoviridae
V010401	low-quality	virome_only	Caudovirales	Siphoviridae
V010406	complete	virome_only	Caudovirales	Siphoviridae
V010407	medium-quality	both	Caudovirales	Siphoviridae
V010417	low-quality	virome_only	Caudovirales	Siphoviridae
V010423	low-quality	both	Caudovirales	Siphoviridae
V010466	medium-quality	virome_only	Caudovirales	Unassigned
V010497	medium-quality	virome_only	Caudovirales	Myoviridae
V010519	medium-quality	virome_only	not classified	not classified
V010579	low-quality	virome_only	Caudovirales	Myoviridae
V010646	complete	virome_only	Caudovirales	Siphoviridae
V010738	high-quality	virome_only	Caudovirales	Siphoviridae
V010761	low-quality	virome_only	Caudovirales	Siphoviridae
V010764	low-quality	virome_only	Caudovirales	Siphoviridae
V010786	low-quality	virome_only	Caudovirales	Unassigned
V010787	low-quality	virome_only	Caudovirales	Siphoviridae
V010807	medium-quality	virome_only	Caudovirales	Podoviridae

V010828	complete	virome_only	Caudovirales	Siphoviridae
V010858	low-quality	virome_only	Caudovirales	Siphoviridae
V010861	low-quality	virome_only	Caudovirales	Siphoviridae
V010883	low-quality	virome_only	Caudovirales	Siphoviridae
V010891	low-quality	virome_only	Caudovirales	Siphoviridae
V010916	low-quality	virome_only	Caudovirales	Myoviridae
V010933	medium-quality	both	Caudovirales	Siphoviridae
V010968	complete	virome_only	Caudovirales	Siphoviridae
V010973	low-quality	virome_only	Caudovirales	Siphoviridae
V010979	complete	virome_only	not classified	not classified
V010980	not-determined	virome_only	Caudovirales	Siphoviridae
V010984	low-quality	virome_only	Caudovirales	Siphoviridae
V011012	low-quality	virome_only	Caudovirales	Siphoviridae
V011030	complete	virome_only	not classified	not classified
V011056	low-quality	virome_only	Caudovirales	Siphoviridae
V011058	low-quality	virome_only	Caudovirales	Unassigned
V011087	low-quality	virome_only	Caudovirales	Siphoviridae
V011190	low-quality	virome_only	Caudovirales	Siphoviridae
V011204	high-quality	virome_only	Caudovirales	Siphoviridae
V011207	medium-quality	virome_only	Caudovirales	Siphoviridae
V011216	low-quality	virome_only	Caudovirales	Myoviridae
V011240	medium-quality	virome_only	Caudovirales	Siphoviridae
V011245	medium-quality	virome_only	Caudovirales	Siphoviridae
V011249	complete	virome_only	Caudovirales	Siphoviridae
V011277	medium-quality	virome_only	Caudovirales	Myoviridae
V011356	complete	virome_only	Cirlivirales	Circoviridae
V011368	low-quality	virome_only	Caudovirales	Siphoviridae
V011375	not-determined	virome_only	Caudovirales	Siphoviridae
V011395	not-determined	virome_only	Caudovirales	not classified
V011441	low-quality	virome_only	Caudovirales	Siphoviridae
V011454	low-quality	virome_only	Caudovirales	Siphoviridae
V011459	medium-quality	virome_only	Caudovirales	Siphoviridae
V011461	low-quality	virome_only	Caudovirales	Myoviridae
V011462	medium-quality	virome_only	Caudovirales	Siphoviridae
V011517	low-quality	virome_only	Caudovirales	Siphoviridae
V011533	low-quality	virome_only	Caudovirales	Siphoviridae
V011573	low-quality	virome_only	Caudovirales	Siphoviridae
V011578	medium-quality	virome_only	Caudovirales	Siphoviridae
V011634	low-quality	virome_only	Caudovirales	Siphoviridae
V011639	medium-quality	virome_only	Caudovirales	Siphoviridae
V011651	low-quality	virome_only	Caudovirales	Siphoviridae
V011694	low-quality	virome_only	Caudovirales	Siphoviridae
V011724	not-determined	virome_only	Caudovirales	Siphoviridae
V011734	complete	virome_only	Caudovirales	Siphoviridae
V011735	complete	both	Caudovirales	Siphoviridae
V011741	medium-quality	virome_only	Caudovirales	Siphoviridae
V011742	low-quality	virome_only	Caudovirales	Siphoviridae

V011819	complete	virome_only	Cirlivirales	Circoviridae
V011860	not-determined	virome_only	Caudovirales	Siphoviridae
V011873	low-quality	virome_only	Caudovirales	Siphoviridae
V011884	low-quality	virome_only	Caudovirales	Siphoviridae
V011929	medium-quality	virome_only	Caudovirales	Siphoviridae
V011943	high-quality	both	Caudovirales	Siphoviridae
V011996	complete	virome_only	not classified	not classified
V012034	low-quality	virome_only	Caudovirales	Siphoviridae
V012043	low-quality	virome_only	Caudovirales	Siphoviridae
V012049	low-quality	both	Caudovirales	Siphoviridae
V012068	complete	virome_only	not classified	not classified
V012085	complete	virome_only	Petitvirales	Microviridae
V012101	medium-quality	both	Caudovirales	Siphoviridae
V012107	low-quality	virome_only	Caudovirales	Siphoviridae
V012148	medium-quality	virome_only	not classified	not classified
V012153	low-quality	virome_only	Caudovirales	Siphoviridae
V012172	low-quality	virome_only	Caudovirales	Siphoviridae
V012232	complete	virome_only	Caudovirales	Siphoviridae
V012237	low-quality	virome_only	Caudovirales	Siphoviridae
V012240	complete	virome_only	Caudovirales	Siphoviridae
V012245	medium-quality	virome_only	Caudovirales	Siphoviridae
V012260	high-quality	virome_only	Caudovirales	Siphoviridae
V012262	low-quality	virome_only	Caudovirales	Podoviridae
V012292	complete	virome_only	not classified	not classified
V012311	low-quality	both	Caudovirales	Myoviridae
V012341	complete	virome_only	Cirlivirales	Circoviridae
V012357	low-quality	virome_only	Caudovirales	Siphoviridae
V012376	low-quality	both	Caudovirales	Siphoviridae
V012386	high-quality	both	Caudovirales	Siphoviridae
V012430	complete	virome_only	not classified	not classified
V012451	low-quality	virome_only	Caudovirales	Siphoviridae
V012470	not-determined	virome_only	Caudovirales	Siphoviridae
V012471	not-determined	virome_only	Caudovirales	Siphoviridae
V012658	not-determined	virome_only	Caudovirales	Siphoviridae
V012717	low-quality	virome_only	Caudovirales	Siphoviridae
V012748	low-quality	both	Caudovirales	Siphoviridae
V012806	high-quality	virome_only	Caudovirales	Siphoviridae
V012814	low-quality	virome_only	Caudovirales	Siphoviridae
V012834	low-quality	virome_only	Caudovirales	Siphoviridae
V012943	low-quality	virome_only	Caudovirales	Siphoviridae
V012977	low-quality	virome_only	Caudovirales	Siphoviridae
V013001	not-determined	virome_only	Caudovirales	Siphoviridae
V013073	low-quality	virome_only	Caudovirales	Siphoviridae
V013123	low-quality	virome_only	Caudovirales	Siphoviridae
V013234	low-quality	virome_only	Caudovirales	Siphoviridae
V013235	low-quality	virome_only	Caudovirales	Siphoviridae
V013251	medium-quality	both	Caudovirales	Siphoviridae

V013259	low-quality	virome_only	Caudovirales	Siphoviridae
V013261	medium-quality	virome_only	Caudovirales	Siphoviridae
V013285	not-determined	virome_only	Caudovirales	Siphoviridae
V013314	low-quality	virome_only	Caudovirales	Myoviridae
V013339	low-quality	virome_only	Caudovirales	Myoviridae
V013390	low-quality	virome_only	Caudovirales	Siphoviridae
V013494	low-quality	virome_only	Caudovirales	Siphoviridae
V013501	low-quality	virome_only	Caudovirales	Unassigned
V013517	low-quality	virome_only	Caudovirales	Unassigned
V013541	low-quality	virome_only	Caudovirales	Siphoviridae
V013616	low-quality	virome_only	Caudovirales	Siphoviridae
V013645	medium-quality	both	Caudovirales	Myoviridae
V013679	low-quality	virome_only	Caudovirales	Siphoviridae
V013682	medium-quality	virome_only	Caudovirales	Siphoviridae
V013791	low-quality	virome_only	Caudovirales	Siphoviridae
V013891	medium-quality	virome_only	Caudovirales	Siphoviridae
V013965	low-quality	virome_only	Caudovirales	Siphoviridae
V013979	low-quality	virome_only	Caudovirales	Siphoviridae
V014037	low-quality	virome_only	Caudovirales	Siphoviridae
V014054	low-quality	virome_only	Caudovirales	Myoviridae
V014089	medium-quality	virome_only	Caudovirales	Siphoviridae
V014120	medium-quality	virome_only	Caudovirales	Myoviridae
V014139	low-quality	virome_only	Caudovirales	Siphoviridae
V014175	low-quality	virome_only	Caudovirales	Myoviridae
V014264	complete	both	Caudovirales	Podoviridae
V014322	low-quality	both	Caudovirales	Podoviridae
V014379	high-quality	both	Caudovirales	Siphoviridae
V014391	high-quality	virome_only	Caudovirales	Myoviridae
V014424	low-quality	both	Caudovirales	Siphoviridae
V014524	low-quality	virome_only	Caudovirales	Siphoviridae
V014565	low-quality	virome_only	Caudovirales	Myoviridae
V014584	low-quality	virome_only	not classified	not classified
V014634	low-quality	virome_only	Caudovirales	Siphoviridae
V014681	complete	both	Caudovirales	Siphoviridae
V014695	high-quality	both	Caudovirales	Siphoviridae
V014709	low-quality	virome_only	Caudovirales	Siphoviridae
V014836	low-quality	virome_only	Caudovirales	Siphoviridae
V015041	medium-quality	virome_only	Caudovirales	Siphoviridae
V015055	low-quality	virome_only	Caudovirales	Unassigned
V015073	low-quality	virome_only	Caudovirales	Siphoviridae
V015078	low-quality	virome_only	Caudovirales	Siphoviridae
V015099	low-quality	virome_only	Caudovirales	Siphoviridae
V015142	high-quality	virome_only	Caudovirales	Siphoviridae
V015144	low-quality	virome_only	Caudovirales	Siphoviridae
V015221	not-determined	virome_only	not classified	not classified
V015223	low-quality	virome_only	Caudovirales	Unassigned
V015231	low-quality	virome_only	not classified	not classified

V015235	high-quality	both	Caudovirales	Siphoviridae
V015299	high-quality	virome_only	Caudovirales	Siphoviridae
V015372	medium-quality	both	Caudovirales	Siphoviridae
V015379	high-quality	both	Caudovirales	Siphoviridae
V015454	low-quality	virome_only	Caudovirales	Siphoviridae
V015479	low-quality	virome_only	Caudovirales	Podoviridae
V015545	low-quality	virome_only	Caudovirales	Siphoviridae
V015579	low-quality	virome_only	Caudovirales	Siphoviridae
V015620	low-quality	virome_only	Caudovirales	Unassigned
V015630	low-quality	virome_only	Caudovirales	Siphoviridae
V015686	low-quality	virome_only	Caudovirales	Podoviridae
V015734	complete	both	Caudovirales	Siphoviridae
V015775	high-quality	both	Caudovirales	Siphoviridae
V015894	low-quality	virome_only	Caudovirales	Unassigned
V015895	low-quality	both	Caudovirales	Siphoviridae
V015925	low-quality	virome_only	Caudovirales	Myoviridae
V015934	low-quality	virome_only	Caudovirales	Unassigned
V015935	low-quality	virome_only	Caudovirales	Siphoviridae
V015949	low-quality	virome_only	Caudovirales	Siphoviridae
V015989	low-quality	virome_only	Caudovirales	Siphoviridae
V016011	low-quality	virome_only	Caudovirales	Siphoviridae
V016192	low-quality	both	Caudovirales	Siphoviridae
V016203	low-quality	both	Caudovirales	Myoviridae
V016240	low-quality	virome_only	Caudovirales	Unassigned
V016279	low-quality	virome_only	Caudovirales	Siphoviridae
V016372	low-quality	virome_only	Caudovirales	Siphoviridae
V016381	low-quality	virome_only	Caudovirales	Siphoviridae
V016464	low-quality	virome_only	Caudovirales	Myoviridae
V016587	low-quality	virome_only	Caudovirales	Siphoviridae
V016641	low-quality	virome_only	Caudovirales	Siphoviridae
V016707	high-quality	virome_only	Caudovirales	Siphoviridae
V016744	complete	both	Caudovirales	Siphoviridae
V016760	complete	virome_only	Caudovirales	Siphoviridae
V016780	low-quality	virome_only	Caudovirales	Unassigned
V016811	not-determined	virome_only	not classified	not classified
V016844	not-determined	virome_only	Caudovirales	Siphoviridae
V016847	medium-quality	virome_only	Caudovirales	Myoviridae
V016883	low-quality	virome_only	Caudovirales	Siphoviridae
V016904	complete	both	Caudovirales	Podoviridae
V016919	medium-quality	both	Caudovirales	Siphoviridae
V016956	complete	both	Caudovirales	Siphoviridae
V017052	low-quality	virome_only	Caudovirales	Siphoviridae
V017164	low-quality	virome_only	Caudovirales	Siphoviridae
V017239	low-quality	virome_only	Caudovirales	Siphoviridae
V017246	high-quality	virome_only	Caudovirales	Siphoviridae
V017387	low-quality	virome_only	Caudovirales	Siphoviridae
V017389	low-quality	virome_only	Caudovirales	Siphoviridae

V017394	medium-quality	virome_only	Caudovirales	Podoviridae
V017399	low-quality	virome_only	Caudovirales	Siphoviridae
V017574	low-quality	virome_only	Caudovirales	Siphoviridae
V017603	medium-quality	virome_only	Caudovirales	Siphoviridae
V017639	low-quality	virome_only	Caudovirales	Siphoviridae
V017646	low-quality	virome_only	Caudovirales	Siphoviridae
V017679	low-quality	virome_only	Caudovirales	Unassigned
V017700	high-quality	both	Caudovirales	Siphoviridae
V017757	low-quality	virome_only	Caudovirales	Siphoviridae
V017813	low-quality	virome_only	Caudovirales	Siphoviridae
V017817	medium-quality	both	Caudovirales	Siphoviridae
V017840	low-quality	virome_only	Caudovirales	Siphoviridae
V017842	low-quality	virome_only	not classified	not classified
V017870	low-quality	virome_only	Caudovirales	Siphoviridae
V017886	high-quality	virome_only	Caudovirales	Siphoviridae
V017888	medium-quality	virome_only	Caudovirales	Siphoviridae
V017894	high-quality	virome_only	Caudovirales	Siphoviridae
V017936	low-quality	virome_only	Caudovirales	Siphoviridae
V017946	high-quality	both	Caudovirales	Siphoviridae
V018032	low-quality	both	Caudovirales	Unassigned
V018037	low-quality	virome_only	Caudovirales	Siphoviridae
V018085	medium-quality	both	Caudovirales	Siphoviridae
V018138	high-quality	virome_only	Caudovirales	Siphoviridae
V018146	medium-quality	virome_only	Caudovirales	Podoviridae
V018213	low-quality	virome_only	Caudovirales	Siphoviridae
V018297	low-quality	virome_only	Caudovirales	Siphoviridae
V018325	low-quality	virome_only	Caudovirales	Siphoviridae
V018388	high-quality	virome_only	Caudovirales	Myoviridae
V018419	low-quality	virome_only	Caudovirales	Siphoviridae
V018567	medium-quality	virome_only	Caudovirales	Siphoviridae
V018580	medium-quality	virome_only	Caudovirales	Siphoviridae
V018625	low-quality	virome_only	Caudovirales	Unassigned
V018664	medium-quality	both	Caudovirales	Myoviridae
V018739	low-quality	virome_only	Caudovirales	Siphoviridae
V018814	low-quality	virome_only	not classified	not classified
V018820	low-quality	virome_only	Caudovirales	Siphoviridae
V018822	medium-quality	both	Caudovirales	Siphoviridae
V018885	medium-quality	virome_only	Caudovirales	Siphoviridae
V018917	high-quality	both	Caudovirales	Siphoviridae
V018941	complete	virome_only	Caudovirales	Siphoviridae
V018943	low-quality	virome_only	Caudovirales	Unassigned
V018983	medium-quality	virome_only	Caudovirales	Siphoviridae
V019046	high-quality	both	Caudovirales	Siphoviridae
V019195	medium-quality	virome_only	Caudovirales	Myoviridae
V019213	low-quality	virome_only	Caudovirales	Siphoviridae
V019236	low-quality	virome_only	Caudovirales	Siphoviridae
V019311	low-quality	virome_only	Caudovirales	Siphoviridae

V019330	low-quality	virome_only	Caudovirales	Siphoviridae
V019386	low-quality	virome_only	Caudovirales	Siphoviridae
V019426	low-quality	virome_only	Caudovirales	Siphoviridae
V019452	low-quality	virome_only	Caudovirales	Siphoviridae
V019456	not-determined	virome_only	Caudovirales	Unassigned
V019499	medium-quality	both	Caudovirales	Siphoviridae
V019515	low-quality	virome_only	Caudovirales	Siphoviridae
V019525	medium-quality	virome_only	Caudovirales	Siphoviridae
V019560	low-quality	virome_only	Caudovirales	Siphoviridae
V019586	low-quality	virome_only	Caudovirales	Siphoviridae
V019592	not-determined	virome_only	Caudovirales	Unassigned
V019635	low-quality	virome_only	Caudovirales	Siphoviridae
V019656	low-quality	virome_only	Caudovirales	Siphoviridae
V019671	complete	virome_only	Caudovirales	Siphoviridae
V019683	medium-quality	virome_only	Caudovirales	Myoviridae
V019686	complete	virome_only	Caudovirales	Siphoviridae
V019707	not-determined	virome_only	Caudovirales	Unassigned
V019738	low-quality	both	Caudovirales	Myoviridae
V019762	low-quality	virome_only	Caudovirales	Siphoviridae
V019763	low-quality	virome_only	Caudovirales	Siphoviridae
V019807	medium-quality	virome_only	Caudovirales	Myoviridae
V019826	low-quality	virome_only	Caudovirales	Unassigned
V019855	high-quality	virome_only	Caudovirales	Unassigned
V019904	low-quality	virome_only	Caudovirales	Siphoviridae
V019964	complete	virome_only	Caudovirales	Siphoviridae
V019974	complete	virome_only	Caudovirales	Siphoviridae
V020117	low-quality	virome_only	Caudovirales	Myoviridae
V020210	complete	both	Caudovirales	Unassigned
V020223	low-quality	virome_only	Caudovirales	Myoviridae
V020230	low-quality	virome_only	Caudovirales	Siphoviridae
V020242	high-quality	both	Caudovirales	Myoviridae
V020286	low-quality	virome_only	Caudovirales	Siphoviridae
V020291	low-quality	virome_only	Caudovirales	Siphoviridae
V020367	medium-quality	both	Caudovirales	Podoviridae
V020405	high-quality	virome_only	Caudovirales	Siphoviridae
V020528	low-quality	virome_only	Caudovirales	Siphoviridae
V020549	low-quality	virome_only	Caudovirales	Unassigned
V020563	high-quality	both	Caudovirales	Unassigned
V020570	low-quality	virome_only	Caudovirales	Siphoviridae
V020627	high-quality	virome_only	Caudovirales	Siphoviridae
V020632	not-determined	virome_only	Caudovirales	Myoviridae
V020700	low-quality	virome_only	Caudovirales	Siphoviridae
V020834	complete	virome_only	Caudovirales	Siphoviridae
V020841	not-determined	virome_only	Caudovirales	Siphoviridae
V020853	not-determined	virome_only	Caudovirales	Siphoviridae
V020873	low-quality	virome_only	Caudovirales	Siphoviridae
V020903	medium-quality	virome_only	Caudovirales	Siphoviridae

V021007	medium-quality	virome_only	Caudovirales	Siphoviridae
V021022	complete	virome_only	Caudovirales	Siphoviridae
V021057	low-quality	virome_only	Caudovirales	Unassigned
V021108	low-quality	virome_only	Caudovirales	Unassigned
V021186	low-quality	virome_only	Caudovirales	Siphoviridae
V021256	low-quality	virome_only	Caudovirales	Siphoviridae
V021270	low-quality	virome_only	Caudovirales	Siphoviridae
V021275	medium-quality	virome_only	Caudovirales	Siphoviridae
V021317	medium-quality	virome_only	Caudovirales	Siphoviridae
V021354	complete	virome_only	Caudovirales	Siphoviridae
V021362	low-quality	virome_only	Caudovirales	Siphoviridae
V021395	low-quality	virome_only	Caudovirales	Siphoviridae
V021398	not-determined	virome_only	Caudovirales	Siphoviridae
V021420	low-quality	virome_only	Caudovirales	Siphoviridae
V021467	low-quality	virome_only	Caudovirales	Siphoviridae
V021525	low-quality	virome_only	Caudovirales	Myoviridae
V021558	low-quality	virome_only	Caudovirales	Siphoviridae
V021645	not-determined	virome_only	not classified	not classified
V021696	high-quality	both	Caudovirales	Siphoviridae
V021785	low-quality	virome_only	Caudovirales	Myoviridae
V021794	high-quality	both	Caudovirales	Siphoviridae
V021845	medium-quality	virome_only	no_order_Phycodnaviridae	Phycodnaviridae
V021864	high-quality	virome_only	Caudovirales	Myoviridae
V022021	low-quality	virome_only	Caudovirales	Siphoviridae
V022144	low-quality	virome_only	Caudovirales	Siphoviridae
V022148	low-quality	virome_only	Caudovirales	Unassigned
V022162	high-quality	virome_only	Caudovirales	Siphoviridae
V022188	low-quality	virome_only	Caudovirales	Siphoviridae
V022190	low-quality	virome_only	Caudovirales	Siphoviridae
V022261	low-quality	virome_only	Caudovirales	Siphoviridae
V022283	medium-quality	virome_only	Caudovirales	Siphoviridae
V022339	low-quality	virome_only	Caudovirales	Unassigned
V022490	low-quality	virome_only	Caudovirales	Siphoviridae
V022551	low-quality	both	Caudovirales	Siphoviridae
V022585	high-quality	virome_only	Caudovirales	Siphoviridae
V022600	medium-quality	virome_only	Caudovirales	Myoviridae
V022700	low-quality	virome_only	Caudovirales	Unassigned
V022886	high-quality	virome_only	Caudovirales	Myoviridae
V022955	low-quality	virome_only	Caudovirales	Unassigned
V022980	low-quality	virome_only	Caudovirales	Siphoviridae
V022989	not-determined	virome_only	Caudovirales	Siphoviridae
V022992	low-quality	virome_only	Caudovirales	Siphoviridae
V023004	not-determined	virome_only	not classified	not classified
V023044	medium-quality	both	Caudovirales	Siphoviridae
V023146	complete	virome_only	Caudovirales	Myoviridae
V023162	not-determined	virome_only	Caudovirales	Siphoviridae
V023321	low-quality	virome_only	Caudovirales	Siphoviridae

V023362	low-quality	virome_only	Caudovirales	Siphoviridae
V023386	complete	virome_only	Caudovirales	Siphoviridae
V023400	low-quality	virome_only	Caudovirales	Siphoviridae
V023408	high-quality	virome_only	Caudovirales	Siphoviridae
V023437	low-quality	virome_only	Caudovirales	Siphoviridae
V023441	medium-quality	virome_only	Caudovirales	Siphoviridae
V023534	low-quality	virome_only	Caudovirales	Siphoviridae
V023559	medium-quality	virome_only	Caudovirales	Siphoviridae
V023630	medium-quality	both	Caudovirales	Siphoviridae
V023670	low-quality	virome_only	Caudovirales	Siphoviridae
V023695	medium-quality	virome_only	Caudovirales	Siphoviridae
V023755	medium-quality	virome_only	Caudovirales	Myoviridae
V023758	low-quality	virome_only	Caudovirales	Siphoviridae
V023829	low-quality	virome_only	Caudovirales	Siphoviridae
V023838	low-quality	virome_only	Caudovirales	Siphoviridae
V023882	low-quality	virome_only	Caudovirales	Siphoviridae
V023910	low-quality	virome_only	Caudovirales	Myoviridae
V023916	medium-quality	virome_only	Caudovirales	Podoviridae
V023937	low-quality	virome_only	Caudovirales	Siphoviridae
V024039	low-quality	virome_only	Caudovirales	Siphoviridae
V024062	high-quality	virome_only	Caudovirales	Siphoviridae
V024155	low-quality	virome_only	Caudovirales	Siphoviridae
V024168	low-quality	virome_only	Caudovirales	Siphoviridae
V024233	complete	both	Caudovirales	Unassigned
V024305	complete	both	Caudovirales	Myoviridae
V024316	high-quality	both	Caudovirales	Siphoviridae
V024323	medium-quality	both	Caudovirales	Myoviridae
V024349	low-quality	virome_only	Caudovirales	Siphoviridae
V024433	low-quality	virome_only	Caudovirales	Siphoviridae
V024449	high-quality	virome_only	Caudovirales	Siphoviridae
V024460	complete	virome_only	Caudovirales	Siphoviridae
V024463	high-quality	both	Caudovirales	Siphoviridae
V024503	low-quality	virome_only	not classified	not classified
V024510	complete	both	Caudovirales	Siphoviridae
V024528	medium-quality	both	Caudovirales	Siphoviridae
V024553	complete	both	Caudovirales	Siphoviridae
V024562	high-quality	virome_only	Caudovirales	Siphoviridae
V024598	complete	both	Caudovirales	Myoviridae
V024616	complete	both	Caudovirales	Myoviridae
V024617	low-quality	virome_only	Caudovirales	Unassigned
V024618	complete	virome_only	not classified	not classified
V024619	complete	both	Caudovirales	Myoviridae
V024621	complete	both	Caudovirales	Siphoviridae
V024622	complete	virome_only	Caudovirales	Siphoviridae
V024626	low-quality	virome_only	Caudovirales	Myoviridae
V024693	low-quality	virome_only	Caudovirales	Podoviridae
V024830	low-quality	virome_only	Caudovirales	Siphoviridae

V024882	low-quality	virome_only	Caudovirales	Siphoviridae
V024938	low-quality	virome_only	Caudovirales	Siphoviridae
V024959	low-quality	virome_only	Caudovirales	Myoviridae
V024977	low-quality	virome_only	Caudovirales	Siphoviridae
V025026	low-quality	virome_only	Caudovirales	Siphoviridae
V025047	not-determined	virome_only	not classified	not classified
V025145	low-quality	virome_only	Caudovirales	Siphoviridae
V025148	low-quality	virome_only	Caudovirales	Podoviridae
V025213	not-determined	virome_only	Caudovirales	Unassigned
V025222	low-quality	virome_only	Caudovirales	Siphoviridae
V025287	low-quality	virome_only	Caudovirales	Siphoviridae
V025353	low-quality	virome_only	Caudovirales	Siphoviridae
V025374	not-determined	virome_only	Caudovirales	Myoviridae
V025391	low-quality	virome_only	Caudovirales	Myoviridae
V025484	high-quality	virome_only	Caudovirales	Siphoviridae
V025582	complete	virome_only	Caudovirales	Siphoviridae
V025636	low-quality	virome_only	Caudovirales	Siphoviridae
V025659	high-quality	virome_only	Caudovirales	Siphoviridae
V025715	low-quality	both	Caudovirales	Siphoviridae
V025739	low-quality	virome_only	Caudovirales	Siphoviridae
V025746	complete	both	Caudovirales	Siphoviridae
V025772	low-quality	virome_only	not classified	not classified
V025830	low-quality	virome_only	Caudovirales	Unassigned
V025849	low-quality	virome_only	Caudovirales	Unassigned
V025857	low-quality	virome_only	Caudovirales	Unassigned
V025872	low-quality	virome_only	Caudovirales	Siphoviridae
V025877	low-quality	both	Caudovirales	Siphoviridae
V025893	low-quality	virome_only	Caudovirales	Siphoviridae
V025978	low-quality	virome_only	Caudovirales	Siphoviridae
V026023	low-quality	virome_only	Caudovirales	Siphoviridae
V026028	low-quality	virome_only	Caudovirales	Unassigned
V026045	low-quality	virome_only	Caudovirales	Siphoviridae
V026167	low-quality	virome_only	Caudovirales	Unassigned
V026197	low-quality	virome_only	Caudovirales	Unassigned
V026277	low-quality	virome_only	Caudovirales	not classified
V026317	low-quality	virome_only	Caudovirales	Siphoviridae
V026567	low-quality	both	Caudovirales	Siphoviridae
V026569	low-quality	virome_only	Caudovirales	Myoviridae
V026572	medium-quality	virome_only	Caudovirales	Siphoviridae
V026580	low-quality	virome_only	not classified	not classified
V026629	low-quality	virome_only	Caudovirales	Unassigned
V026692	high-quality	virome_only	Caudovirales	Siphoviridae
V026721	complete	virome_only	Caudovirales	Siphoviridae
V026854	high-quality	both	Caudovirales	Siphoviridae
V026903	low-quality	virome_only	Caudovirales	Unassigned
V026931	low-quality	virome_only	Caudovirales	Siphoviridae
V026938	not-determined	virome_only	Caudovirales	Unassigned

V026953	low-quality	virome_only	Caudovirales	Myoviridae
V027065	low-quality	virome_only	Caudovirales	Siphoviridae
V027077	low-quality	virome_only	Caudovirales	Siphoviridae
V027191	medium-quality	both	Caudovirales	Siphoviridae
V027262	low-quality	virome_only	Caudovirales	Siphoviridae
V027288	complete	both	Caudovirales	Siphoviridae
V027312	low-quality	virome_only	Caudovirales	Siphoviridae
V027415	low-quality	virome_only	Caudovirales	Unassigned
V027484	not-determined	virome_only	not classified	not classified
V027529	not-determined	virome_only	Caudovirales	Myoviridae
V027564	high-quality	both	Caudovirales	Siphoviridae
V027588	low-quality	both	Caudovirales	Siphoviridae
V027654	low-quality	virome_only	Caudovirales	Siphoviridae
V027681	low-quality	virome_only	Caudovirales	Siphoviridae
V027735	not-determined	virome_only	Caudovirales	Siphoviridae
V027767	high-quality	both	Caudovirales	Siphoviridae
V027807	high-quality	virome_only	Caudovirales	Siphoviridae
V027808	low-quality	virome_only	Caudovirales	Siphoviridae
V027857	low-quality	virome_only	Caudovirales	Siphoviridae
V027932	low-quality	virome_only	Caudovirales	Myoviridae
V028005	high-quality	both	Caudovirales	Siphoviridae
V028032	not-determined	virome_only	Caudovirales	Unassigned
V028082	low-quality	virome_only	Caudovirales	Siphoviridae
V028108	low-quality	virome_only	Caudovirales	Siphoviridae
V028147	low-quality	virome_only	Caudovirales	Siphoviridae
V028168	low-quality	virome_only	Caudovirales	Siphoviridae
V028173	low-quality	virome_only	Caudovirales	Siphoviridae
V028217	not-determined	virome_only	Caudovirales	Unassigned
V028240	medium-quality	virome_only	Caudovirales	Siphoviridae
V028256	medium-quality	virome_only	Caudovirales	Siphoviridae
V028339	low-quality	virome_only	Caudovirales	Siphoviridae
V028363	high-quality	virome_only	Caudovirales	Siphoviridae
V028436	low-quality	virome_only	Caudovirales	Siphoviridae
V028471	medium-quality	virome_only	Caudovirales	Siphoviridae
V028473	complete	virome_only	Caudovirales	Siphoviridae
V028512	complete	both	Caudovirales	Siphoviridae
V028627	low-quality	virome_only	Caudovirales	Siphoviridae
V028659	complete	both	Caudovirales	Siphoviridae
V028771	not-determined	virome_only	Caudovirales	Siphoviridae
V028802	not-determined	virome_only	not classified	not classified
V028804	low-quality	both	Caudovirales	Myoviridae
V028852	low-quality	virome_only	Caudovirales	Siphoviridae
V028871	low-quality	virome_only	Caudovirales	Siphoviridae
V028874	not-determined	virome_only	Caudovirales	Siphoviridae
V028963	complete	both	Caudovirales	Siphoviridae
V028982	low-quality	virome_only	Caudovirales	Myoviridae
V028988	low-quality	virome_only	Caudovirales	Siphoviridae

V029024	low-quality	virome_only	Caudovirales	Siphoviridae
V029058	low-quality	virome_only	Caudovirales	Myoviridae
V029159	low-quality	virome_only	Caudovirales	Siphoviridae
V029169	high-quality	virome_only	Caudovirales	Siphoviridae
V029203	high-quality	virome_only	Caudovirales	Siphoviridae
V029249	low-quality	virome_only	Caudovirales	Siphoviridae
V029256	low-quality	virome_only	Caudovirales	Myoviridae
V029273	low-quality	virome_only	Caudovirales	Siphoviridae
V029274	low-quality	virome_only	Caudovirales	Siphoviridae
V029291	high-quality	both	Caudovirales	Siphoviridae
V029341	low-quality	both	Caudovirales	Siphoviridae
V029371	low-quality	virome_only	Caudovirales	Siphoviridae
V029507	low-quality	virome_only	Caudovirales	Siphoviridae
V029576	low-quality	virome_only	Caudovirales	Siphoviridae
V029636	low-quality	virome_only	Caudovirales	Siphoviridae
V029733	not-determined	virome_only	not classified	not classified
V029743	medium-quality	virome_only	Caudovirales	Siphoviridae
V029775	low-quality	virome_only	Caudovirales	Myoviridae
V029790	low-quality	virome_only	Caudovirales	Myoviridae
V029827	high-quality	virome_only	Caudovirales	Siphoviridae
V029837	low-quality	both	Caudovirales	Unassigned
V029895	low-quality	virome_only	Caudovirales	Unassigned
V029905	low-quality	virome_only	Caudovirales	Siphoviridae
V029953	low-quality	virome_only	Caudovirales	Siphoviridae
V029965	low-quality	virome_only	Caudovirales	Siphoviridae
V029970	low-quality	virome_only	Caudovirales	Siphoviridae
V030113	high-quality	both	Caudovirales	Siphoviridae
V030120	high-quality	both	Caudovirales	Siphoviridae
V030129	medium-quality	virome_only	Caudovirales	Siphoviridae
V030168	low-quality	virome_only	Caudovirales	Siphoviridae
V030239	medium-quality	virome_only	Caudovirales	Siphoviridae
V030260	not-determined	virome_only	Caudovirales	Myoviridae
V030298	low-quality	virome_only	Caudovirales	Siphoviridae
V030332	low-quality	virome_only	Caudovirales	Siphoviridae
V030334	low-quality	virome_only	Caudovirales	Unassigned
V030385	not-determined	virome_only	Caudovirales	Podoviridae
V030519	not-determined	virome_only	Caudovirales	Siphoviridae
V030594	low-quality	virome_only	Caudovirales	Siphoviridae
V030662	low-quality	virome_only	Caudovirales	Myoviridae
V030867	low-quality	virome_only	Caudovirales	Siphoviridae
V030957	low-quality	virome_only	Caudovirales	Myoviridae
V030970	medium-quality	virome_only	Caudovirales	Siphoviridae
V031078	low-quality	virome_only	Caudovirales	Siphoviridae
V031088	medium-quality	virome_only	Caudovirales	Siphoviridae
V031144	low-quality	virome_only	Caudovirales	Siphoviridae
V031183	low-quality	virome_only	Caudovirales	Siphoviridae
V031205	not-determined	virome_only	Caudovirales	Siphoviridae

V031252	low-quality	virome_only	Caudovirales	Siphoviridae
V031263	low-quality	virome_only	Caudovirales	Siphoviridae
V031277	low-quality	virome_only	Caudovirales	Siphoviridae
V031368	low-quality	virome_only	Caudovirales	Siphoviridae
V031380	medium-quality	virome_only	Caudovirales	Siphoviridae
V031392	high-quality	virome_only	Caudovirales	Siphoviridae
V031428	low-quality	virome_only	Caudovirales	Siphoviridae
V031456	low-quality	virome_only	Caudovirales	Myoviridae
V031551	medium-quality	both	Caudovirales	Siphoviridae
V031582	complete	virome_only	Caudovirales	Siphoviridae
V031631	high-quality	both	Caudovirales	Siphoviridae
V031665	low-quality	virome_only	Caudovirales	Unassigned
V031807	low-quality	both	Caudovirales	Myoviridae
V031844	low-quality	virome_only	Caudovirales	Siphoviridae
V031900	medium-quality	virome_only	Caudovirales	Siphoviridae
V031974	low-quality	virome_only	Caudovirales	Siphoviridae
V032069	low-quality	virome_only	Caudovirales	Unassigned
V032087	medium-quality	virome_only	not classified	not classified
V032088	low-quality	virome_only	Caudovirales	Siphoviridae
V032111	medium-quality	both	Caudovirales	Myoviridae
V032124	low-quality	virome_only	Caudovirales	Siphoviridae
V032172	low-quality	virome_only	Caudovirales	Siphoviridae
V032189	low-quality	virome_only	Caudovirales	Siphoviridae
V032217	low-quality	virome_only	Caudovirales	Siphoviridae
V032234	high-quality	virome_only	Caudovirales	Podoviridae
V032303	low-quality	virome_only	Caudovirales	Siphoviridae
V032318	low-quality	virome_only	Caudovirales	Siphoviridae
V032381	low-quality	virome_only	Caudovirales	Siphoviridae
V032397	low-quality	virome_only	Caudovirales	Podoviridae
V032419	low-quality	virome_only	Caudovirales	Siphoviridae
V032499	complete	virome_only	Caudovirales	Siphoviridae
V032520	not-determined	virome_only	not classified	not classified
V032550	low-quality	virome_only	Caudovirales	Siphoviridae
V032647	low-quality	virome_only	Caudovirales	Unassigned
V032773	high-quality	virome_only	Caudovirales	Siphoviridae
V032910	low-quality	virome_only	Caudovirales	Siphoviridae
V032918	complete	both	Caudovirales	Siphoviridae
V033141	complete	virome_only	not classified	not classified
V033159	low-quality	virome_only	Caudovirales	Siphoviridae
V033169	not-determined	virome_only	Caudovirales	Myoviridae
V033214	high-quality	virome_only	Caudovirales	Myoviridae
V033220	low-quality	virome_only	Caudovirales	Siphoviridae
V033258	low-quality	both	Caudovirales	Siphoviridae
V033281	low-quality	virome_only	Caudovirales	Siphoviridae
V033307	low-quality	virome_only	Caudovirales	Podoviridae
V033340	low-quality	virome_only	Caudovirales	Unassigned
V033349	low-quality	virome_only	Caudovirales	Siphoviridae

V033369	low-quality	virome_only	Caudovirales	Myoviridae
V033389	not-determined	virome_only	Caudovirales	Siphoviridae
V033470	low-quality	virome_only	Caudovirales	Unassigned
V033482	low-quality	virome_only	Caudovirales	Siphoviridae
V033541	low-quality	virome_only	Caudovirales	Unassigned
V033586	low-quality	virome_only	Caudovirales	Myoviridae
V033594	low-quality	virome_only	Caudovirales	Myoviridae
V033617	low-quality	virome_only	Caudovirales	Siphoviridae
V033654	low-quality	virome_only	Caudovirales	Siphoviridae
V033655	low-quality	virome_only	Caudovirales	Myoviridae
V033756	not-determined	virome_only	Caudovirales	Siphoviridae
V033847	high-quality	virome_only	Caudovirales	Siphoviridae
V033929	high-quality	virome_only	Caudovirales	Siphoviridae
V033963	medium-quality	virome_only	Caudovirales	Siphoviridae
V033980	low-quality	virome_only	Caudovirales	Myoviridae
V033992	high-quality	both	Caudovirales	Myoviridae
V034179	medium-quality	virome_only	Caudovirales	Siphoviridae
V034180	medium-quality	virome_only	Caudovirales	Siphoviridae
V034226	low-quality	virome_only	Caudovirales	Siphoviridae
V034444	low-quality	virome_only	Caudovirales	Siphoviridae
V034450	low-quality	virome_only	Caudovirales	Siphoviridae
V034485	low-quality	virome_only	Caudovirales	Unassigned
V034504	low-quality	virome_only	Caudovirales	Siphoviridae
V034555	low-quality	virome_only	Caudovirales	Siphoviridae
V034563	medium-quality	virome_only	Caudovirales	Siphoviridae
V034594	low-quality	virome_only	Caudovirales	Unassigned
V034613	high-quality	both	Caudovirales	Myoviridae
V034658	low-quality	virome_only	Caudovirales	Siphoviridae
V034697	low-quality	virome_only	Caudovirales	Myoviridae
V034702	low-quality	virome_only	Caudovirales	Siphoviridae
V034756	low-quality	virome_only	Caudovirales	Siphoviridae
V034803	low-quality	virome_only	Caudovirales	Siphoviridae
V034810	low-quality	virome_only	Caudovirales	Siphoviridae
V034853	high-quality	both	Caudovirales	Siphoviridae
V034930	low-quality	both	Caudovirales	Siphoviridae
V034967	low-quality	virome_only	Caudovirales	Siphoviridae
V034994	low-quality	virome_only	Caudovirales	Unassigned
V035014	low-quality	virome_only	Caudovirales	Unassigned
V035213	low-quality	virome_only	Caudovirales	Siphoviridae
V035293	low-quality	virome_only	Caudovirales	Siphoviridae
V035383	low-quality	virome_only	Caudovirales	Siphoviridae
V035390	low-quality	virome_only	Caudovirales	Siphoviridae
V035486	low-quality	virome_only	Caudovirales	Myoviridae
V035554	complete	virome_only	Petitvirales	Microviridae
V035556	low-quality	virome_only	Caudovirales	Siphoviridae
V035651	complete	both	Caudovirales	Siphoviridae
V035672	high-quality	virome_only	Caudovirales	Siphoviridae

V035677	low-quality	virome_only	Caudovirales	Myoviridae
V035749	low-quality	virome_only	Caudovirales	Siphoviridae
V035785	medium-quality	virome_only	Caudovirales	Unassigned
V035803	low-quality	virome_only	Caudovirales	Podoviridae
V035867	low-quality	virome_only	Caudovirales	Myoviridae
V035886	medium-quality	both	Caudovirales	Myoviridae
V035970	low-quality	virome_only	Caudovirales	Siphoviridae
V036048	complete	virome_only	Caudovirales	Siphoviridae
V036073	low-quality	virome_only	Caudovirales	Siphoviridae
V036101	low-quality	virome_only	Caudovirales	Siphoviridae
V036135	low-quality	virome_only	Caudovirales	Podoviridae
V036169	low-quality	virome_only	Caudovirales	Siphoviridae
V036239	low-quality	virome_only	Caudovirales	Siphoviridae
V036255	low-quality	virome_only	Caudovirales	Siphoviridae
V036323	low-quality	virome_only	Caudovirales	Siphoviridae
V036563	medium-quality	virome_only	Caudovirales	Siphoviridae
V036598	low-quality	virome_only	Caudovirales	Myoviridae
V036616	complete	virome_only	Caudovirales	Siphoviridae
V036634	low-quality	virome_only	Caudovirales	Siphoviridae
V036641	not-determined	virome_only	Caudovirales	Unassigned
V036653	low-quality	virome_only	Caudovirales	Unassigned
V036674	low-quality	virome_only	not classified	not classified
V036897	low-quality	virome_only	Caudovirales	Siphoviridae
V036943	low-quality	virome_only	Caudovirales	Siphoviridae
V036953	low-quality	both	Caudovirales	Myoviridae
V036965	low-quality	virome_only	Caudovirales	Siphoviridae
V037062	medium-quality	virome_only	Petitvirales	Microviridae
V037092	complete	virome_only	Caudovirales	Siphoviridae
V037140	low-quality	virome_only	Caudovirales	Siphoviridae
V037145	low-quality	virome_only	Caudovirales	Siphoviridae
V037149	medium-quality	virome_only	Caudovirales	Siphoviridae
V037173	low-quality	virome_only	Caudovirales	Siphoviridae
V037241	low-quality	virome_only	Caudovirales	Siphoviridae
V037267	low-quality	virome_only	Caudovirales	Siphoviridae
V037294	low-quality	virome_only	Caudovirales	Siphoviridae
V037296	low-quality	virome_only	Caudovirales	Unassigned
V037313	complete	virome_only	Caudovirales	Siphoviridae
V037355	medium-quality	virome_only	Caudovirales	Siphoviridae
V037464	high-quality	both	Caudovirales	Myoviridae
V037466	high-quality	virome_only	Caudovirales	Siphoviridae
V037512	low-quality	virome_only	Caudovirales	Siphoviridae
V037552	complete	both	Caudovirales	Siphoviridae
V037574	high-quality	both	Caudovirales	Siphoviridae
V037597	complete	virome_only	Caudovirales	Siphoviridae
V037674	low-quality	virome_only	Caudovirales	Siphoviridae
V037703	high-quality	both	Caudovirales	Siphoviridae
V037740	low-quality	virome_only	Caudovirales	Podoviridae

V037781	complete	virome_only	Caudovirales	Myoviridae
V037784	complete	both	Caudovirales	Siphoviridae
V037803	low-quality	virome_only	Caudovirales	Siphoviridae
V037810	low-quality	virome_only	Caudovirales	Unassigned
V037874	low-quality	both	Caudovirales	Unassigned
V037891	complete	virome_only	Caudovirales	Siphoviridae
V037917	low-quality	virome_only	Caudovirales	Siphoviridae
V037981	low-quality	virome_only	Caudovirales	Siphoviridae
V037986	complete	both	Caudovirales	Myoviridae
V038000	low-quality	virome_only	Caudovirales	Siphoviridae
V038002	low-quality	virome_only	Caudovirales	Myoviridae
V038006	not-determined	virome_only	Caudovirales	Unassigned
V038050	complete	virome_only	Caudovirales	Siphoviridae
V038057	low-quality	virome_only	Caudovirales	Myoviridae
V038157	not-determined	virome_only	Caudovirales	Myoviridae
V038168	not-determined	virome_only	Caudovirales	Unassigned
V038192	low-quality	virome_only	Caudovirales	Siphoviridae
V038210	not-determined	virome_only	Caudovirales	Myoviridae
V038219	high-quality	virome_only	Caudovirales	Siphoviridae
V038262	complete	both	Caudovirales	Myoviridae
V038284	complete	virome_only	Petitvirales	Microviridae
V038305	low-quality	virome_only	Caudovirales	Siphoviridae
V038335	not-determined	virome_only	Caudovirales	Myoviridae
V038342	high-quality	both	Caudovirales	Siphoviridae
V038373	complete	virome_only	Petitvirales	Microviridae
V038375	low-quality	virome_only	not classified	not classified
V038445	low-quality	virome_only	Caudovirales	Unassigned
V038458	not-determined	virome_only	Caudovirales	Unassigned
V038479	low-quality	virome_only	Caudovirales	Siphoviridae
V038516	low-quality	virome_only	Caudovirales	Unassigned
V038539	medium-quality	virome_only	Caudovirales	Siphoviridae
V038549	low-quality	virome_only	Caudovirales	Unassigned
V038563	low-quality	virome_only	Caudovirales	Siphoviridae
V038602	complete	virome_only	Caudovirales	Siphoviridae
V038621	low-quality	virome_only	Caudovirales	Siphoviridae
V038625	low-quality	virome_only	Caudovirales	Unassigned
V038633	complete	both	Caudovirales	Siphoviridae
V038659	low-quality	virome_only	Caudovirales	Siphoviridae
V038668	complete	virome_only	Caudovirales	Siphoviridae
V038678	not-determined	virome_only	Caudovirales	Siphoviridae
V038703	not-determined	virome_only	Caudovirales	Siphoviridae
V038706	not-determined	virome_only	Caudovirales	Siphoviridae
V038708	low-quality	virome_only	Caudovirales	Unassigned
V038709	high-quality	virome_only	Caudovirales	Siphoviridae
V038735	low-quality	virome_only	Caudovirales	Unassigned
V038736	complete	virome_only	not classified	not classified
V038743	low-quality	virome_only	Caudovirales	Siphoviridae

V038754	low-quality	virome_only	Caudovirales	Siphoviridae
V038901	low-quality	virome_only	not classified	not classified
V038929	high-quality	virome_only	Caudovirales	Siphoviridae
V038934	low-quality	virome_only	Caudovirales	Siphoviridae
V038938	medium-quality	virome_only	Caudovirales	Siphoviridae
V038984	low-quality	virome_only	Caudovirales	Siphoviridae
V039003	not-determined	virome_only	Caudovirales	Unassigned
V039022	not-determined	virome_only	Caudovirales	Unassigned
V039112	low-quality	virome_only	Caudovirales	Siphoviridae
V039158	low-quality	virome_only	Caudovirales	Siphoviridae
V039163	not-determined	virome_only	Caudovirales	Siphoviridae
V039166	high-quality	virome_only	no_order_Anelloviridae	Anelloviridae
V039245	low-quality	virome_only	Caudovirales	Unassigned
V039368	low-quality	virome_only	Caudovirales	Siphoviridae
V039369	complete	both	Caudovirales	Siphoviridae
V039375	low-quality	virome_only	Caudovirales	Siphoviridae
V039404	low-quality	virome_only	Caudovirales	Siphoviridae
V039538	low-quality	virome_only	Caudovirales	Siphoviridae
V039550	high-quality	both	Caudovirales	Siphoviridae
V039567	medium-quality	virome_only	Caudovirales	Siphoviridae
V039580	not-determined	virome_only	Caudovirales	Siphoviridae
V039592	low-quality	virome_only	Caudovirales	Siphoviridae
V039599	low-quality	virome_only	Caudovirales	Siphoviridae
V039600	high-quality	both	Caudovirales	Siphoviridae
V039615	low-quality	virome_only	Caudovirales	Myoviridae
V039647	low-quality	virome_only	Caudovirales	Siphoviridae
V039654	low-quality	virome_only	Caudovirales	Siphoviridae
V039662	not-determined	virome_only	Caudovirales	Myoviridae
V039671	low-quality	virome_only	Caudovirales	Siphoviridae
V039689	low-quality	virome_only	Caudovirales	Myoviridae
V039708	low-quality	both	Caudovirales	Myoviridae
V039720	not-determined	virome_only	Caudovirales	Siphoviridae
V039772	high-quality	both	Caudovirales	Siphoviridae
V039812	high-quality	virome_only	no_order_Anelloviridae	Anelloviridae
V039841	medium-quality	both	Caudovirales	Myoviridae
V039842	low-quality	virome_only	Caudovirales	Siphoviridae
V039890	high-quality	virome_only	Petitvirales	Microviridae
V039895	low-quality	virome_only	Caudovirales	Siphoviridae
V039900	medium-quality	virome_only	Caudovirales	Siphoviridae
V039926	medium-quality	both	Caudovirales	Podoviridae
V039948	low-quality	virome_only	Caudovirales	Siphoviridae
V039978	high-quality	virome_only	Caudovirales	Siphoviridae
V039980	not-determined	virome_only	Caudovirales	Unassigned
V040004	low-quality	virome_only	Caudovirales	Siphoviridae
V040009	low-quality	virome_only	Caudovirales	Unassigned
V040145	low-quality	virome_only	Caudovirales	Siphoviridae
V040175	low-quality	virome_only	Caudovirales	Myoviridae

V040180	low-quality	virome_only	Caudovirales	Myoviridae
V040255	low-quality	virome_only	Caudovirales	Siphoviridae
V040258	not-determined	virome_only	Caudovirales	Myoviridae
V040264	medium-quality	virome_only	not classified	not classified
V040306	high-quality	virome_only	Caudovirales	Siphoviridae
V040337	medium-quality	both	Caudovirales	Siphoviridae
V040383	low-quality	virome_only	Caudovirales	Siphoviridae
V040413	medium-quality	virome_only	Cirlivirales	Circoviridae
V040446	low-quality	virome_only	Caudovirales	Siphoviridae
V040481	low-quality	virome_only	Caudovirales	Siphoviridae
V040540	medium-quality	both	Caudovirales	Siphoviridae
V040545	low-quality	both	Caudovirales	Siphoviridae
V040549	high-quality	virome_only	Geplafuvirales	Genomoviridae
V040573	complete	both	Caudovirales	Siphoviridae
V040607	low-quality	virome_only	Caudovirales	Siphoviridae
V040633	low-quality	virome_only	Caudovirales	Siphoviridae
V040648	not-determined	virome_only	Caudovirales	Siphoviridae
V040659	high-quality	virome_only	Cirlivirales	Circoviridae
V040688	high-quality	virome_only	Caudovirales	Siphoviridae
V040691	low-quality	virome_only	Caudovirales	Siphoviridae
V040701	not-determined	virome_only	Caudovirales	Siphoviridae
V040731	not-determined	virome_only	no_order_Phycodnaviridae	Phycodnaviridae
V040756	medium-quality	both	Caudovirales	Siphoviridae
V040764	high-quality	both	Caudovirales	Myoviridae
V040767	medium-quality	virome_only	Caudovirales	Siphoviridae
V040791	low-quality	virome_only	Caudovirales	Siphoviridae
V040811	low-quality	virome_only	Caudovirales	Myoviridae
V040884	medium-quality	virome_only	Caudovirales	Siphoviridae
V040931	complete	virome_only	Caudovirales	Siphoviridae
V040966	medium-quality	virome_only	Petitvirales	Microviridae
V040967	low-quality	virome_only	Caudovirales	Siphoviridae
V040979	low-quality	virome_only	Caudovirales	Siphoviridae
V040991	medium-quality	virome_only	Caudovirales	Siphoviridae
V041092	low-quality	virome_only	Caudovirales	Siphoviridae
V041095	low-quality	virome_only	Caudovirales	Siphoviridae
V041134	low-quality	virome_only	Caudovirales	Podoviridae
V041170	low-quality	virome_only	Caudovirales	Siphoviridae
V041183	not-determined	virome_only	Caudovirales	Siphoviridae
V041274	low-quality	virome_only	Caudovirales	Myoviridae
V041306	low-quality	virome_only	Caudovirales	Siphoviridae
V041318	low-quality	both	Caudovirales	Siphoviridae
V041370	medium-quality	virome_only	Caudovirales	Siphoviridae
V041399	low-quality	virome_only	Caudovirales	Siphoviridae
V041434	low-quality	virome_only	Caudovirales	Siphoviridae
V041437	low-quality	virome_only	Caudovirales	Siphoviridae
V041446	low-quality	virome_only	Caudovirales	Myoviridae
V041485	low-quality	virome_only	Caudovirales	Siphoviridae

V041620	high-quality	both	Caudovirales	Siphoviridae
V041697	low-quality	virome_only	Caudovirales	Siphoviridae
V041731	medium-quality	virome_only	Caudovirales	Myoviridae
V041752	low-quality	virome_only	Caudovirales	Siphoviridae
V041822	low-quality	virome_only	Caudovirales	Siphoviridae
V041833	low-quality	virome_only	Caudovirales	Siphoviridae
V041894	low-quality	virome_only	Caudovirales	Siphoviridae
V041901	not-determined	virome_only	Caudovirales	Siphoviridae
V041919	low-quality	virome_only	Caudovirales	Siphoviridae
V041956	low-quality	virome_only	Caudovirales	Myoviridae
V041994	low-quality	virome_only	Caudovirales	Siphoviridae
V042042	not-determined	virome_only	Caudovirales	Myoviridae
V042076	low-quality	virome_only	Caudovirales	Siphoviridae
V042090	low-quality	virome_only	Caudovirales	Siphoviridae
V042095	low-quality	virome_only	Caudovirales	Siphoviridae
V042116	medium-quality	virome_only	Caudovirales	Siphoviridae
V042128	low-quality	virome_only	Caudovirales	Siphoviridae
V042194	low-quality	virome_only	Caudovirales	Siphoviridae
V042201	low-quality	virome_only	Caudovirales	Unassigned
V042208	low-quality	virome_only	Caudovirales	Podoviridae
V042242	low-quality	virome_only	Caudovirales	Siphoviridae
V042246	complete	virome_only	Caudovirales	Siphoviridae
V042341	low-quality	virome_only	Caudovirales	Unassigned
V042350	medium-quality	virome_only	Caudovirales	Siphoviridae
V042371	low-quality	virome_only	not classified	not classified
V042375	low-quality	virome_only	Caudovirales	Unassigned
V042414	high-quality	virome_only	Caudovirales	Siphoviridae
V042492	high-quality	virome_only	Caudovirales	Myoviridae
V042497	high-quality	virome_only	Caudovirales	Siphoviridae
V042507	low-quality	virome_only	Caudovirales	Siphoviridae
V042515	low-quality	virome_only	Caudovirales	Siphoviridae
V042527	not-determined	virome_only	not classified	not classified
V042543	low-quality	virome_only	Caudovirales	Siphoviridae
V042564	not-determined	virome_only	Caudovirales	Siphoviridae
V042586	medium-quality	both	Caudovirales	Siphoviridae
V042619	medium-quality	virome_only	Caudovirales	Myoviridae
V042638	low-quality	virome_only	Caudovirales	Siphoviridae
V042668	low-quality	virome_only	Caudovirales	Myoviridae
V042669	complete	both	Caudovirales	Siphoviridae
V042682	low-quality	virome_only	Caudovirales	Myoviridae
V042688	low-quality	virome_only	Caudovirales	Siphoviridae
V042710	low-quality	virome_only	Caudovirales	Siphoviridae
V042721	medium-quality	both	Caudovirales	Siphoviridae
V042723	low-quality	virome_only	Caudovirales	Myoviridae
V042785	not-determined	virome_only	Caudovirales	Unassigned
V042808	low-quality	virome_only	Caudovirales	Unassigned
V042839	low-quality	virome_only	Caudovirales	Siphoviridae

V042842	low-quality	virome_only	Caudovirales	Siphoviridae
V042945	low-quality	virome_only	Caudovirales	Siphoviridae
V042952	low-quality	virome_only	Caudovirales	Siphoviridae
V042985	complete	both	Caudovirales	Siphoviridae
V042995	low-quality	virome_only	Caudovirales	Siphoviridae
V043022	low-quality	virome_only	Caudovirales	Siphoviridae
V043081	low-quality	virome_only	Caudovirales	Myoviridae
V043083	not-determined	virome_only	Caudovirales	Myoviridae
V043097	low-quality	virome_only	Caudovirales	Unassigned
V043195	not-determined	virome_only	Caudovirales	Siphoviridae
V043205	low-quality	virome_only	Caudovirales	Myoviridae
V043212	not-determined	virome_only	Caudovirales	Siphoviridae
V043219	low-quality	virome_only	Caudovirales	Unassigned
V043232	low-quality	virome_only	Caudovirales	Siphoviridae
V043293	complete	both	Caudovirales	Siphoviridae
V043321	low-quality	virome_only	Caudovirales	Siphoviridae
V043336	low-quality	virome_only	Caudovirales	Unassigned
V043388	complete	virome_only	not classified	not classified
V043435	complete	both	Caudovirales	Siphoviridae
V043443	complete	virome_only	Petitvirales	Microviridae
V043447	low-quality	virome_only	Caudovirales	Siphoviridae
V043484	low-quality	virome_only	Caudovirales	Myoviridae
V043493	low-quality	virome_only	Caudovirales	Myoviridae
V043558	medium-quality	virome_only	Caudovirales	Unassigned
V043560	low-quality	virome_only	Caudovirales	Myoviridae
V043562	complete	virome_only	Petitvirales	Microviridae
V043566	complete	virome_only	Petitvirales	Microviridae
V043575	complete	virome_only	Caudovirales	Unassigned
V043576	low-quality	both	Caudovirales	Siphoviridae
V043578	complete	virome_only	Petitvirales	Microviridae
V043584	complete	virome_only	Cirlivirales	Circoviridae
V043597	complete	virome_only	Caudovirales	Siphoviridae
V043599	complete	virome_only	Petitvirales	Microviridae
V043604	medium-quality	virome_only	Caudovirales	Siphoviridae
V043606	not-determined	virome_only	Caudovirales	Siphoviridae
V043616	complete	virome_only	Cirlivirales	Circoviridae
V043617	medium-quality	virome_only	Cirlivirales	Circoviridae
V043618	complete	virome_only	Caudovirales	Siphoviridae
V043623	complete	virome_only	Caudovirales	Siphoviridae
V043625	complete	virome_only	Caudovirales	Siphoviridae
V043636	complete	virome_only	Caudovirales	Siphoviridae
V043642	complete	virome_only	Caudovirales	Siphoviridae
V043651	complete	virome_only	Caudovirales	Siphoviridae
V043664	complete	virome_only	Petitvirales	Microviridae
V043665	complete	virome_only	Cirlivirales	Circoviridae
V043667	complete	virome_only	Petitvirales	Microviridae
V043668	complete	virome_only	Petitvirales	Microviridae

V043674	complete	virome_only	Cirlivirales	Circoviridae
V043680	complete	virome_only	not classified	not classified
V043687	complete	virome_only	Cirlivirales	Circoviridae
V043690	complete	virome_only	not classified	not classified
V043694	complete	virome_only	Petitvirales	Microviridae
V043742	complete	virome_only	Caudovirales	Siphoviridae
V043754	complete	virome_only	Cirlivirales	Circoviridae
V043818	low-quality	virome_only	Caudovirales	Podoviridae
V043835	low-quality	virome_only	Caudovirales	Siphoviridae
V043837	low-quality	virome_only	Caudovirales	Siphoviridae
V043841	medium-quality	virome_only	Caudovirales	Siphoviridae
V043847	low-quality	virome_only	Caudovirales	Myoviridae
V043858	low-quality	both	Caudovirales	Siphoviridae
V043883	low-quality	virome_only	Caudovirales	Siphoviridae
V043884	complete	virome_only	no_order_Anelloviridae	Anelloviridae
V043889	medium-quality	both	Caudovirales	Siphoviridae
V043898	low-quality	virome_only	Caudovirales	Siphoviridae
V043901	medium-quality	virome_only	Caudovirales	Podoviridae
V043903	low-quality	virome_only	Caudovirales	Siphoviridae
V043904	low-quality	virome_only	Caudovirales	Siphoviridae
V043905	low-quality	virome_only	not classified	not classified
V043910	low-quality	virome_only	Caudovirales	Siphoviridae
V043923	low-quality	virome_only	Caudovirales	Siphoviridae
V043931	low-quality	virome_only	Caudovirales	Siphoviridae
V043937	low-quality	virome_only	Caudovirales	Siphoviridae
V043949	low-quality	virome_only	Caudovirales	Siphoviridae
V043954	low-quality	virome_only	Caudovirales	Myoviridae
V043970	low-quality	virome_only	Caudovirales	Siphoviridae
V043971	low-quality	virome_only	Caudovirales	Siphoviridae
V043974	low-quality	virome_only	Caudovirales	Siphoviridae
V043980	low-quality	virome_only	Caudovirales	Unassigned
V043991	low-quality	virome_only	Caudovirales	Siphoviridae
V043996	low-quality	virome_only	Caudovirales	Siphoviridae
V043998	low-quality	virome_only	Caudovirales	Myoviridae
V043999	low-quality	virome_only	Caudovirales	Unassigned
V044008	complete	virome_only	Caudovirales	Siphoviridae
V044010	low-quality	virome_only	Caudovirales	Siphoviridae
V044034	low-quality	virome_only	Caudovirales	Siphoviridae
V044040	low-quality	virome_only	Caudovirales	Myoviridae
V044057	low-quality	virome_only	Caudovirales	Podoviridae
V044082	low-quality	virome_only	Caudovirales	Siphoviridae
V044088	low-quality	virome_only	Caudovirales	Myoviridae
V044094	low-quality	virome_only	Caudovirales	Siphoviridae
V044098	high-quality	virome_only	Petitvirales	Microviridae
V044101	medium-quality	both	Caudovirales	Siphoviridae
V044102	medium-quality	virome_only	Caudovirales	Siphoviridae
V044123	low-quality	both	Caudovirales	Siphoviridae

V044149	low-quality	virome_only	Caudovirales	Siphoviridae
V044188	low-quality	virome_only	Caudovirales	Siphoviridae
V044190	low-quality	virome_only	not classified	not classified
V044191	medium-quality	virome_only	Caudovirales	Siphoviridae
V044212	medium-quality	virome_only	Caudovirales	Siphoviridae
V044231	low-quality	virome_only	Caudovirales	Siphoviridae
V044254	complete	both	Caudovirales	Siphoviridae
V044283	low-quality	virome_only	Caudovirales	Siphoviridae
V044284	medium-quality	virome_only	Caudovirales	Siphoviridae
V044292	low-quality	virome_only	Tubulavirales	Inoviridae
V044295	low-quality	virome_only	Caudovirales	Myoviridae
V044298	high-quality	virome_only	Caudovirales	Siphoviridae
V044301	low-quality	virome_only	Caudovirales	Siphoviridae
V044305	complete	both	Caudovirales	Siphoviridae
V044329	low-quality	virome_only	Caudovirales	Siphoviridae
V044333	low-quality	virome_only	Caudovirales	Myoviridae
V044339	low-quality	virome_only	Caudovirales	Myoviridae
V044350	low-quality	both	Caudovirales	Siphoviridae
V044356	high-quality	virome_only	Caudovirales	Siphoviridae
V044364	low-quality	virome_only	Caudovirales	Siphoviridae
V044365	medium-quality	virome_only	Caudovirales	Siphoviridae
V044381	medium-quality	both	Caudovirales	Siphoviridae
V044382	high-quality	virome_only	Caudovirales	Siphoviridae
V044396	low-quality	virome_only	Caudovirales	Siphoviridae
V044401	high-quality	both	Caudovirales	Siphoviridae
V044415	medium-quality	virome_only	Caudovirales	Podoviridae
V044431	complete	virome_only	Caudovirales	Siphoviridae
V044439	complete	virome_only	not classified	not classified
V044441	low-quality	virome_only	Caudovirales	Siphoviridae
V044450	low-quality	virome_only	Caudovirales	Siphoviridae
V044453	complete	virome_only	Caudovirales	Siphoviridae
V044455	complete	virome_only	no_order_Anelloviridae	Anelloviridae
V044456	complete	virome_only	not classified	not classified
V044464	medium-quality	both	Caudovirales	Siphoviridae
V044593	low-quality	virome_only	Caudovirales	Siphoviridae
V044598	low-quality	virome_only	Caudovirales	Siphoviridae
V044607	low-quality	virome_only	Caudovirales	Myoviridae
V044696	medium-quality	virome_only	Caudovirales	Siphoviridae
V044738	low-quality	virome_only	Caudovirales	Siphoviridae
V044766	low-quality	virome_only	Caudovirales	Siphoviridae
V044851	medium-quality	virome_only	Caudovirales	Myoviridae
V044939	low-quality	virome_only	Caudovirales	Siphoviridae
V045022	medium-quality	virome_only	Caudovirales	Siphoviridae
V045032	low-quality	virome_only	Caudovirales	Siphoviridae
V045039	medium-quality	both	Caudovirales	Myoviridae
V045093	medium-quality	virome_only	Caudovirales	Siphoviridae
V045118	high-quality	both	Caudovirales	Myoviridae

V045148	low-quality	virome_only	Caudovirales	Siphoviridae
V045156	high-quality	virome_only	Caudovirales	Podoviridae
V045158	low-quality	virome_only	Caudovirales	Siphoviridae
V045200	complete	both	Caudovirales	Siphoviridae
V045211	low-quality	virome_only	Caudovirales	Siphoviridae
V045223	low-quality	virome_only	Caudovirales	Siphoviridae
V045239	not-determined	virome_only	not classified	not classified
V045250	low-quality	virome_only	Caudovirales	Siphoviridae
V045252	medium-quality	virome_only	Caudovirales	Siphoviridae
V045261	low-quality	virome_only	Caudovirales	Unassigned
V045288	high-quality	both	Caudovirales	Myoviridae
V045478	low-quality	virome_only	Caudovirales	Siphoviridae
V045485	low-quality	virome_only	Caudovirales	Siphoviridae
V045495	medium-quality	both	Caudovirales	Siphoviridae
V045501	high-quality	both	Caudovirales	Podoviridae
V045569	low-quality	virome_only	not classified	not classified
V045608	medium-quality	both	Caudovirales	Myoviridae
V045659	high-quality	both	Caudovirales	Siphoviridae
V045671	low-quality	virome_only	Caudovirales	Siphoviridae
V045683	low-quality	virome_only	Caudovirales	Siphoviridae
V045770	low-quality	virome_only	Caudovirales	Podoviridae
V045799	not-determined	virome_only	Caudovirales	Myoviridae
V045802	low-quality	virome_only	Caudovirales	Podoviridae
V045836	low-quality	virome_only	Caudovirales	Myoviridae
V045905	low-quality	virome_only	Caudovirales	Podoviridae
V045910	medium-quality	both	Caudovirales	Siphoviridae
V046000	not-determined	virome_only	Caudovirales	Myoviridae
V046013	not-determined	virome_only	Caudovirales	Unassigned
V046046	medium-quality	virome_only	Caudovirales	Siphoviridae
V046051	low-quality	virome_only	Caudovirales	Siphoviridae
V046054	low-quality	virome_only	Caudovirales	Siphoviridae
V046060	low-quality	virome_only	Caudovirales	Unassigned
V046140	medium-quality	virome_only	Caudovirales	Siphoviridae
V046193	low-quality	virome_only	Caudovirales	Siphoviridae
V046232	medium-quality	virome_only	Caudovirales	Siphoviridae
V046298	complete	virome_only	Caudovirales	Siphoviridae
V046321	complete	virome_only	Caudovirales	Myoviridae
V046323	low-quality	virome_only	Caudovirales	Siphoviridae
V046361	low-quality	virome_only	Caudovirales	Unassigned
V046370	low-quality	virome_only	Caudovirales	Unassigned
V046384	not-determined	virome_only	Caudovirales	Unassigned
V046449	complete	both	Caudovirales	Siphoviridae
V046450	medium-quality	both	Caudovirales	Siphoviridae
V046517	not-determined	virome_only	Caudovirales	Siphoviridae
V046533	medium-quality	virome_only	Caudovirales	Myoviridae
V046542	high-quality	both	Caudovirales	Siphoviridae
V046561	low-quality	virome_only	Caudovirales	Siphoviridae

V046583	not-determined	virome_only	Caudovirales	Siphoviridae
V046585	low-quality	virome_only	Caudovirales	Myoviridae
V046594	medium-quality	virome_only	Caudovirales	Siphoviridae
V046602	low-quality	virome_only	Caudovirales	Siphoviridae
V046642	low-quality	virome_only	Caudovirales	Siphoviridae
V046644	high-quality	virome_only	Caudovirales	Siphoviridae
V046645	low-quality	virome_only	Caudovirales	Siphoviridae
V046662	medium-quality	both	Caudovirales	Siphoviridae
V046675	high-quality	both	Caudovirales	Siphoviridae
V046705	medium-quality	virome_only	Caudovirales	Podoviridae
V046718	not-determined	virome_only	no_order_Phycodnaviridae	Phycodnaviridae
V046720	low-quality	virome_only	Caudovirales	Myoviridae
V046756	high-quality	both	Caudovirales	Siphoviridae
V046817	low-quality	virome_only	Caudovirales	Siphoviridae
V046841	low-quality	both	Caudovirales	Siphoviridae
V046864	low-quality	virome_only	Caudovirales	Siphoviridae
V046919	complete	both	Caudovirales	Siphoviridae
V046921	low-quality	both	Caudovirales	Siphoviridae
V047046	medium-quality	virome_only	Petitvirales	Microviridae
V047048	low-quality	virome_only	Caudovirales	Siphoviridae
V047090	high-quality	both	Caudovirales	Siphoviridae
V047117	low-quality	virome_only	Caudovirales	Siphoviridae
V047125	complete	both	Caudovirales	Siphoviridae
V047130	low-quality	virome_only	Caudovirales	Siphoviridae
V047132	low-quality	virome_only	Caudovirales	Siphoviridae
V047157	low-quality	virome_only	Caudovirales	Siphoviridae
V047164	medium-quality	both	Caudovirales	Siphoviridae
V047165	medium-quality	virome_only	Caudovirales	Siphoviridae
V047186	low-quality	virome_only	Caudovirales	Siphoviridae
V047188	high-quality	both	Caudovirales	Siphoviridae
V047206	low-quality	virome_only	Caudovirales	Siphoviridae
V047238	low-quality	virome_only	Caudovirales	Siphoviridae
V047246	low-quality	virome_only	Caudovirales	Siphoviridae
V047270	low-quality	virome_only	Caudovirales	Myoviridae
V047273	low-quality	virome_only	Caudovirales	Siphoviridae
V047280	low-quality	virome_only	Caudovirales	Siphoviridae
V047310	low-quality	virome_only	Caudovirales	Siphoviridae
V047422	medium-quality	both	Caudovirales	Siphoviridae
V047428	low-quality	virome_only	Caudovirales	Siphoviridae
V047455	high-quality	both	Caudovirales	Siphoviridae
V047540	low-quality	virome_only	Caudovirales	Siphoviridae
V047546	low-quality	virome_only	Caudovirales	Siphoviridae
V047565	low-quality	virome_only	Caudovirales	Unassigned
V047580	low-quality	virome_only	Caudovirales	Myoviridae
V047584	low-quality	virome_only	not classified	not classified
V047597	low-quality	virome_only	Caudovirales	Siphoviridae
V047631	not-determined	virome_only	not classified	not classified

V047688	medium-quality	both	Caudovirales	Myoviridae
V047698	not-determined	virome_only	Caudovirales	Podoviridae
V047699	high-quality	both	Caudovirales	Siphoviridae
V047715	high-quality	both	Caudovirales	Siphoviridae
V047740	high-quality	virome_only	Caudovirales	Siphoviridae
V047748	complete	virome_only	Caudovirales	Siphoviridae
V047754	high-quality	virome_only	Caudovirales	Myoviridae
V047755	complete	both	Caudovirales	Siphoviridae
V047765	complete	virome_only	not classified	not classified
V047770	complete	virome_only	no_order_Anelloviridae	Anelloviridae

max_abundance	contains_integrase	prophage_flanking
0.00722	FALSE	FALSE
0.028797	FALSE	FALSE
0.005384	FALSE	FALSE
0.012084	FALSE	FALSE
0.012937	FALSE	FALSE
0.011643	FALSE	FALSE
0.009704	FALSE	FALSE
0.168333	FALSE	FALSE
0.006392	FALSE	FALSE
0.012362	FALSE	FALSE
0.00987	FALSE	FALSE
0.08544	FALSE	FALSE
0.006358	FALSE	TRUE
0.224443	FALSE	FALSE
0.145884	TRUE	TRUE
0.013634	FALSE	TRUE
0.052191	FALSE	FALSE
0.11177	FALSE	FALSE
0.009446	FALSE	FALSE
0.220961	FALSE	FALSE
0.116663	FALSE	TRUE
0.023429	FALSE	TRUE
0.007481	FALSE	FALSE
0.00305	FALSE	FALSE
0.005664	FALSE	TRUE
0.008904	FALSE	FALSE
0.003901	FALSE	FALSE
0.168863	TRUE	FALSE
0.00853	FALSE	TRUE
0.008477	FALSE	FALSE
0.005275	FALSE	FALSE
0.046799	FALSE	FALSE
0.007656	FALSE	FALSE
0.01119	FALSE	FALSE
0.004207	FALSE	FALSE
0.021522	FALSE	TRUE
0.012801	FALSE	FALSE
0.01468	FALSE	FALSE
0.025434	FALSE	TRUE
0.014377	FALSE	TRUE
0.014853	FALSE	TRUE
0.108275	FALSE	FALSE
0.023494	FALSE	TRUE
0.010538	FALSE	TRUE
0.003543	FALSE	FALSE
0.009705	FALSE	FALSE

87.976864	FALSE	FALSE
0.014748	FALSE	FALSE
0.006925	FALSE	FALSE
0.002778	FALSE	FALSE
0.042027	FALSE	FALSE
0.033381	FALSE	TRUE
0.006305	FALSE	FALSE
0.100085	FALSE	FALSE
0.009443	FALSE	FALSE
0.012546	FALSE	FALSE
0.054341	FALSE	FALSE
0.044233	FALSE	TRUE
0.072677	FALSE	TRUE
0.005554	FALSE	FALSE
0.216801	FALSE	FALSE
0.005977	FALSE	FALSE
0.004045	FALSE	FALSE
0.280353	FALSE	TRUE
0.114182	FALSE	TRUE
0.005418	FALSE	FALSE
0.010144	FALSE	FALSE
0.003108	FALSE	FALSE
0.013111	FALSE	TRUE
0.014344	FALSE	FALSE
0.029685	FALSE	FALSE
0.011001	FALSE	FALSE
0.072253	FALSE	TRUE
0.019454	FALSE	FALSE
0.004429	FALSE	FALSE
0.017963	FALSE	TRUE
0.013077	FALSE	FALSE
0.010124	FALSE	FALSE
0.079389	FALSE	FALSE
0.018409	FALSE	TRUE
0.023607	FALSE	TRUE
0.076691	FALSE	TRUE
0.00918	FALSE	TRUE
0.015202	FALSE	FALSE
0.026907	FALSE	FALSE
0.014925	FALSE	FALSE
0.02027	FALSE	TRUE
0.02614	FALSE	TRUE
0.015428	FALSE	FALSE
0.021386	FALSE	TRUE
0.020603	FALSE	FALSE
0.024757	FALSE	FALSE
0.006662	FALSE	FALSE

0.013151	FALSE	FALSE
0.005725	FALSE	FALSE
0.023288	FALSE	TRUE
0.022143	FALSE	FALSE
0.011962	FALSE	FALSE
0.011588	FALSE	FALSE
0.009115	FALSE	FALSE
0.009442	FALSE	FALSE
0.067619	FALSE	FALSE
0.007927	FALSE	FALSE
0.01108	FALSE	TRUE
0.015098	FALSE	FALSE
0.056931	FALSE	FALSE
5.467608	FALSE	FALSE
0.004385	TRUE	FALSE
0.002656	FALSE	FALSE
0.002096	FALSE	FALSE
0.008837	FALSE	TRUE
0.002429	FALSE	TRUE
0.004457	FALSE	TRUE
4.12E-04	FALSE	FALSE
0.051195	FALSE	TRUE
3.28E-04	FALSE	FALSE
4.17E-04	FALSE	FALSE
0.00605	FALSE	FALSE
0.001832	FALSE	FALSE
0.001745	FALSE	FALSE
0.001064	FALSE	FALSE
0.001273	FALSE	FALSE
0.007665	FALSE	FALSE
0.001899	FALSE	TRUE
0.002145	FALSE	TRUE
0.053083	FALSE	FALSE
7.69E-04	FALSE	FALSE
0.006825	FALSE	TRUE
5.321707	FALSE	TRUE
0.015669	FALSE	TRUE
0.002957	FALSE	TRUE
0.050666	FALSE	TRUE
0.146548	FALSE	TRUE
0.002457	FALSE	TRUE
0.002738	FALSE	FALSE
2.230125	FALSE	FALSE
8.02E-04	FALSE	FALSE
6.84E-04	FALSE	FALSE
0.01558	FALSE	FALSE
0.015623	FALSE	TRUE

0.008145	FALSE	TRUE
0.021634	FALSE	TRUE
0.009687	FALSE	FALSE
0.004339	FALSE	FALSE
0.524706	FALSE	TRUE
0.127178	FALSE	FALSE
0.274313	FALSE	FALSE
0.013737	FALSE	FALSE
0.015776	FALSE	TRUE
0.005497	FALSE	FALSE
0.002665	TRUE	FALSE
0.001136	FALSE	FALSE
0.014179	FALSE	FALSE
0.13586	FALSE	TRUE
0.705339	FALSE	TRUE
0.003896	FALSE	FALSE
0.040564	FALSE	TRUE
0.001679	FALSE	FALSE
6.93E-04	FALSE	FALSE
0.042659	FALSE	FALSE
0.019014	FALSE	TRUE
5.08E-04	FALSE	FALSE
0.008478	FALSE	FALSE
0.002505	FALSE	FALSE
8.34E-04	FALSE	FALSE
0.06271	FALSE	FALSE
0.001433	FALSE	TRUE
0.003027	TRUE	TRUE
0.007431	FALSE	TRUE
1.213206	FALSE	TRUE
0.174346	FALSE	TRUE
0.022949	FALSE	FALSE
0.004129	FALSE	TRUE
7.22E-04	FALSE	FALSE
6.80E-04	FALSE	FALSE
0.002695	FALSE	FALSE
0.002092	FALSE	FALSE
0.135677	FALSE	FALSE
0.002251	FALSE	FALSE
5.40E-04	FALSE	FALSE
0.002251	FALSE	TRUE
0.002669	FALSE	TRUE
0.00676	FALSE	TRUE
7.28E-04	FALSE	FALSE
0.046518	FALSE	TRUE
5.28E-04	FALSE	FALSE
0.010629	FALSE	TRUE

0.001574	FALSE	FALSE
0.003232	FALSE	TRUE
7.09E-04	FALSE	TRUE
0.001457	FALSE	FALSE
6.34E-04	FALSE	FALSE
0.011387	FALSE	TRUE
0.022133	FALSE	FALSE
0.033494	FALSE	FALSE
0.001165	FALSE	FALSE
0.006761	FALSE	TRUE
0.002648	FALSE	TRUE
0.001183	FALSE	TRUE
0.003178	FALSE	FALSE
0.011121	FALSE	TRUE
0.002342	FALSE	FALSE
0.075248	FALSE	TRUE
5.87E-04	FALSE	FALSE
6.70E-04	FALSE	FALSE
0.026978	FALSE	TRUE
0.003378	FALSE	FALSE
0.013865	FALSE	TRUE
6.26E-04	FALSE	FALSE
0.017333	FALSE	TRUE
0.004565	FALSE	TRUE
0.061585	FALSE	FALSE
0.004681	FALSE	TRUE
0.007574	FALSE	TRUE
0.001135	FALSE	FALSE
3.85E-04	FALSE	FALSE
0.002084	FALSE	FALSE
0.004095	FALSE	TRUE
7.70E-04	FALSE	FALSE
0.003742	FALSE	TRUE
0.003118	FALSE	TRUE
0.003155	FALSE	TRUE
0.002633	FALSE	TRUE
0.001924	FALSE	FALSE
0.004868	FALSE	TRUE
0.047453	FALSE	FALSE
0.034152	FALSE	FALSE
0.015138	FALSE	FALSE
0.015669	FALSE	FALSE
0.002258	TRUE	TRUE
0.049665	FALSE	FALSE
7.43E-04	FALSE	FALSE
0.017485	FALSE	TRUE
3.39E-04	FALSE	FALSE

2.93E-04	FALSE	FALSE
0.033671	FALSE	TRUE
0.004143	FALSE	TRUE
4.90E-04	FALSE	FALSE
0.074984	FALSE	TRUE
0.003493	FALSE	TRUE
0.086629	FALSE	TRUE
9.57E-04	FALSE	FALSE
0.01279	FALSE	TRUE
0.308519	FALSE	TRUE
7.58E-04	FALSE	FALSE
0.001205	TRUE	FALSE
0.003514	FALSE	FALSE
0.001272	FALSE	FALSE
4.32E-04	FALSE	FALSE
0.00198	FALSE	TRUE
0.001025	FALSE	FALSE
0.001818	FALSE	TRUE
5.07E-04	FALSE	FALSE
0.006047	FALSE	TRUE
0.003181	FALSE	TRUE
0.001344	FALSE	FALSE
0.001443	FALSE	FALSE
5.90E-04	FALSE	FALSE
6.30E-04	FALSE	FALSE
0.088421	FALSE	TRUE
0.006109	FALSE	TRUE
0.024646	FALSE	TRUE
0.001338	FALSE	FALSE
5.02E-04	FALSE	FALSE
0.001299	FALSE	FALSE
0.011446	FALSE	TRUE
0.012216	FALSE	FALSE
0.001244	FALSE	TRUE
0.090518	TRUE	TRUE
0.001813	FALSE	FALSE
8.27E-04	FALSE	FALSE
0.00567	FALSE	FALSE
8.12E-04	FALSE	FALSE
0.006256	FALSE	TRUE
0.001452	FALSE	FALSE
5.71E-04	FALSE	FALSE
0.001496	FALSE	TRUE
9.06E-04	FALSE	FALSE
7.45E-04	FALSE	FALSE
0.002878	FALSE	FALSE
0.005123	FALSE	FALSE

5.283736	FALSE	TRUE
0.001765	FALSE	TRUE
5.26E-04	FALSE	FALSE
0.003063	FALSE	TRUE
0.001935	FALSE	TRUE
0.004647	FALSE	FALSE
0.002838	FALSE	TRUE
5.34E-04	FALSE	FALSE
0.0015	FALSE	TRUE
7.15E-04	FALSE	FALSE
0.130461	FALSE	FALSE
0.002594	FALSE	TRUE
0.001522	FALSE	FALSE
0.011252	FALSE	TRUE
0.001939	FALSE	TRUE
0.021616	FALSE	FALSE
0.007785	FALSE	FALSE
0.001753	FALSE	FALSE
0.001292	FALSE	FALSE
0.009423	FALSE	FALSE
0.001111	FALSE	FALSE
0.176207	FALSE	FALSE
6.43E-04	FALSE	FALSE
5.40E-04	FALSE	FALSE
0.050396	FALSE	FALSE
0.002006	FALSE	TRUE
0.006293	FALSE	FALSE
9.49E-04	FALSE	FALSE
0.04465	FALSE	TRUE
0.082749	FALSE	FALSE
7.24E-04	FALSE	FALSE
0.006394	FALSE	FALSE
0.005432	FALSE	TRUE
0.004662	FALSE	FALSE
9.89E-04	FALSE	FALSE
7.64E-04	FALSE	TRUE
0.00256	FALSE	TRUE
0.497305	FALSE	FALSE
8.47E-04	FALSE	FALSE
0.006671	FALSE	FALSE
0.023445	FALSE	TRUE
0.003146	FALSE	FALSE
0.021439	FALSE	TRUE
0.00136	FALSE	FALSE
0.002634	FALSE	FALSE
0.004537	FALSE	FALSE
0.00576	FALSE	TRUE

9.18E-04	FALSE	FALSE
5.10E-04	FALSE	FALSE
0.001863	FALSE	FALSE
9.60E-04	FALSE	FALSE
6.32E-04	FALSE	FALSE
0.008762	FALSE	FALSE
0.051804	FALSE	TRUE
0.027558	FALSE	FALSE
0.001143	FALSE	FALSE
0.365468	FALSE	FALSE
0.018196	FALSE	FALSE
7.87E-04	FALSE	FALSE
0.00164	FALSE	FALSE
0.00283	FALSE	FALSE
0.010925	FALSE	FALSE
0.001811	FALSE	FALSE
0.049072	FALSE	FALSE
0.002058	FALSE	FALSE
0.001492	FALSE	FALSE
3.08103	FALSE	TRUE
3.221395	FALSE	TRUE
0.001634	FALSE	FALSE
0.162524	FALSE	TRUE
0.014142	FALSE	FALSE
0.008788	FALSE	FALSE
0.00117	FALSE	FALSE
4.44E-04	FALSE	FALSE
0.007151	FALSE	FALSE
0.008364	FALSE	FALSE
0.004296	FALSE	FALSE
0.005494	FALSE	FALSE
0.001223	FALSE	FALSE
0.01191	FALSE	FALSE
0.024579	FALSE	FALSE
9.61E-04	FALSE	FALSE
0.00247	FALSE	FALSE
0.002573	FALSE	FALSE
0.008774	FALSE	FALSE
0.041547	FALSE	FALSE
0.001672	FALSE	FALSE
0.845563	FALSE	TRUE
6.91E-04	FALSE	FALSE
0.052878	FALSE	TRUE
0.017367	FALSE	TRUE
0.004026	FALSE	FALSE
0.035316	FALSE	FALSE
0.010718	FALSE	TRUE

0.020963	FALSE	FALSE
0.010875	FALSE	FALSE
0.064454	FALSE	TRUE
0.028883	FALSE	FALSE
0.0373	TRUE	TRUE
0.005585	FALSE	TRUE
0.001443	FALSE	TRUE
0.001969	FALSE	FALSE
0.002291	FALSE	FALSE
0.010759	FALSE	FALSE
0.001596	FALSE	FALSE
0.001697	FALSE	FALSE
0.022985	FALSE	TRUE
0.003457	FALSE	FALSE
0.009114	FALSE	FALSE
0.027254	FALSE	TRUE
0.002563	FALSE	FALSE
0.008002	FALSE	TRUE
9.90E-04	FALSE	FALSE
0.001628	FALSE	FALSE
0.014495	FALSE	FALSE
0.001781	FALSE	TRUE
0.002838	FALSE	FALSE
0.001009	FALSE	FALSE
0.003092	FALSE	TRUE
0.030444	FALSE	TRUE
8.44E-04	FALSE	FALSE
0.00673	FALSE	TRUE
0.015116	TRUE	FALSE
0.0104	FALSE	FALSE
0.034076	FALSE	TRUE
7.81E-04	FALSE	FALSE
0.022876	FALSE	TRUE
0.007503	FALSE	FALSE
0.001221	FALSE	FALSE
70.897574	FALSE	FALSE
0.002145	FALSE	FALSE
0.037399	FALSE	TRUE
0.042048	FALSE	FALSE
5.53E-04	FALSE	FALSE
0.00766	FALSE	TRUE
0.004905	FALSE	FALSE
0.010817	FALSE	TRUE
0.029159	FALSE	FALSE
4.88E-04	FALSE	FALSE
0.359449	FALSE	TRUE
0.006987	FALSE	TRUE

5.39E-04	FALSE	FALSE
0.001209	FALSE	FALSE
0.001058	FALSE	FALSE
0.00553	FALSE	TRUE
0.001731	FALSE	TRUE
0.052279	FALSE	FALSE
0.008663	FALSE	TRUE
0.001636	FALSE	FALSE
0.00119	FALSE	FALSE
6.16E-04	FALSE	FALSE
0.020533	FALSE	TRUE
0.006024	FALSE	TRUE
0.020692	FALSE	FALSE
0.002948	FALSE	TRUE
0.002969	FALSE	TRUE
0.008063	FALSE	TRUE
0.003135	FALSE	FALSE
0.006383	FALSE	TRUE
19.271672	FALSE	FALSE
0.001737	FALSE	FALSE
0.06972	FALSE	FALSE
0.01318	FALSE	TRUE
0.013114	FALSE	TRUE
0.002041	FALSE	FALSE
0.003394	FALSE	FALSE
9.90E-04	FALSE	FALSE
0.005398	FALSE	FALSE
0.00925	FALSE	FALSE
0.011817	FALSE	TRUE
0.006577	FALSE	FALSE
0.016268	FALSE	TRUE
0.052506	FALSE	FALSE
0.002144	FALSE	FALSE
0.012304	TRUE	TRUE
0.014491	FALSE	TRUE
0.00845	FALSE	TRUE
0.003804	FALSE	TRUE
0.209907	TRUE	TRUE
0.005631	TRUE	FALSE
0.40651	FALSE	TRUE
0.006848	FALSE	TRUE
0.007572	FALSE	FALSE
0.002857	FALSE	FALSE
0.008456	FALSE	FALSE
0.030707	FALSE	FALSE
0.00509	FALSE	TRUE
0.003104	FALSE	FALSE

0.004829	FALSE	FALSE
0.027958	FALSE	TRUE
0.041703	FALSE	TRUE
0.031103	FALSE	FALSE
4.40E-05	FALSE	FALSE
9.40E-05	FALSE	FALSE
7.90E-05	FALSE	FALSE
0.002132	FALSE	FALSE
4.71E-04	FALSE	FALSE
9.90E-05	FALSE	FALSE
6.33E-04	FALSE	FALSE
4.90E-05	FALSE	FALSE
0.001934	FALSE	FALSE
5.30E-05	FALSE	FALSE
0.01554	FALSE	FALSE
0.001299	FALSE	FALSE
8.80E-05	FALSE	FALSE
0.021855	FALSE	FALSE
2.47E-04	FALSE	FALSE
0.005127	FALSE	FALSE
9.60E-05	FALSE	FALSE
1.75E-04	FALSE	FALSE
4.61E-04	FALSE	FALSE
7.90E-05	FALSE	FALSE
1.32E-04	FALSE	FALSE
5.20E-04	FALSE	FALSE
0.001715	FALSE	FALSE
9.40E-05	FALSE	FALSE
0.221135	FALSE	TRUE
7.75E-04	FALSE	FALSE
0.001461	FALSE	FALSE
5.54E-04	FALSE	FALSE
6.76E-04	FALSE	FALSE
9.90E-05	FALSE	FALSE
1.02E-04	FALSE	FALSE
5.83E-04	FALSE	FALSE
9.20E-05	FALSE	FALSE
7.44E-04	FALSE	FALSE
1.50E-04	FALSE	FALSE
1.38E-04	FALSE	FALSE
0.167085	FALSE	FALSE
1.63E-04	FALSE	FALSE
9.53E-04	FALSE	FALSE
5.10E-05	FALSE	FALSE
0.098099	FALSE	FALSE
8.10E-05	FALSE	FALSE
3.08E-04	FALSE	FALSE

7.60E-05	FALSE	FALSE
0.01312	FALSE	FALSE
1.15E-04	FALSE	FALSE
0.004567	FALSE	FALSE
0.001156	FALSE	FALSE
6.40E-05	FALSE	FALSE
1.72E-04	FALSE	FALSE
6.84E-04	FALSE	FALSE
2.94E-04	FALSE	FALSE
0.003275	FALSE	FALSE
2.48E-04	FALSE	FALSE
0.001581	FALSE	FALSE
2.54E-04	FALSE	FALSE
0.002206	FALSE	FALSE
0.00115	FALSE	FALSE
2.91E-04	FALSE	FALSE
7.00E-05	FALSE	FALSE
2.79E-04	FALSE	FALSE
8.57E-04	FALSE	FALSE
1.33E-04	FALSE	FALSE
0.001076	FALSE	FALSE
0.001862	FALSE	FALSE
2.51E-04	FALSE	FALSE
8.152442	FALSE	FALSE
1.96E-04	FALSE	FALSE
5.24E-04	FALSE	FALSE
2.27E-04	FALSE	FALSE
0.00436	FALSE	FALSE
0.002027	FALSE	FALSE
0.044117	FALSE	FALSE
0.02443	FALSE	FALSE
8.10E-05	FALSE	FALSE
8.94E-04	FALSE	FALSE
0.002237	FALSE	FALSE
0.007306	FALSE	FALSE
3.55E-04	FALSE	FALSE
0.010391	TRUE	FALSE
1.88E-04	FALSE	FALSE
0.00606	FALSE	FALSE
0.00512	FALSE	FALSE
1.69E-04	FALSE	FALSE
5.80E-05	FALSE	FALSE
6.80E-05	FALSE	FALSE
8.40E-05	FALSE	FALSE
8.50E-05	FALSE	FALSE
0.021523	FALSE	FALSE
8.70E-05	FALSE	FALSE

0.040034	TRUE	FALSE
8.50E-05	FALSE	FALSE
5.61E-04	FALSE	FALSE
0.034171	FALSE	FALSE
1.65E-04	FALSE	FALSE
6.90E-05	FALSE	FALSE
1.63E-04	FALSE	FALSE
3.67E-04	FALSE	FALSE
7.80E-05	FALSE	FALSE
2.46E-04	FALSE	FALSE
2.28E-04	FALSE	FALSE
0.011208	FALSE	FALSE
2.29E-04	FALSE	FALSE
1.15E-04	FALSE	FALSE
2.20E-04	FALSE	FALSE
4.70E-05	FALSE	FALSE
7.20E-05	FALSE	FALSE
1.28E-04	FALSE	FALSE
6.46E-04	FALSE	FALSE
4.41E-04	FALSE	FALSE
0.054319	FALSE	FALSE
1.58E-04	FALSE	FALSE
4.70E-05	FALSE	FALSE
6.10E-05	FALSE	FALSE
0.005066	FALSE	FALSE
4.90E-04	FALSE	FALSE
7.54E-04	FALSE	FALSE
0.003482	FALSE	FALSE
7.30E-05	FALSE	FALSE
8.50E-05	FALSE	FALSE
0.00314	FALSE	FALSE
8.80E-05	FALSE	FALSE
2.43E-04	FALSE	FALSE
3.29E-04	FALSE	FALSE
2.72E-04	FALSE	FALSE
9.40E-05	FALSE	FALSE
0.0014	FALSE	FALSE
9.56E-04	FALSE	FALSE
0.153575	FALSE	TRUE
1.14E-04	FALSE	FALSE
1.41E-04	FALSE	FALSE
7.60E-05	FALSE	FALSE
8.58E-04	FALSE	FALSE
0.224747	FALSE	FALSE
0.009114	FALSE	FALSE
8.30E-05	FALSE	FALSE
6.51E-04	FALSE	FALSE

2.77E-04	FALSE	FALSE
0.001627	FALSE	FALSE
0.001429	FALSE	FALSE
1.64E-04	FALSE	FALSE
9.48E-04	FALSE	FALSE
3.81E-04	FALSE	FALSE
6.70E-05	FALSE	FALSE
0.001219	FALSE	FALSE
7.30E-05	FALSE	FALSE
5.80E-05	FALSE	FALSE
1.31E-04	FALSE	FALSE
4.95E-04	FALSE	FALSE
1.69E-04	FALSE	FALSE
8.50E-05	FALSE	FALSE
4.45E-04	FALSE	FALSE
4.04E-04	FALSE	FALSE
1.09E-04	FALSE	FALSE
4.48E-04	FALSE	FALSE
2.56E-04	FALSE	FALSE
0.003864	FALSE	FALSE
0.014432	FALSE	FALSE
0.010784	FALSE	FALSE
9.90E-05	FALSE	FALSE
0.001067	FALSE	FALSE
6.70E-05	FALSE	FALSE
0.001523	FALSE	FALSE
2.77E-04	FALSE	FALSE
0.020619	FALSE	TRUE
2.42E-04	FALSE	FALSE
4.73E-04	FALSE	FALSE
5.45E-04	FALSE	FALSE
2.08E-04	FALSE	FALSE
2.14E-04	FALSE	FALSE
1.38E-04	FALSE	FALSE
8.88E-04	FALSE	FALSE
3.81E-04	FALSE	FALSE
7.59E-04	FALSE	FALSE
1.31E-04	FALSE	FALSE
6.10E-05	FALSE	FALSE
0.002625	FALSE	FALSE
8.10E-05	FALSE	FALSE
8.20E-05	FALSE	FALSE
9.65E-04	FALSE	FALSE
1.51E-04	FALSE	FALSE
0.002089	FALSE	FALSE
1.69E-04	FALSE	FALSE
1.56E-04	FALSE	FALSE

0.194075	FALSE	FALSE
0.00119	FALSE	FALSE
0.001741	FALSE	FALSE
0.001109	FALSE	FALSE
0.003513	FALSE	FALSE
5.40E-05	FALSE	FALSE
5.72E-04	FALSE	FALSE
5.44E-04	FALSE	FALSE
2.73E-04	FALSE	FALSE
1.80E-04	FALSE	FALSE
7.70E-05	FALSE	FALSE
7.09E-04	FALSE	FALSE
1.79E-04	FALSE	FALSE
2.03E-04	FALSE	FALSE
0.103986	FALSE	FALSE
0.006373	FALSE	FALSE
6.30E-05	FALSE	FALSE
3.67E-04	FALSE	FALSE
8.20E-05	FALSE	FALSE
0.001838	FALSE	FALSE
1.29E-04	FALSE	FALSE
2.77E-04	FALSE	FALSE
0.002449	FALSE	FALSE
7.40E-05	FALSE	FALSE
0.034094	FALSE	TRUE
0.002544	FALSE	FALSE
6.21E-04	FALSE	FALSE
6.30E-05	FALSE	FALSE
7.90E-05	FALSE	FALSE
0.004925	FALSE	FALSE
0.010628	TRUE	FALSE
9.90E-05	FALSE	FALSE
0.003221	FALSE	FALSE
1.01E-04	FALSE	FALSE
0.003561	FALSE	FALSE
2.90E-04	FALSE	FALSE
5.50E-05	FALSE	FALSE
4.12E-04	FALSE	FALSE
3.20E-04	FALSE	FALSE
0.002513	FALSE	FALSE
2.34E-04	FALSE	FALSE
0.001754	FALSE	FALSE
2.657606	FALSE	FALSE
2.19E-04	FALSE	FALSE
0.001352	FALSE	FALSE
8.80E-05	FALSE	FALSE
8.50E-05	FALSE	FALSE

2.94E-04	FALSE	FALSE
2.94E-04	FALSE	FALSE
3.24E-04	FALSE	FALSE
1.09E-04	FALSE	FALSE
2.52E-04	FALSE	FALSE
0.012697	TRUE	FALSE
5.50E-05	FALSE	FALSE
8.90E-05	FALSE	FALSE
1.12E-04	FALSE	FALSE
0.01497	TRUE	FALSE
2.25E-04	FALSE	FALSE
2.70E-04	FALSE	FALSE
0.001628	FALSE	FALSE
1.13E-04	FALSE	FALSE
9.80E-05	FALSE	FALSE
1.26E-04	FALSE	FALSE
2.02E-04	FALSE	FALSE
1.32E-04	FALSE	FALSE
0.142529	FALSE	FALSE
4.94E-04	FALSE	FALSE
2.19E-04	FALSE	FALSE
8.60E-05	FALSE	FALSE
6.90E-05	FALSE	FALSE
7.60E-05	FALSE	FALSE
3.47E-04	FALSE	FALSE
2.34E-04	FALSE	FALSE
0.02458	FALSE	FALSE
4.56E-04	FALSE	FALSE
1.77E-04	FALSE	FALSE
9.90E-05	FALSE	FALSE
2.19E-04	FALSE	FALSE
0.296853	FALSE	TRUE
2.68E-04	FALSE	FALSE
4.28E-04	FALSE	FALSE
5.46E-04	FALSE	FALSE
1.63E-04	FALSE	FALSE
1.25E-04	FALSE	FALSE
4.02E-04	FALSE	FALSE
2.21E-04	FALSE	FALSE
1.11E-04	FALSE	FALSE
1.03E-04	FALSE	FALSE
1.31E-04	FALSE	FALSE
2.15E-04	FALSE	FALSE
4.02E-04	FALSE	FALSE
0.111533	FALSE	FALSE
0.003416	FALSE	FALSE
1.78E-04	FALSE	FALSE

3.09E-04	FALSE	FALSE
0.001425	FALSE	FALSE
0.015905	FALSE	TRUE
0.001772	FALSE	FALSE
5.36E-04	FALSE	FALSE
1.34E-04	FALSE	FALSE
1.01E-04	FALSE	FALSE
0.001409	FALSE	FALSE
4.91E-04	FALSE	FALSE
4.69E-04	FALSE	FALSE
9.00E-05	FALSE	FALSE
2.21E-04	FALSE	FALSE
6.46E-04	FALSE	FALSE
8.70E-05	FALSE	FALSE
0.005232	FALSE	FALSE
8.70E-05	FALSE	FALSE
1.56E-04	FALSE	FALSE
0.01375	FALSE	FALSE
1.27E-04	FALSE	FALSE
6.10E-05	FALSE	FALSE
4.70E-05	FALSE	FALSE
1.96E-04	FALSE	FALSE
0.001152	FALSE	FALSE
1.12E-04	FALSE	FALSE
0.014257	FALSE	FALSE
1.87E-04	FALSE	FALSE
3.33E-04	FALSE	FALSE
1.24E-04	FALSE	FALSE
1.72E-04	FALSE	FALSE
1.00E-04	FALSE	FALSE
0.005378	FALSE	FALSE
5.40E-05	FALSE	FALSE
0.107476	FALSE	FALSE
0.001573	FALSE	FALSE
1.57E-04	FALSE	FALSE
9.20E-05	FALSE	FALSE
1.00E-04	FALSE	FALSE
7.70E-05	FALSE	FALSE
7.40E-05	FALSE	FALSE
2.57E-04	FALSE	FALSE
7.30E-05	FALSE	FALSE
6.30E-05	FALSE	FALSE
0.02486	FALSE	TRUE
6.60E-05	FALSE	FALSE
4.75E-04	FALSE	FALSE
0.023908	TRUE	FALSE
0.001185	FALSE	FALSE

7.30E-05	FALSE	FALSE
1.15E-04	FALSE	FALSE
2.01E-04	FALSE	FALSE
0.005512	FALSE	FALSE
9.26E-04	FALSE	FALSE
3.10E-04	FALSE	FALSE
9.21E-04	FALSE	FALSE
1.21E-04	FALSE	FALSE
0.21619	TRUE	FALSE
9.30E-05	FALSE	FALSE
5.90E-05	FALSE	FALSE
1.87E-04	FALSE	FALSE
0.101434	FALSE	FALSE
2.03E-04	FALSE	FALSE
0.011424	FALSE	FALSE
0.13636	TRUE	FALSE
7.30E-05	FALSE	FALSE
1.24E-04	FALSE	FALSE
1.63E-04	FALSE	FALSE
1.26E-04	FALSE	FALSE
6.60E-05	FALSE	FALSE
0.117167	FALSE	FALSE
0.231347	TRUE	TRUE
5.71E-04	FALSE	FALSE
1.33E-04	FALSE	FALSE
2.87E-04	FALSE	FALSE
0.002992	FALSE	FALSE
0.153273	FALSE	FALSE
3.29E-04	FALSE	FALSE
2.00E-04	FALSE	FALSE
0.033969	FALSE	FALSE
2.24E-04	FALSE	FALSE
0.001165	FALSE	FALSE
0.010566	FALSE	FALSE
1.21E-04	FALSE	FALSE
0.201512	FALSE	FALSE
4.90E-05	FALSE	FALSE
0.09395	TRUE	TRUE
0.022706	TRUE	FALSE
0.021619	FALSE	TRUE
5.80E-05	FALSE	FALSE
2.19E-04	FALSE	FALSE
9.00E-05	FALSE	FALSE
1.99E-04	FALSE	FALSE
1.74E-04	FALSE	FALSE
2.06E-04	FALSE	FALSE
0.001611	FALSE	FALSE

0.006807	FALSE	FALSE
1.33E-04	FALSE	FALSE
9.50E-05	FALSE	FALSE
1.00E-04	FALSE	FALSE
9.60E-05	FALSE	FALSE
1.45E-04	FALSE	FALSE
6.80E-05	FALSE	FALSE
1.64E-04	FALSE	FALSE
3.92E-04	FALSE	FALSE
2.56E-04	FALSE	FALSE
7.70E-05	FALSE	FALSE
0.001282	FALSE	FALSE
1.31E-04	FALSE	FALSE
1.26E-04	FALSE	FALSE
1.61E-04	FALSE	FALSE
7.40E-05	FALSE	FALSE
2.13E-04	FALSE	FALSE
0.004788	FALSE	FALSE
1.70E-04	FALSE	FALSE
9.40E-05	FALSE	FALSE
2.24E-04	FALSE	FALSE
1.65E-04	FALSE	FALSE
0.003153	FALSE	FALSE
0.001372	FALSE	FALSE
0.00258	FALSE	FALSE
3.77E-04	FALSE	FALSE
9.91E-04	FALSE	FALSE
0.001117	FALSE	FALSE
1.20E-04	FALSE	FALSE
9.40E-05	FALSE	FALSE
8.50E-05	FALSE	FALSE
1.04E-04	FALSE	FALSE
9.10E-05	FALSE	FALSE
8.50E-05	FALSE	FALSE
0.002132	FALSE	FALSE
2.51E-04	FALSE	FALSE
1.66E-04	FALSE	FALSE
0.005309	FALSE	FALSE
0.361509	FALSE	FALSE
0.106418	FALSE	FALSE
0.004419	FALSE	FALSE
0.167162	FALSE	FALSE
0.002304	FALSE	FALSE
2.14E-04	FALSE	FALSE
0.004564	FALSE	FALSE
1.01E-04	FALSE	FALSE
0.005637	FALSE	FALSE

0.00723	FALSE	FALSE
0.011484	FALSE	FALSE
0.002418	FALSE	FALSE
0.05354	FALSE	FALSE
1.38E-04	FALSE	FALSE
0.004372	FALSE	FALSE
6.53E-04	FALSE	FALSE
0.003961	FALSE	TRUE
0.062982	TRUE	FALSE
0.001051	FALSE	FALSE
7.05E-04	FALSE	FALSE
0.018895	TRUE	FALSE
7.50E-05	FALSE	FALSE
0.011247	FALSE	FALSE
4.02E-04	FALSE	FALSE
3.29E-04	FALSE	FALSE
1.062347	TRUE	FALSE
0.017729	FALSE	TRUE
3.38E-04	FALSE	FALSE
0.01727	FALSE	FALSE
4.87E-04	FALSE	FALSE
8.10E-05	FALSE	FALSE
3.36E-04	FALSE	FALSE
2.66E-04	FALSE	FALSE
0.056115	FALSE	TRUE
5.53E-04	FALSE	FALSE
1.38E-04	FALSE	FALSE
3.74E-04	FALSE	FALSE
1.46E-04	FALSE	FALSE
1.06E-04	FALSE	FALSE
0.003619	FALSE	FALSE
8.70E-05	FALSE	FALSE
0.032508	FALSE	FALSE
0.001514	FALSE	FALSE
5.54E-04	FALSE	FALSE
7.30E-05	FALSE	FALSE
1.04E-04	FALSE	FALSE
1.99E-04	FALSE	FALSE
1.37E-04	FALSE	FALSE
0.004442	FALSE	FALSE
1.17E-04	FALSE	FALSE
0.279734	FALSE	FALSE
5.91E-04	FALSE	FALSE
0.016065	FALSE	TRUE
3.19E-04	FALSE	FALSE
1.12E-04	FALSE	FALSE
0.022322	FALSE	FALSE

5.59E-04	FALSE	FALSE
0.005876	FALSE	FALSE
0.002822	FALSE	FALSE
1.14E-04	FALSE	FALSE
0.003807	FALSE	FALSE
8.50E-05	FALSE	FALSE
0.007148	FALSE	FALSE
3.73E-04	FALSE	FALSE
4.97E-04	FALSE	FALSE
7.10E-05	FALSE	FALSE
0.062285	FALSE	TRUE
7.90E-05	FALSE	FALSE
1.74E-04	FALSE	FALSE
0.062315	FALSE	TRUE
1.09E-04	FALSE	FALSE
2.74E-04	FALSE	FALSE
0.012285	FALSE	TRUE
0.008927	FALSE	FALSE
0.025962	FALSE	FALSE
1.95E-04	FALSE	FALSE
0.002412	FALSE	FALSE
1.56E-04	FALSE	FALSE
0.722864	FALSE	FALSE
0.02	FALSE	FALSE
2.63E-04	FALSE	FALSE
4.73E-04	FALSE	FALSE
0.004707	FALSE	FALSE
1.13E-04	FALSE	FALSE
0.082324	FALSE	FALSE
1.42E-04	FALSE	FALSE
0.001828	FALSE	FALSE
0.001057	FALSE	FALSE
0.003061	FALSE	FALSE
0.004483	FALSE	FALSE
2.42E-04	FALSE	FALSE
2.04E-04	FALSE	FALSE
0.007466	FALSE	FALSE
1.39E-04	FALSE	FALSE
4.67E-04	FALSE	FALSE
8.70E-05	FALSE	FALSE
0.129409	TRUE	FALSE
2.06E-04	FALSE	FALSE
0.008743	FALSE	FALSE
0.363944	FALSE	TRUE
0.002646	FALSE	FALSE
0.00584	TRUE	FALSE
6.80E-04	FALSE	FALSE

0.003526	FALSE	FALSE
1.37E-04	FALSE	FALSE
0.002557	FALSE	FALSE
0.029358	FALSE	FALSE
0.008049	FALSE	FALSE
0.034164	FALSE	FALSE
1.84E-04	FALSE	FALSE
5.39E-04	FALSE	FALSE
1.37E-04	FALSE	FALSE
4.75E-04	FALSE	FALSE
8.80E-05	FALSE	FALSE
1.26E-04	FALSE	FALSE
0.002113	FALSE	FALSE
1.79E-04	FALSE	FALSE
0.04453	FALSE	TRUE
4.61E-04	FALSE	FALSE
0.004876	FALSE	FALSE
0.004427	FALSE	FALSE
0.017067	FALSE	FALSE
0.062385	FALSE	FALSE
0.010686	FALSE	TRUE
0.005028	FALSE	FALSE
0.132603	TRUE	FALSE
1.65E-04	FALSE	FALSE
1.59E-04	FALSE	FALSE
0.11229	TRUE	TRUE
2.11E-04	FALSE	FALSE
0.00731	FALSE	FALSE
0.002008	FALSE	FALSE
9.20E-05	FALSE	FALSE
9.90E-05	FALSE	FALSE
0.001988	FALSE	FALSE
8.50E-05	FALSE	FALSE
7.00E-05	FALSE	FALSE
1.60E-04	FALSE	FALSE
2.61E-04	FALSE	FALSE
9.91E-04	FALSE	FALSE
2.23E-04	FALSE	FALSE
3.01E-04	FALSE	FALSE
0.233826	TRUE	FALSE
0.024044	FALSE	FALSE
2.98E-04	FALSE	FALSE
1.37E-04	FALSE	FALSE
9.40E-05	FALSE	FALSE
0.003312	FALSE	FALSE
1.13E-04	FALSE	FALSE
0.056319	FALSE	TRUE

1.43E-04	FALSE	FALSE
2.01E-04	FALSE	FALSE
8.50E-05	FALSE	FALSE
8.16E-04	FALSE	FALSE
0.095245	FALSE	FALSE
0.014887	TRUE	FALSE
7.21E-04	FALSE	FALSE
1.55E-04	FALSE	FALSE
0.04501	FALSE	FALSE
0.016293	FALSE	FALSE
0.008326	FALSE	TRUE
1.88E-04	FALSE	FALSE
0.010932	FALSE	FALSE
0.002303	FALSE	FALSE
0.009819	FALSE	FALSE
6.31E-04	FALSE	FALSE
1.98E-04	FALSE	FALSE
1.18E-04	FALSE	FALSE
4.93E-04	FALSE	FALSE
0.005147	FALSE	FALSE
5.21E-04	FALSE	FALSE
0.017624	FALSE	FALSE
7.80E-05	FALSE	FALSE
0.010159	FALSE	FALSE
0.013686	TRUE	TRUE
0.010127	FALSE	TRUE
1.02E-04	FALSE	FALSE
4.65E-04	FALSE	FALSE
0.001631	FALSE	FALSE
0.049784	FALSE	TRUE
1.93E-04	FALSE	FALSE
0.006957	FALSE	FALSE
0.000649	FALSE	FALSE
0.011525	FALSE	FALSE
1.24E-04	FALSE	FALSE
0.022279	FALSE	FALSE
0.007077	FALSE	FALSE
2.13E-04	FALSE	FALSE
8.30E-05	FALSE	FALSE
3.20E-04	FALSE	FALSE
6.21E-04	FALSE	FALSE
0.004499	FALSE	FALSE
1.78E-04	FALSE	FALSE
0.026719	FALSE	FALSE
8.90E-05	FALSE	FALSE
0.004606	FALSE	FALSE
0.002857	FALSE	FALSE

4.51E-04	FALSE	FALSE
1.63E-04	FALSE	FALSE
1.03E-04	FALSE	FALSE
1.82E-04	FALSE	FALSE
0.009586	TRUE	FALSE
0.117796	FALSE	FALSE
0.005682	FALSE	FALSE
0.001545	FALSE	FALSE
2.17E-04	FALSE	FALSE
0.01419	FALSE	FALSE
0.107512	TRUE	TRUE
2.34E-04	FALSE	FALSE
3.94E-04	FALSE	FALSE
1.22E-04	FALSE	FALSE
1.94E-04	FALSE	FALSE
1.64E-04	FALSE	FALSE
0.007771	FALSE	TRUE
4.29E-04	FALSE	FALSE
0.009075	FALSE	FALSE
9.04E-04	FALSE	FALSE
2.29E-04	FALSE	FALSE
3.80E-04	FALSE	FALSE
2.35E-04	FALSE	FALSE
0.002372	FALSE	FALSE
9.36E-04	FALSE	FALSE
2.19E-04	FALSE	FALSE
1.73E-04	FALSE	FALSE
0.007007	FALSE	FALSE
0.026585	FALSE	TRUE
7.60E-05	FALSE	FALSE
2.18E-04	FALSE	FALSE
0.005352	FALSE	FALSE
0.185321	FALSE	FALSE
2.20E-04	FALSE	FALSE
1.85E-04	FALSE	FALSE
0.007353	FALSE	FALSE
5.90E-05	FALSE	FALSE
2.89E-04	FALSE	FALSE
0.002132	FALSE	FALSE
4.47E-04	FALSE	FALSE
0.008637	FALSE	TRUE
6.65E-04	FALSE	FALSE
0.014299	FALSE	FALSE
2.49E-04	FALSE	FALSE
0.068992	FALSE	FALSE
0.00695	FALSE	FALSE
7.40E-05	FALSE	FALSE

0.0034	FALSE	FALSE
0.021241	FALSE	TRUE
6.31E-04	FALSE	FALSE
0.002087	FALSE	FALSE
2.61E-04	FALSE	FALSE
0.193617	FALSE	FALSE
1.33E-04	FALSE	FALSE
1.69E-04	FALSE	FALSE
5.47E-04	FALSE	FALSE
0.005643	FALSE	FALSE
3.69E-04	FALSE	FALSE
44.261596	FALSE	FALSE
0.016001	FALSE	TRUE
0.017368	FALSE	TRUE
0.001289	FALSE	FALSE
1.12E-04	FALSE	FALSE
1.44E-04	FALSE	FALSE
7.10E-05	FALSE	FALSE
7.90E-05	FALSE	FALSE
0.003455	FALSE	FALSE
9.58E-04	FALSE	FALSE
9.40E-04	FALSE	FALSE
4.60E-05	FALSE	FALSE
0.002076	FALSE	FALSE
0.008256	FALSE	FALSE
1.09E-04	FALSE	FALSE
0.016596	FALSE	FALSE
0.00623	FALSE	FALSE
0.007623	FALSE	FALSE
2.61E-04	FALSE	FALSE
1.98E-04	FALSE	FALSE
0.058109	FALSE	FALSE
1.50E-04	FALSE	FALSE
0.006205	FALSE	FALSE
7.34E-04	FALSE	FALSE
0.005385	FALSE	FALSE
0.025029	FALSE	FALSE
0.01485	FALSE	FALSE
0.001007	FALSE	FALSE
8.05E-04	FALSE	FALSE
0.016233	FALSE	FALSE
0.002817	FALSE	FALSE
0.009525	FALSE	FALSE
7.46E-04	FALSE	FALSE
7.98E-04	FALSE	FALSE
6.11E-04	FALSE	FALSE
9.90E-04	FALSE	FALSE

0.008567	FALSE	FALSE
0.002421	FALSE	FALSE
0.002204	TRUE	FALSE
7.21E-04	FALSE	FALSE
0.016977	FALSE	FALSE
0.002368	FALSE	FALSE
9.41E-04	FALSE	FALSE
6.28E-04	FALSE	FALSE
6.03E-04	FALSE	FALSE
7.02E-04	FALSE	FALSE
4.52E-04	FALSE	FALSE
0.001259	FALSE	FALSE
6.46E-04	FALSE	FALSE
0.002511	FALSE	FALSE
0.002202	FALSE	FALSE
0.001579	FALSE	FALSE
0.020162	FALSE	FALSE
0.002404	FALSE	FALSE
0.001836	FALSE	FALSE
2.635473	FALSE	FALSE
0.011651	FALSE	FALSE
0.001497	FALSE	FALSE
0.001088	FALSE	FALSE
7.19E-04	FALSE	FALSE
4.820462	FALSE	TRUE
0.002379	FALSE	FALSE
0.106946	FALSE	FALSE
0.001519	FALSE	FALSE
0.003791	FALSE	FALSE
9.64E-04	FALSE	FALSE
7.77E-04	FALSE	FALSE
0.029533	FALSE	FALSE
0.001752	FALSE	FALSE
0.01515	FALSE	TRUE
0.011506	FALSE	TRUE
0.002978	FALSE	FALSE
0.032415	FALSE	FALSE
0.001376	FALSE	FALSE
7.33E-04	FALSE	FALSE
0.02957	FALSE	FALSE
5.41E-04	FALSE	FALSE
6.69E-04	FALSE	FALSE
0.006996	FALSE	FALSE
9.08E-04	FALSE	FALSE
0.043478	FALSE	FALSE
0.023395	FALSE	TRUE
3.01E-04	FALSE	FALSE

0.00879	FALSE	TRUE
0.004926	FALSE	FALSE
0.081305	FALSE	FALSE
0.005877	FALSE	FALSE
0.001906	FALSE	FALSE
0.004114	TRUE	FALSE
0.001362	FALSE	FALSE
0.009625	FALSE	TRUE
0.101442	FALSE	TRUE
0.001675	FALSE	FALSE
0.001503	FALSE	FALSE
2.643644	FALSE	TRUE
0.203161	TRUE	TRUE
5.64E-04	FALSE	FALSE
0.014214	FALSE	FALSE
2.573667	FALSE	FALSE
0.001473	FALSE	FALSE
0.207903	FALSE	FALSE
6.27E-04	FALSE	FALSE
7.55E-04	FALSE	FALSE
0.016824	FALSE	FALSE
0.002093	FALSE	FALSE
0.001149	FALSE	FALSE
8.81E-04	FALSE	FALSE
0.084301	FALSE	FALSE
8.27E-04	FALSE	FALSE
0.027612	FALSE	FALSE
4.51E-04	FALSE	FALSE
0.00123	FALSE	FALSE
8.73E-04	FALSE	FALSE
0.021746	FALSE	FALSE
0.01373	FALSE	FALSE
0.001454	FALSE	FALSE
0.074324	FALSE	FALSE
0.116161	FALSE	FALSE
7.01E-04	FALSE	FALSE
0.007463	FALSE	FALSE
0.026349	FALSE	FALSE
1.063284	FALSE	FALSE
0.002384	FALSE	FALSE
0.031969	TRUE	FALSE
0.004265	FALSE	FALSE
8.36E-04	TRUE	FALSE
0.002734	FALSE	FALSE
0.006325	FALSE	FALSE
4.60E-04	FALSE	FALSE
8.18E-04	FALSE	FALSE

0.001159	FALSE	FALSE
0.002224	FALSE	FALSE
0.001428	FALSE	FALSE
9.44E-04	FALSE	FALSE
0.246297	FALSE	TRUE
0.001318	FALSE	FALSE
0.004501	FALSE	TRUE
0.160363	TRUE	TRUE
3.48E-04	FALSE	FALSE
7.93E-04	FALSE	FALSE
0.262946	TRUE	FALSE
0.00129	FALSE	FALSE
0.058107	FALSE	FALSE
3.72E-04	FALSE	FALSE
0.019078	FALSE	FALSE
0.002155	FALSE	FALSE
0.002569	FALSE	FALSE
0.001392	FALSE	FALSE
0.002896	FALSE	FALSE
1.368336	FALSE	FALSE
7.21E-04	FALSE	FALSE
0.09553	TRUE	TRUE
0.029291	TRUE	FALSE
3.385816	FALSE	FALSE
8.11E-04	FALSE	FALSE
0.00103	FALSE	FALSE
7.55E-04	FALSE	FALSE
0.038421	TRUE	FALSE
0.082998	FALSE	FALSE
0.010179	FALSE	FALSE
0.005065	FALSE	FALSE
0.004709	FALSE	FALSE
0.03456	FALSE	FALSE
4.84E-04	FALSE	FALSE
0.001853	FALSE	FALSE
5.69E-04	FALSE	FALSE
0.063083	FALSE	FALSE
0.011219	FALSE	FALSE
0.065563	FALSE	TRUE
0.005375	FALSE	FALSE
0.001051	FALSE	FALSE
0.156981	FALSE	FALSE
0.012201	FALSE	TRUE
0.020088	FALSE	FALSE
9.01E-04	FALSE	FALSE
0.003797	FALSE	FALSE
5.99E-04	FALSE	FALSE

5.68E-04	FALSE	FALSE
0.002253	FALSE	FALSE
0.01076	FALSE	FALSE
7.08E-04	FALSE	FALSE
0.001069	FALSE	FALSE
0.008006	FALSE	FALSE
5.63E-04	FALSE	FALSE
0.002097	FALSE	FALSE
0.109333	FALSE	FALSE
0.004911	FALSE	FALSE
0.012244	FALSE	FALSE
6.20E-04	FALSE	FALSE
7.67E-04	FALSE	FALSE
0.005162	TRUE	FALSE
0.00128	FALSE	FALSE
0.069587	FALSE	FALSE
0.007736	FALSE	FALSE
0.075313	FALSE	FALSE
0.008641	FALSE	FALSE
0.005021	FALSE	FALSE
0.002048	FALSE	FALSE
8.65E-04	FALSE	FALSE
0.006828	TRUE	TRUE
0.017025	FALSE	FALSE
0.03735	FALSE	FALSE
0.001614	FALSE	FALSE
5.17E-04	FALSE	FALSE
0.01744	FALSE	TRUE
0.12948	FALSE	TRUE
0.001049	FALSE	FALSE
0.00857	FALSE	FALSE
0.046102	FALSE	FALSE
6.90E-04	FALSE	FALSE
0.019197	FALSE	FALSE
0.031566	TRUE	FALSE
4.76E-04	FALSE	FALSE
5.40E-04	FALSE	FALSE
0.002099	TRUE	TRUE
8.58E-04	FALSE	FALSE
0.015966	FALSE	FALSE
0.015487	TRUE	FALSE
7.24E-04	FALSE	FALSE
0.09734	FALSE	FALSE
0.009339	FALSE	TRUE
0.001613	FALSE	TRUE
0.001749	FALSE	FALSE
0.00298	FALSE	FALSE

0.001318	FALSE	FALSE
0.018168	FALSE	FALSE
5.80E-04	FALSE	FALSE
7.20E-04	FALSE	FALSE
0.004798	FALSE	FALSE
0.011029	FALSE	FALSE
9.59E-04	FALSE	TRUE
0.017909	TRUE	FALSE
0.00607	FALSE	FALSE
0.008479	FALSE	FALSE
0.001245	FALSE	FALSE
0.053057	FALSE	FALSE
0.003289	TRUE	FALSE
0.001628	FALSE	FALSE
0.003009	FALSE	FALSE
0.181995	FALSE	FALSE
4.85E-04	FALSE	FALSE
0.024673	FALSE	FALSE
0.008006	FALSE	FALSE
6.87E-04	FALSE	FALSE
0.202209	FALSE	TRUE
0.002685	FALSE	TRUE
0.056689	FALSE	FALSE
4.28E-04	FALSE	FALSE
0.03497	FALSE	FALSE
0.003567	FALSE	FALSE
0.087653	FALSE	FALSE
0.002124	TRUE	FALSE
0.004104	FALSE	FALSE
0.158239	FALSE	FALSE
0.001441	FALSE	FALSE
0.018858	FALSE	FALSE
0.001269	FALSE	FALSE
0.017132	FALSE	FALSE
0.002092	FALSE	FALSE
0.00402	FALSE	FALSE
7.02E-04	FALSE	FALSE
0.080846	FALSE	FALSE
4.98E-04	FALSE	FALSE
0.033991	FALSE	FALSE
0.009284	TRUE	TRUE
8.63E-04	FALSE	FALSE
8.35E-04	FALSE	FALSE
0.037505	FALSE	FALSE
0.177952	FALSE	FALSE
0.027722	FALSE	FALSE
3.75E-04	FALSE	FALSE

5.87E-04	FALSE	FALSE
0.012385	FALSE	FALSE
6.36E-04	FALSE	TRUE
0.002022	FALSE	FALSE
6.11E-04	FALSE	FALSE
0.001675	FALSE	FALSE
9.19E-04	FALSE	FALSE
0.006146	FALSE	FALSE
0.00256	FALSE	TRUE
8.59E-04	FALSE	FALSE
0.004992	FALSE	TRUE
0.014389	FALSE	FALSE
0.010212	FALSE	FALSE
0.089841	FALSE	FALSE
0.001386	FALSE	FALSE
5.30E-04	FALSE	FALSE
0.137985	TRUE	FALSE
0.002021	FALSE	FALSE
6.37E-04	FALSE	FALSE
0.007151	FALSE	FALSE
0.010475	FALSE	FALSE
0.001839	FALSE	FALSE
0.001641	FALSE	FALSE
0.059955	FALSE	TRUE
0.006606	FALSE	TRUE
0.019126	TRUE	TRUE
0.060407	FALSE	TRUE
8.43E-04	FALSE	FALSE
8.36E-04	FALSE	FALSE
0.008343	FALSE	FALSE
0.006393	FALSE	FALSE
0.002087	FALSE	FALSE
0.015076	FALSE	TRUE
0.010495	FALSE	TRUE
0.063242	FALSE	TRUE
0.057534	FALSE	FALSE
0.004441	FALSE	FALSE
0.022124	FALSE	TRUE
0.043392	FALSE	FALSE
0.010863	FALSE	FALSE
0.033756	FALSE	TRUE
0.004625	FALSE	FALSE
0.009994	FALSE	FALSE
0.039507	FALSE	FALSE
0.001884	FALSE	TRUE
7.01E-04	FALSE	FALSE
5.13E-04	FALSE	FALSE

0.049697	FALSE	FALSE
4.06E-04	FALSE	FALSE
9.48E-04	FALSE	FALSE
4.23E-04	FALSE	FALSE
6.19E-04	FALSE	FALSE
0.008434	FALSE	TRUE
7.36E-04	FALSE	FALSE
0.00114	FALSE	FALSE
4.15E-04	FALSE	FALSE
3.55E-04	FALSE	FALSE
0.00132	FALSE	FALSE
0.002773	FALSE	FALSE
0.04066	FALSE	TRUE
0.001247	FALSE	FALSE
0.012254	FALSE	FALSE
0.004619	FALSE	FALSE
0.002154	FALSE	FALSE
0.004085	FALSE	FALSE
0.019058	FALSE	FALSE
5.28E-04	FALSE	FALSE
0.45538	FALSE	FALSE
0.094172	FALSE	FALSE
0.150765	TRUE	FALSE
7.90E-04	FALSE	FALSE
5.91E-04	FALSE	FALSE
0.001018	FALSE	FALSE
0.007269	TRUE	TRUE
4.15E-04	FALSE	FALSE
0.001045	TRUE	FALSE
0.002352	FALSE	FALSE
5.01E-04	FALSE	FALSE
4.07E-04	FALSE	FALSE
0.18809	FALSE	FALSE
4.93E-04	FALSE	FALSE
5.34E-04	FALSE	FALSE
5.99E-04	FALSE	FALSE
0.006998	FALSE	FALSE
0.143747	FALSE	FALSE
0.002534	FALSE	FALSE
0.010456	FALSE	TRUE
5.13E-04	FALSE	FALSE
0.006133	FALSE	FALSE
0.049928	FALSE	FALSE
0.043179	TRUE	TRUE
3.02E-04	FALSE	FALSE
0.00124	FALSE	FALSE
5.16E-04	FALSE	FALSE

4.06E-04	FALSE	FALSE
0.001032	FALSE	FALSE
3.77E-04	FALSE	FALSE
0.023728	FALSE	FALSE
0.013423	FALSE	FALSE
0.635368	FALSE	FALSE
4.67E-04	FALSE	FALSE
6.50E-04	FALSE	FALSE
0.140744	FALSE	TRUE
0.030812	FALSE	TRUE
0.00108	FALSE	FALSE
0.001236	FALSE	FALSE
6.43E-04	FALSE	FALSE
0.003012	FALSE	FALSE
0.002576	TRUE	TRUE
0.001304	FALSE	FALSE
0.016589	FALSE	FALSE
0.009619	FALSE	FALSE
0.005919	FALSE	FALSE
4.93E-04	FALSE	FALSE
0.078348	FALSE	FALSE
0.060133	TRUE	FALSE
0.001056	FALSE	FALSE
3.85E-04	FALSE	FALSE
0.030629	FALSE	FALSE
0.030499	FALSE	FALSE
0.001155	FALSE	FALSE
0.014266	FALSE	FALSE
0.002157	FALSE	FALSE
0.005104	FALSE	FALSE
0.015882	FALSE	FALSE
0.020828	FALSE	FALSE
7.61E-04	FALSE	FALSE
0.001119	FALSE	FALSE
0.142931	FALSE	FALSE
0.192072	FALSE	FALSE
0.001656	FALSE	FALSE
0.013311	FALSE	FALSE
0.011071	TRUE	FALSE
0.066696	FALSE	FALSE
0.019057	FALSE	FALSE
0.002184	FALSE	FALSE
0.031851	FALSE	FALSE
5.12E-04	FALSE	FALSE
0.108538	FALSE	FALSE
4.05E-04	FALSE	FALSE
5.15E-04	FALSE	FALSE

0.00299	FALSE	FALSE
0.001528	TRUE	FALSE
5.00E-04	FALSE	FALSE
0.002731	FALSE	FALSE
0.005109	FALSE	FALSE
0.073602	FALSE	FALSE
9.38E-04	FALSE	FALSE
0.00257	FALSE	FALSE
0.00181	FALSE	FALSE
0.001737	FALSE	FALSE
0.005065	FALSE	FALSE
4.68E-04	FALSE	FALSE
3.84E-04	FALSE	FALSE
0.054096	FALSE	FALSE
0.002299	FALSE	FALSE
0.148294	FALSE	FALSE
0.001467	FALSE	FALSE
5.78E-04	FALSE	FALSE
2.33E-04	FALSE	FALSE
0.006044	FALSE	FALSE
0.026338	TRUE	TRUE
0.017488	FALSE	FALSE
4.01E-04	FALSE	FALSE
9.20E-04	FALSE	TRUE
4.37E-04	FALSE	FALSE
0.007805	FALSE	FALSE
0.047269	FALSE	FALSE
0.037897	FALSE	FALSE
0.005246	FALSE	FALSE
4.04E-04	FALSE	FALSE
0.001005	FALSE	FALSE
0.03693	FALSE	FALSE
0.001433	FALSE	FALSE
8.28E-04	FALSE	FALSE
4.87E-04	FALSE	FALSE
0.011819	FALSE	FALSE
0.002933	FALSE	FALSE
8.63E-04	FALSE	FALSE
0.001032	FALSE	FALSE
3.95E-04	FALSE	FALSE
6.47E-04	FALSE	FALSE
0.012654	FALSE	FALSE
4.71E-04	FALSE	FALSE
6.44E-04	FALSE	FALSE
4.98E-04	FALSE	FALSE
8.25E-04	FALSE	FALSE
9.09E-04	FALSE	FALSE

0.001737	FALSE	TRUE
0.016	FALSE	FALSE
0.004231	TRUE	FALSE
0.129529	FALSE	FALSE
0.017035	FALSE	FALSE
0.01934	TRUE	FALSE
0.004357	FALSE	FALSE
2.96E-04	FALSE	FALSE
0.055223	FALSE	FALSE
0.024936	FALSE	FALSE
0.020401	FALSE	TRUE
0.030908	FALSE	TRUE
9.64E-04	FALSE	FALSE
5.65E-04	FALSE	FALSE
0.212547	FALSE	FALSE
7.96E-04	FALSE	FALSE
5.04E-04	FALSE	FALSE
0.001192	FALSE	FALSE
0.052773	TRUE	FALSE
0.005003	FALSE	FALSE
3.04E-04	FALSE	FALSE
3.28E-04	FALSE	FALSE
0.001311	FALSE	FALSE
0.002044	FALSE	FALSE
0.030341	TRUE	FALSE
0.001021	FALSE	FALSE
4.87E-04	FALSE	FALSE
3.52E-04	FALSE	FALSE
0.00145	FALSE	FALSE
3.69E-04	FALSE	FALSE
0.045878	FALSE	FALSE
0.00771	FALSE	FALSE
0.002327	FALSE	FALSE
9.02E-04	FALSE	FALSE
0.054832	FALSE	FALSE
5.84E-04	FALSE	FALSE
15.841235	FALSE	FALSE
0.115278	FALSE	FALSE
3.34E-04	FALSE	FALSE
0.075101	FALSE	TRUE
0.001198	FALSE	FALSE
4.51E-04	FALSE	FALSE
0.050826	FALSE	FALSE
0.006829	FALSE	FALSE
3.99E-04	FALSE	FALSE
0.033835	FALSE	FALSE
0.202778	FALSE	FALSE

0.014228	FALSE	FALSE
0.04434	TRUE	FALSE
0.003453	FALSE	FALSE
4.47E-04	FALSE	FALSE
5.00E-04	FALSE	FALSE
3.30E-04	FALSE	FALSE
4.29E-04	FALSE	FALSE
0.048873	FALSE	FALSE
6.11E-04	FALSE	FALSE
0.004566	FALSE	FALSE
0.010753	FALSE	TRUE
0.059514	FALSE	FALSE
0.14343	FALSE	TRUE
0.001015	FALSE	FALSE
0.007472	FALSE	TRUE
0.023342	FALSE	FALSE
8.69E-04	FALSE	FALSE
0.001387	FALSE	FALSE
8.23E-04	FALSE	FALSE
4.92E-04	FALSE	FALSE
0.020221	FALSE	FALSE
0.005749	FALSE	FALSE
0.027852	FALSE	FALSE
4.56E-04	FALSE	FALSE
0.012998	FALSE	FALSE
8.22E-04	FALSE	FALSE
0.073435	FALSE	FALSE
5.42E-04	FALSE	FALSE
4.13E-04	FALSE	FALSE
8.22E-04	FALSE	FALSE
0.00193	FALSE	FALSE
3.73E-04	FALSE	FALSE
0.001703	FALSE	FALSE
0.017011	FALSE	TRUE
0.005395	FALSE	FALSE
8.78E-04	FALSE	FALSE
0.005945	FALSE	TRUE
0.011376	FALSE	FALSE
3.32E-04	FALSE	FALSE
0.001693	FALSE	FALSE
7.23E-04	FALSE	FALSE
0.001299	FALSE	FALSE
0.007057	FALSE	FALSE
2.071408	FALSE	FALSE
0.001538	FALSE	FALSE
0.003149	FALSE	FALSE
0.002462	FALSE	FALSE

4.17E-04	FALSE	FALSE
3.64E-04	FALSE	FALSE
0.00242	FALSE	FALSE
5.55E-04	FALSE	FALSE
4.42E-04	TRUE	FALSE
0.825654	TRUE	FALSE
5.10E-04	FALSE	FALSE
0.003039	FALSE	FALSE
6.43E-04	FALSE	FALSE
7.08E-04	FALSE	FALSE
5.48E-04	FALSE	FALSE
0.003592	FALSE	FALSE
5.80E-04	FALSE	FALSE
6.02E-04	FALSE	FALSE
3.73E-04	FALSE	FALSE
0.002387	FALSE	FALSE
7.09E-04	FALSE	FALSE
0.002055	FALSE	FALSE
4.95E-04	FALSE	FALSE
0.001714	FALSE	FALSE
0.018249	FALSE	FALSE
3.14E-04	FALSE	FALSE
7.10E-04	FALSE	FALSE
0.002669	FALSE	TRUE
0.008025	FALSE	FALSE
4.16E-04	FALSE	FALSE
6.85E-04	FALSE	FALSE
0.002542	FALSE	FALSE
5.76E-04	FALSE	FALSE
7.13E-04	FALSE	FALSE
0.002246	FALSE	FALSE
0.14808	FALSE	FALSE
9.67E-04	FALSE	FALSE
6.85E-04	FALSE	FALSE
3.04E-04	FALSE	FALSE
0.008412	FALSE	FALSE
0.003103	FALSE	FALSE
0.002143	FALSE	FALSE
0.00154	FALSE	FALSE
0.028652	FALSE	FALSE
0.001628	FALSE	FALSE
0.592239	FALSE	FALSE
0.215801	FALSE	FALSE
0.028933	FALSE	FALSE
0.001345	FALSE	FALSE
0.01707	FALSE	FALSE
5.88E-04	FALSE	FALSE

0.01599	FALSE	FALSE
0.764713	FALSE	FALSE
0.005373	FALSE	FALSE
0.02277	TRUE	TRUE
0.008093	FALSE	FALSE
0.006076	FALSE	FALSE
0.001146	FALSE	FALSE
0.001339	FALSE	FALSE
0.053386	FALSE	FALSE
4.51E-04	FALSE	FALSE
6.13E-04	FALSE	FALSE
0.008006	FALSE	FALSE
0.014424	FALSE	FALSE
0.004423	TRUE	FALSE
7.67E-04	FALSE	FALSE
0.00609	FALSE	TRUE
5.96E-04	FALSE	FALSE
0.059345	FALSE	TRUE
0.001	FALSE	TRUE
0.004327	FALSE	FALSE
0.002273	FALSE	FALSE
9.78E-04	FALSE	FALSE
8.28E-04	FALSE	FALSE
0.021332	FALSE	TRUE
0.00211	FALSE	FALSE
0.001416	FALSE	FALSE
0.001057	FALSE	FALSE
0.024159	FALSE	FALSE
4.52E-04	FALSE	FALSE
0.052873	FALSE	TRUE
0.236283	FALSE	FALSE
0.002235	FALSE	FALSE
0.155634	FALSE	TRUE
0.002837	FALSE	FALSE
0.007551	FALSE	FALSE
7.50E-04	FALSE	FALSE
0.055432	FALSE	FALSE
9.08E-04	FALSE	FALSE
0.004433	FALSE	TRUE
0.007942	FALSE	FALSE
0.018451	FALSE	TRUE
8.58E-04	FALSE	FALSE
5.53E-04	FALSE	FALSE
0.001067	FALSE	FALSE
0.147188	FALSE	TRUE
0.003545	FALSE	FALSE
0.067831	FALSE	FALSE

5.53E-04	FALSE	FALSE
0.001634	TRUE	FALSE
5.07E-04	FALSE	FALSE
1.57E-04	FALSE	FALSE
0.00131	FALSE	FALSE
2.22E-04	FALSE	FALSE
0.501339	FALSE	FALSE
0.001371	FALSE	FALSE
2.19E-04	FALSE	FALSE
0.010341	FALSE	FALSE
0.0069	FALSE	TRUE
0.069553	FALSE	FALSE
0.005503	FALSE	FALSE
1.22E-04	FALSE	FALSE
0.141732	FALSE	FALSE
0.005801	FALSE	FALSE
1.54E-04	FALSE	FALSE
2.77E-04	FALSE	FALSE
0.056845	FALSE	TRUE
0.011176	FALSE	FALSE
0.002475	TRUE	FALSE
0.01253	FALSE	FALSE
0.001571	FALSE	FALSE
0.039129	FALSE	FALSE
2.62E-04	FALSE	FALSE
0.001869	FALSE	FALSE
0.058106	FALSE	FALSE
0.001891	TRUE	FALSE
2.04E-04	FALSE	FALSE
5.19E-04	FALSE	FALSE
0.001321	FALSE	FALSE
0.085296	TRUE	FALSE
0.001431	FALSE	FALSE
0.086975	FALSE	FALSE
0.015405	FALSE	FALSE
6.43E-04	FALSE	FALSE
1.866475	FALSE	FALSE
0.001068	FALSE	FALSE
4.07E-04	FALSE	FALSE
0.219098	FALSE	FALSE
4.41E-04	FALSE	FALSE
0.001262	FALSE	FALSE
0.046464	FALSE	FALSE
0.002333	FALSE	FALSE
1.82E-04	FALSE	FALSE
5.65E-04	FALSE	FALSE
3.11E-04	FALSE	FALSE

2.23E-04	FALSE	FALSE
0.001355	FALSE	FALSE
0.002563	FALSE	FALSE
1.80E-04	FALSE	FALSE
0.004393	FALSE	TRUE
0.017147	FALSE	FALSE
2.59E-04	FALSE	FALSE
1.52E-04	FALSE	FALSE
5.07E-04	FALSE	FALSE
1.59E-04	FALSE	FALSE
0.001722	FALSE	FALSE
0.035774	FALSE	FALSE
0.00222	FALSE	FALSE
0.014114	FALSE	FALSE
2.24E-04	FALSE	FALSE
2.19E-04	FALSE	FALSE
0.054507	TRUE	TRUE
5.37E-04	FALSE	FALSE
8.08E-04	FALSE	FALSE
0.007059	FALSE	FALSE
0.003746	FALSE	FALSE
0.06803	TRUE	FALSE
6.844116	FALSE	TRUE
0.019298	FALSE	FALSE
0.034695	FALSE	FALSE
1.28E-04	FALSE	FALSE
3.15E-04	FALSE	FALSE
0.001173	FALSE	FALSE
0.006759	FALSE	FALSE
0.008516	FALSE	FALSE
0.001078	FALSE	FALSE
1.31E-04	FALSE	FALSE
8.41E-04	FALSE	FALSE
1.32E-04	FALSE	FALSE
2.77E-04	FALSE	FALSE
2.64E-04	FALSE	FALSE
2.18E-04	FALSE	FALSE
0.009627	TRUE	FALSE
2.97E-04	FALSE	FALSE
1.66E-04	FALSE	FALSE
0.006648	FALSE	TRUE
0.001098	FALSE	FALSE
2.42E-04	FALSE	FALSE
1.95E-04	FALSE	FALSE
1.15E-04	FALSE	FALSE
1.134178	FALSE	FALSE
2.65E-04	FALSE	FALSE

0.011556	FALSE	TRUE
7.20E-04	FALSE	FALSE
0.040104	FALSE	FALSE
1.45E-04	FALSE	FALSE
3.50E-04	FALSE	FALSE
0.012467	FALSE	FALSE
1.88E-04	FALSE	FALSE
1.14E-04	FALSE	FALSE
0.001211	FALSE	FALSE
3.14E-04	FALSE	FALSE
2.36E-04	FALSE	FALSE
0.05607	TRUE	FALSE
0.002146	FALSE	FALSE
9.10E-05	FALSE	FALSE
1.58E-04	FALSE	FALSE
3.43E-04	FALSE	FALSE
1.08E-04	FALSE	FALSE
3.44E-04	FALSE	FALSE
2.67E-04	FALSE	FALSE
1.92E-04	FALSE	FALSE
1.60E-04	FALSE	FALSE
0.027901	FALSE	FALSE
1.87E-04	FALSE	FALSE
0.003133	FALSE	FALSE
0.073228	FALSE	FALSE
0.061048	TRUE	FALSE
0.062303	FALSE	FALSE
0.072205	TRUE	FALSE
0.019592	FALSE	FALSE
2.20E-04	FALSE	FALSE
0.007071	FALSE	TRUE
3.09E-04	FALSE	FALSE
8.59E-04	FALSE	FALSE
0.070857	TRUE	FALSE
0.006586	FALSE	TRUE
5.92E-04	FALSE	FALSE
1.09E-04	FALSE	FALSE
0.003043	FALSE	FALSE
0.947403	FALSE	FALSE
0.026175	FALSE	FALSE
0.001717	FALSE	FALSE
0.071436	TRUE	FALSE
0.017556	FALSE	FALSE
3.26E-04	FALSE	FALSE
0.079661	TRUE	TRUE
2.14E-04	FALSE	FALSE
1.06E-04	FALSE	FALSE

2.57E-04	FALSE	FALSE
0.03934	FALSE	FALSE
0.002082	FALSE	FALSE
0.009992	TRUE	TRUE
3.33E-04	FALSE	FALSE
3.60E-04	FALSE	FALSE
0.003128	FALSE	FALSE
0.003206	TRUE	FALSE
0.005251	TRUE	FALSE
2.34E-04	FALSE	FALSE
8.86E-04	FALSE	FALSE
0.078196	FALSE	FALSE
6.35E-04	FALSE	FALSE
0.058674	FALSE	FALSE
0.007801	FALSE	FALSE
0.001535	FALSE	FALSE
0.005097	FALSE	TRUE
0.00566	FALSE	FALSE
0.003219	FALSE	FALSE
3.60E-04	FALSE	FALSE
0.001997	FALSE	FALSE
0.00325	FALSE	FALSE
1.23E-04	FALSE	FALSE
0.001378	FALSE	FALSE
1.95E-04	FALSE	FALSE
0.001475	FALSE	FALSE
0.001491	FALSE	FALSE
4.73E-04	FALSE	FALSE
0.011133	FALSE	FALSE
1.96E-04	FALSE	FALSE
0.0011	FALSE	FALSE
5.09E-04	FALSE	FALSE
0.003691	FALSE	FALSE
4.64E-04	FALSE	FALSE
0.004612	FALSE	FALSE
5.51E-04	FALSE	FALSE
0.00341	FALSE	FALSE
0.016094	FALSE	FALSE
9.62E-04	FALSE	FALSE
6.19E-04	FALSE	FALSE
0.002469	FALSE	FALSE
2.368389	FALSE	TRUE
7.49E-04	FALSE	FALSE
0.003631	FALSE	FALSE
0.101488	FALSE	FALSE
4.84E-04	FALSE	FALSE
0.003471	FALSE	FALSE

3.39E-04	FALSE	FALSE
2.92E-04	FALSE	FALSE
0.001046	FALSE	FALSE
9.96E-04	FALSE	FALSE
5.56E-04	FALSE	FALSE
0.002529	FALSE	FALSE
5.87E-04	FALSE	FALSE
1.80E-04	FALSE	FALSE
3.77E-04	FALSE	FALSE
1.20E-04	FALSE	FALSE
2.12E-04	FALSE	FALSE
3.61E-04	FALSE	FALSE
0.003474	FALSE	FALSE
0.004015	FALSE	TRUE
0.042658	FALSE	FALSE
6.65E-04	FALSE	FALSE
0.001314	FALSE	FALSE
1.52E-04	FALSE	FALSE
2.75E-04	FALSE	FALSE
6.71E-04	FALSE	FALSE
2.28E-04	FALSE	FALSE
0.001776	FALSE	FALSE
8.60E-05	FALSE	FALSE
4.13E-04	FALSE	FALSE
2.01E-04	FALSE	FALSE
1.05E-04	FALSE	FALSE
1.55E-04	FALSE	FALSE
7.10E-05	FALSE	FALSE
9.73E-04	FALSE	FALSE
4.31E-04	FALSE	FALSE
0.003087	FALSE	FALSE
1.00E-04	FALSE	FALSE
0.006964	FALSE	TRUE
2.52E-04	FALSE	FALSE
1.79E-04	FALSE	FALSE
0.159504	FALSE	FALSE
3.13E-04	FALSE	FALSE
1.99E-04	FALSE	FALSE
2.19E-04	FALSE	FALSE
1.73E-04	FALSE	FALSE
1.31E-04	FALSE	FALSE
1.45E-04	FALSE	FALSE
0.002027	FALSE	FALSE
21.723796	FALSE	FALSE
5.34E-04	FALSE	FALSE
2.21E-04	FALSE	FALSE
5.30E-04	FALSE	FALSE

0.010689	FALSE	TRUE
0.006102	FALSE	FALSE
7.78E-04	FALSE	FALSE
5.90E-04	FALSE	FALSE
3.10E-04	FALSE	FALSE
3.67E-04	FALSE	FALSE
0.037069	FALSE	FALSE
1.13E-04	FALSE	FALSE
5.31E-04	FALSE	FALSE
0.001605	FALSE	FALSE
2.55E-04	FALSE	FALSE
0.005163	FALSE	FALSE
3.87E-04	FALSE	FALSE
0.00125	FALSE	FALSE
1.61E-04	FALSE	FALSE
4.02E-04	FALSE	FALSE
1.55E-04	FALSE	FALSE
0.00118	FALSE	FALSE
0.028729	FALSE	FALSE
7.10E-04	FALSE	TRUE
0.003299	FALSE	FALSE
0.001642	FALSE	FALSE
5.00E-04	FALSE	FALSE
3.10E-04	FALSE	FALSE
0.064026	FALSE	FALSE
7.30E-04	FALSE	FALSE
0.002814	FALSE	FALSE
0.009551	FALSE	FALSE
0.001114	FALSE	FALSE
1.07E-04	FALSE	FALSE
0.001984	FALSE	FALSE
0.002769	FALSE	FALSE
0.010053	FALSE	FALSE
0.030696	FALSE	FALSE
2.09E-04	FALSE	FALSE
1.30E-04	FALSE	FALSE
1.58E-04	FALSE	FALSE
4.94E-04	FALSE	FALSE
6.55E-04	FALSE	FALSE
6.50E-05	FALSE	FALSE
0.007669	FALSE	FALSE
1.29E-04	FALSE	FALSE
0.003137	FALSE	FALSE
5.29E-04	FALSE	FALSE
0.003253	FALSE	FALSE
0.008349	FALSE	FALSE
0.092759	FALSE	FALSE

0.015469	FALSE	FALSE
2.70E-04	FALSE	FALSE
4.29E-04	FALSE	FALSE
0.276638	FALSE	TRUE
0.008135	FALSE	FALSE
0.021288	FALSE	FALSE
0.075044	FALSE	FALSE
2.64E-04	FALSE	FALSE
8.68E-04	FALSE	FALSE
0.004835	FALSE	FALSE
0.00246	FALSE	FALSE
2.38E-04	FALSE	FALSE
0.001233	FALSE	FALSE
0.001218	FALSE	FALSE
0.002016	FALSE	FALSE
7.29E-04	FALSE	FALSE
0.005367	FALSE	FALSE
0.009978	FALSE	TRUE
0.021385	FALSE	FALSE
1.41E-04	FALSE	FALSE
1.31E-04	FALSE	FALSE
0.108061	FALSE	TRUE
1.79E-04	FALSE	FALSE
1.66E-04	FALSE	FALSE
1.12E-04	FALSE	FALSE
0.034744	FALSE	FALSE
0.055025	FALSE	FALSE
3.03E-04	FALSE	FALSE
0.041921	FALSE	FALSE
0.002241	FALSE	FALSE
0.032421	FALSE	TRUE
1.04E-04	FALSE	FALSE
2.62E-04	FALSE	FALSE
0.003156	FALSE	FALSE
0.001814	FALSE	FALSE
0.007904	FALSE	FALSE
0.015065	FALSE	FALSE
8.54E-04	FALSE	FALSE
0.001763	FALSE	FALSE
5.57E-04	FALSE	FALSE
0.002736	FALSE	FALSE
0.006599	FALSE	FALSE
0.116035	FALSE	FALSE
0.042124	FALSE	TRUE
0.003371	FALSE	FALSE
0.002775	FALSE	FALSE
2.15E-04	FALSE	FALSE

0.093646	FALSE	FALSE
0.001689	FALSE	TRUE
0.010765	FALSE	FALSE
8.77E-04	FALSE	FALSE
0.001828	FALSE	FALSE
0.031338	FALSE	FALSE
0.003783	FALSE	FALSE
0.002825	FALSE	FALSE
0.001107	FALSE	TRUE
2.12E-04	FALSE	FALSE
0.070108	FALSE	FALSE
0.005729	FALSE	FALSE
0.019421	FALSE	FALSE
2.58E-04	FALSE	FALSE
0.003636	FALSE	FALSE
0.00138	FALSE	FALSE
0.008747	FALSE	FALSE
6.05E-04	FALSE	FALSE
19.581072	FALSE	FALSE
0.002188	FALSE	FALSE
0.185509	FALSE	FALSE
0.026199	FALSE	FALSE
0.012741	FALSE	FALSE
3.34E-04	FALSE	FALSE
0.002312	FALSE	FALSE
0.001165	FALSE	FALSE
0.002088	FALSE	FALSE
0.002251	FALSE	FALSE
0.002549	FALSE	FALSE
0.001632	FALSE	FALSE
0.001971	FALSE	FALSE
0.059779	FALSE	FALSE
0.033369	FALSE	FALSE
0.005316	FALSE	FALSE
0.002707	FALSE	FALSE
0.001424	FALSE	FALSE
1.66E-04	FALSE	FALSE
0.021808	FALSE	FALSE
9.50E-05	FALSE	FALSE
0.001619	FALSE	FALSE
7.19E-04	FALSE	FALSE
0.002778	FALSE	FALSE
0.001856	FALSE	FALSE
1.25E-04	FALSE	FALSE
0.04906	TRUE	FALSE
9.60E-05	FALSE	FALSE
0.083838	FALSE	FALSE

0.00261	FALSE	FALSE
0.041589	TRUE	FALSE
0.050293	FALSE	FALSE
0.021683	FALSE	FALSE
0.001276	FALSE	FALSE
0.053669	FALSE	FALSE
0.006495	FALSE	FALSE
0.003632	FALSE	TRUE
0.004657	FALSE	FALSE
0.034353	FALSE	FALSE
